# Exon‐independent recruitment of SRSF1 is mediated by U1 snRNP stem‐loop 3

**DOI:** 10.15252/embj.2021107640

**Published:** 2021-11-15

**Authors:** Andrew M Jobbins, Sébastien Campagne, Robert Weinmeister, Christian M Lucas, Alison R Gosliga, Antoine Clery, Li Chen, Lucy P Eperon, Mark J Hodson, Andrew J Hudson, Frédéric H T Allain, Ian C Eperon

**Affiliations:** ^1^ Leicester Institute of Structural & Chemical Biology and Department of Molecular & Cell Biology University of Leicester Leicester UK; ^2^ Institute of Biochemistry ETH Zürich Switzerland; ^3^ Leicester Institute of Structural & Chemical Biology and Department of Chemistry University of Leicester Leicester UK; ^4^ Present address: MRC London Institute of Medical Sciences London UK; ^5^ Present address: Institute of Clinical Sciences Imperial College London London UK; ^6^ Present address: Inserm U1212 CNRS UMR5320 ARNA Laboratory Bordeaux Cedex France; ^7^ Present address: Institut für Industrielle Genetik Abt.(eilung) Systembiologie Universität Stuttgart Stuttgart Germany

**Keywords:** exon definition, RNA splicing, RNA–protein interaction, SRSF1, U1 snRNP, Chromatin, Transcription & Genomics, RNA Biology

## Abstract

SRSF1 protein and U1 snRNPs are closely connected splicing factors. They both stimulate exon inclusion, SRSF1 by binding to exonic splicing enhancer sequences (ESEs) and U1 snRNPs by binding to the downstream 5′ splice site (SS), and both factors affect 5′ SS selection. The binding of U1 snRNPs initiates spliceosome assembly, but SR proteins such as SRSF1 can in some cases substitute for it. The mechanistic basis of this relationship is poorly understood. We show here by single‐molecule methods that a single molecule of SRSF1 can be recruited by a U1 snRNP. This reaction is independent of exon sequences and separate from the U1‐independent process of binding to an ESE. Structural analysis and cross‐linking data show that SRSF1 contacts U1 snRNA stem‐loop 3, which is required for splicing. We suggest that the recruitment of SRSF1 to a U1 snRNP at a 5′SS is the basis for exon definition by U1 snRNP and might be one of the principal functions of U1 snRNPs in the core reactions of splicing in mammals.

## Graphical abstract

Single‐molecule analyses uncover recruitment of splicing factor SRSF1 to 5′‐splice sites through an interaction with U1 snRNP.

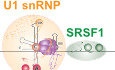

## Introduction

SRSF1 is one of the most‐studied regulators of alternative splicing. It is the archetypal member of the family of SR proteins, proteins that have one or two RNA recognition motif (RRM)‐type RNA‐binding domains and a C‐terminal RS domain rich in arginine‐serine dipeptides that can be phosphorylated extensively. The SR proteins have roles in a wide variety of processes, including transcription (Ji *et al*, 2013), nuclear export, translation and nonsense‐mediated decay (Long & Caceres, 2009; Maslon *et al*, 2014; Muller‐McNicoll *et al*, 2016), but it is for their roles in alternative splicing that they are best known. SRSF1 in particular activates or represses the inclusion of hundreds of exons (Pandit *et al*, 2013; Anczukow *et al*, 2015; Bradley *et al*, 2015), and this activity is thought to be the primary reason why it is both essential (Wang *et al*, 1996; Longman *et al*, 2000; Lin *et al*, 2005b) and an oncoprotein (Das & Krainer, 2014). It also plays an essential but poorly understood role in the core reactions of splicing.

Three key observations made in 1993 underpin the prevailing model for the actions of SR proteins in splicing. SR proteins were found to bind purine‐rich enhancer sequences (Lavigueur *et al*, 1993; Sun *et al*, 1993), to interact with the core splicing components U2AF35 and U1‐70K (Wu & Maniatis, 1993) and, for SRSF1 in particular, to stabilize the binding of U1 snRNP complexes at 5′ splice sites (5′SSs) (Eperon *et al*, 1993). The resultant model for activation of splicing by SRSF1 is that it binds exonic splicing enhancer sequences (ESEs) (Graveley & Maniatis, 1998; Sanford *et al*, 2009; Clery *et al*, 2013; Pandit *et al*, 2013; Ray *et al*, 2013; Anczukow *et al*, 2015) and then recruits limiting splicing factors such as U1 snRNPs or U2‐associated proteins to 5′ or 3′ splice sites, respectively, by direct protein–protein interactions that stabilize the association of the splicing factor with the pre‐mRNA (Eperon *et al*, 1993; Lavigueur *et al*, 1993; Wu & Maniatis, 1993; Amrein *et al*, 1994; Kohtz *et al*, 1994; Staknis & Reed, 1994; Jamison *et al*, 1995; Tarn & Steitz, 1995; Wang *et al*, 1995; Cao & Garcia‐Blanco, 1998; Graveley *et al*, 2001; Martins de Araujo *et al*, 2009; Cho *et al*, 2011; Smith *et al*, 2014; Akerman *et al*, 2015). This model is commonly depicted in cartoon representations of splicing (Will & Luhrmann, 2011; Lee & Rio, 2015; Wahl & Luhrmann, 2015). Moreover, this model is compatible with the two properties that led to the isolation of SRSF1 originally: its ability to modulate 5′SS selection and its ability to restore splicing activity to S100 cytoplasmic extracts (Ge & Manley, 1990; Krainer *et al*, 1990a, 1990b, 1991; Ge *et al*, 1991), since the latter could be accounted for by the ability to enhance the binding of scarce components and depended on exon sequences (Chandler *et al*, 1997; Mayeda *et al*, 1999). We inferred from recent single‐molecule experiments that the activity of a mammalian ESE is limited by a low probability of transient occupancy by SRSF1, and, using chimeric RNA with non‐RNA linkers, that the association of bound SRSF1 with 3′ splice site factors involves direct protein–protein interactions mediated by 3D diffusion; this stabilizes the binding of the successful molecule of SRSF1 (Jobbins *et al*, 2018).

Several observations suggest that SRSF1 has other activities. First, SRSF1 was shown to stimulate splicing in S100 extracts of truncated pre‐mRNA substrates that contained only 1–3 nt of the 5′ exon and lacked a 3′ exon (Hertel & Maniatis, 1999; Zhu & Krainer, 2000). Second, whereas the activation of splicing by SR proteins requires phosphorylation of their RS domains (Caceres & Krainer, 1993; Zuo & Manley, 1993; Mermoud *et al*, 1994; Roscigno & Garcia‐Blanco, 1995; Cao *et al*, 1997; Xiao & Manley, 1997, 1998; Graveley & Maniatis, 1998; Zhu & Krainer, 2000; Cartegni & Krainer, 2003; Shen & Green, 2006), the catalytic reactions of splicing require dephosphorylation of the RS domains of SR proteins and specifically SRSF1 (Mermoud *et al*, 1992, 1994; Cao *et al*, 1997; Xiao & Manley, 1998). This suggests that SRSF1 has an additional function in splicing. Third, cross‐linking has shown that the RS domain of SRSF1 contacts the pre‐mRNA in mature spliceosomal complexes B and C (Shen *et al*, 2004). Attempts to distinguish the putative roles of SRSF1 in splicing from those in alternative splicing by identifying the domains responsible have foundered, possibly because both of the two original assays might have been assaying the recruitment activity; alternatively, multiple domains might be involved in interactions or the RS domain might be involved in autoregulation (Caceres & Krainer, 1993; Zuo & Manley, 1993; Wang & Manley, 1995; Caceres *et al*, 1997; Eperon *et al*, 2000; Zhu & Krainer, 2000; Lin *et al*, 2005b; Shaw *et al*, 2007; Cho *et al*, 2011; Serrano *et al*, 2016).

Establishing whether SRSF1 really is engaged in the constitutive reactions of splicing is made particularly difficult because the SR proteins are found at variable and non‐stoichiometric levels in spliceosome preparations (Schmidt *et al*, 2014). This is not unexpected, given that SRSF1 can bind to a wide range of pre‐mRNA sequences and might also interact by electrostatic interactions of its RS domain. Binding by multiple molecules that are involved in different interactions precludes the use of conventional methods for resolving different events. Here, we have unravelled this complexity by the use of single‐molecule methods and reveal that SRSF1 is associated in a 1:1 complex with U1 snRNPs, interacts with stem‐loop 3 of U1 snRNA and is recruited by U1 snRNPs to 5′ splice sites. This suggests a function for U1 snRNPs in splicing reactions, which is otherwise still missing, and explains why 5′ splice sites can act like ESEs in exon definition (Kreivi *et al*, 1991; Hwang & Cohen, 1997; Fernandez Alanis *et al*, 2012; Braun *et al*, 2018; Singh & Singh, 2019; Erkelenz *et al*, 2021).

## Results

### Detection of single molecules in complexes

**Figure 1:**
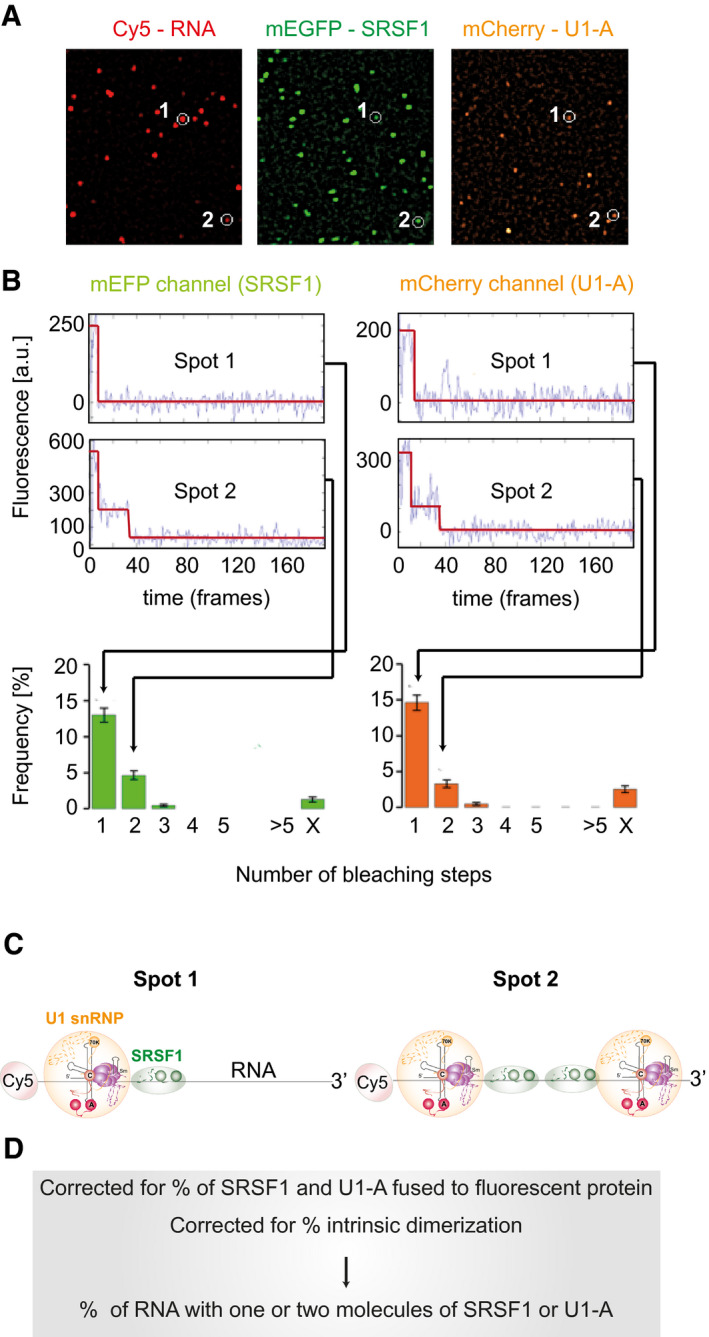
Representation of methods used for determining the frequencies with which 1, 2, etc., molecules of fluorescent protein are associated with pre‐mRNA After incubation of Cy5‐labelled pre‐mRNA in a nuclear extract expressing mEGFP‐SRSF1 and mCherry‐U1A, samples are diluted and applied to a coverslip. Fluorescent images are acquired at time frames of 20–100 ms from excitation successively at 633 nm (to detect Cy5), 561 nm (to detect mCherry) and 488 nm (to detect mEGFP), and at each stage, collection continues until all the molecules have bleached. Cy5‐labelled RNA molecules (red) and mEGFP (green) and mCherry (orange) molecules are detected as spots in the composite images from each laser (Materials and Methods). Colocalized spots are identified (white circles); the other spots are not colocalized.Time courses of fluorescence are analysed to detect bleaching of the fluorophores in each spot. Two hypothetical molecular complexes are followed, one in which a molecule of RNA is associated with one fluorescent protein of each type (Spot 1) and the other in which it is associated with two fluorescent proteins of each type (Spot 2; either the result of binding to another site in the pre‐mRNA or dimerization). Each spot in which mEGFP bleached in one step contributes to the frequency of the *N* = 1 class; each spot in which it bleached with two steps is added to the *N* = 2 class, etc. The proportions of RNA spots associated with mEGFP signals that bleach in one, two, etc., steps are shown as a frequency histogram.§
Inferred compositions of Spot 1 and Spot 2. Note that the positions and mechanisms of binding are not revealed by this method. The 5′ Cy5 fluorophore on the pre‐mRNA is highlighted in red; the U1 snRNP is represented by an orange disc, containing a diagram of the U1 RNA secondary structure with solid spheres representing the structured domains of the Sm proteins and the U1‐specific proteins, lines showing extended regions of the protein structures and dashed lines showing unstructured regions, since it was detected by mCherry‐U1A fluorescence; SRSF1 is represented by a green ellipse, containing two darker green circles representing the two RNA‐binding domains, since it was detected by mEGFP fluorescence.The frequency of complexes containing one, two, etc., molecules of SRSF1 and U1 snRNPs is calculated by taking into account the relative levels of the endogenous, untagged proteins and the frequency of intrinsic dimerization of each protein.

To detect single complexes of SRSF1 with pre‐mRNA, Cy5‐labelled pre‐mRNA was incubated in splicing conditions in nuclear extracts prepared from cells expressing functional monomeric mEGFP‐SRSF1 (Table 1; Appendix Fig S1; (Sleeman *et al*, 1998; Ellis *et al*, 2008)). In most of the experiments shown here, the incubations were done in the presence of ATP and a 2′‐*O*‐methyl oligonucleotide complementary to U6 snRNA that caused spliceosome assembly to stall at complex A (Donmez *et al*, 2007). Nuclear extract (NE) 3 contained mCherry‐U1A as well, which associates with pre‐mRNA only as part of the U1 snRNP (Hodson *et al*, 2012). The reaction mixtures were diluted and then applied to coverslips (Fig 1). Complexes adsorb directly to the glass surface and are stable indefinitely. Total internal reflection fluorescence microscopy was used to locate surface‐bound RNA molecules, and then, protein fluorescence was recorded until all the fluorophores had bleached. Colocalized spots containing Cy5 and mEGFP or mCherry were identified, and the number of steps in which the protein fluorophores bleached was recorded for each complex. Each step is caused by the bleaching of a single molecule. The number of complexes in which bleaching had taken place in 1, 2, etc. steps is plotted as a percentage of the number of RNA molecules detected (Fig 1), and the absolute number of complexes is given above each bar.

**Table 1:** Concentrations and properties of expressed fluorescent proteins.

	NE1	NE2	NE3	
	mEGFP‐SRSF1	mEGFP‐SRSF1	mEGFP‐SRSF1	mCherry‐U1A
Concentration in extract (µM)	1.5	0.72	n.d.	n.d.
% tagged:endogenous	90:10	59:41	58:42	57:43
Molecules bleaching in 2 vs 1 steps, +ATP	32:129	9:118	18:138	15:124

Concentrations were calculated from Western blotting of nuclear extract alongside recombinant protein; the percentages labelled or unlabelled (endogenous) were also calculated by Western blotting; the numbers of molecules recorded as bleaching in one or two steps when not associated with exogenous RNA are shown. n.d., not determined.

### Stable binding of SRSF1 depends on U1 snRNP and 5′ splice sites

**Figure 2:**
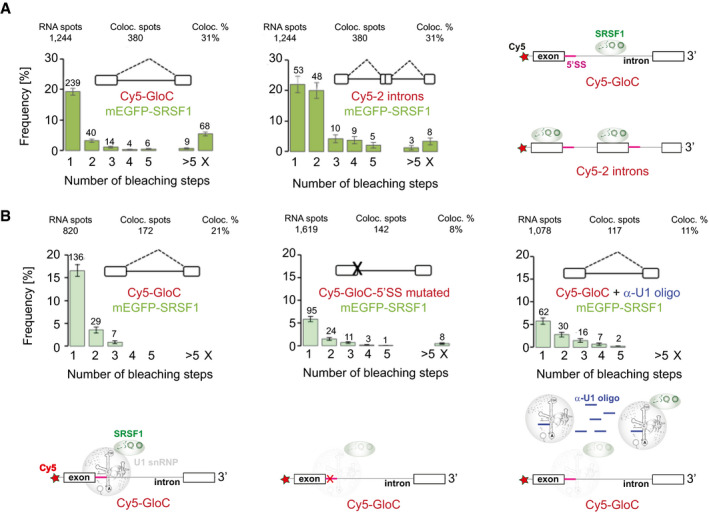
Dependence of stoichiometric SRSF1 association with pre‐mRNA on 5′ splice sites and U1 snRNA Two‐way colocalization of mEGFP‐SRSF1 with Cy5‐pre‐mRNA containing one (left) or two (right) introns. In this experiment, nuclear extract (NE) 1 was used. The SRSF1 histograms are colour‐coded in three shades of green to indicate the extract used in each experiment shown. The number above each bar indicates the number of complexes in which complete bleaching was achieved in 1, 2, 3, etc., steps, and hence the number of complexes in which there were 1, 2, 3, etc., molecules of mEGFP‐SRSF1. > 5 refers to complexes where a higher number of bleaching steps were measured; X represents complexes where the number could not be determined. RNA spots refer to numbers of molecules of Cy5‐labelled pre‐mRNA detected in the fields acquired; Coloc. spots is the number of these associated with mEGFP‐SRSF1, and Coloc. % is the resulting percentage. The error bars are the square root of the variance of the binomial probability that an RNA spot will be associated with the given number of protein bleaching steps. The pre‐mRNA is represented above the histograms. For globin‐derived pre‐mRNA, the white boxes represent exons and the line in between is the intron. Potential splicing pathways are shown as dashed lines. The pre‐mRNA with two introns was formed by duplication of GloC sequences. The cartoons on the right show the inferences drawn from the histograms. The pre‐mRNA molecules are shown in grey, with the Cy5 label shown as a red star and 5′ splice sites as purple segments. From the histogram on the left we infer that, in those complexes where a molecule of pre‐mRNA was associated with SRSF1, only one molecule of SRSF1 (green) was present, while the histogram on the right shows that about half of the complexes of pre‐mRNA containing two introns were associated with two molecules of SRSF1.Two‐way colocalization of mEGFP‐SRSF1 with Cy5‐pre‐mRNA showing dependence of SRSF1 binding on 5′SS and U1 snRNP. Complexes formed in NE2 on GloC pre‐mRNA (left), a derivative with an inactivating mutation in the 5′SS mutant (centre) and on GloC in the presence of an additional 2′‐*O*‐methyl oligonucleotide complementary to the 5′ end of U1 snRNA (right). The last distribution is geometric (χ^2^, *P_geo_* = 0.72). Error bars are as described in (A). The interpretative cartoons underneath are based on the observed dependence of SFSF1 binding on U1 snRNPs and represent the possibility that in many cases, SRSF1 binding might be mediated by U1 snRNPs. SRSF1 is in green as in Fig 1, and the U1 snRNPs are in grey since they were not detected directly in this experiment. Paler colours indicate reduced levels of a labelled protein in the complex.

We reported recently that most of the molecules of a pre‐mRNA containing one intron with a single strong 5′ splice site (GloC) were associated with just one molecule of mEGFP‐SRSF1 in complex A conditions (Jobbins *et al*, 2018) (Fig 2A), despite the presence of a number of potential binding sites in the exon (Schaal & Maniatis, 1999). To test whether this surprisingly restricted binding was connected with splicing, a second pre‐mRNA was tested that contained two introns, derived by duplication of the sequences in GloC (Hodson *et al*, 2012). The distribution expected if all SRSF1‐associated pre‐mRNA molecules were bound by either one or two molecules of mEGFP‐SRSF1 in this extract (NE1) is shown in Appendix Fig S2. The observed distribution (Fig 2A) was intermediate between the two canonical distributions. Estimations of the proportions of the two classes suggest that approximately 55% of the SRSF1‐pre‐mRNA complexes contained one molecule of SRSF1 and 45% contained two molecules of SRSF1. In previous work, we showed that under these conditions about 40 and 60% of the same pre‐mRNA was colocalized with one or two U1 snRNPs, respectively (Hodson *et al*, 2012), whereas only one U2 snRNP‐containing complex was detected (Chen *et al*, 2017). The results in Fig 2A raise the possibility that SRSF1 was associated with U1 snRNPs in spliceosomal complex A.

The link to U1 snRNPs was tested by repeating the experiment with a pre‐mRNA containing a mutant 5′ splice site that substantially reduces U1 snRNP association (Hodson *et al*, 2012). The fraction of pre‐mRNA molecules associated with SRSF1 was halved in the mutant (Fig 2B). A very similar change was seen when the binding of U1 snRNP in NE2 was blocked by a complementary 2′‐*O*‐methyl oligonucleotide (Hodson *et al*, 2012) (Fig 2B). The residual distributions in these conditions appear to represent alternative modes of association with pre‐mRNA molecules that are unable to form complex A (see Discussion). We conclude that U1 snRNP binding to 5′SSs directly or indirectly determines whether SRSF1 binds with a fixed stoichiometry.

The strong dependence on U1 snRNPs and the 5′SS was seen also when experiments were done after ATP depletion, in conditions used to form complex E, often taken as a model for the earliest interactions that form prior to complex A or splice site selection (Michaud & Reed, 1991, 1993; Jamison *et al*, 1992; Lim & Hertel, 2004; Kotlajich *et al*, 2009). Under these conditions, SRSF1 is substantially hypophosphorylated (Appendix Fig S1). However, compared with complex A conditions, there was an increase in the proportion of GloC pre‐mRNA molecules associated with two molecules of mEGFP‐SRSF1 and a noticeable increase in larger complexes that are not U1‐dependent (Appendix Fig S3). This is consistent with the co‐existence of U1 snRNP‐dependent recruitment and sequence‐independent interactions made by the positively charged hypophosphorylated RS domains. Non‐specific binding to the pre‐mRNA by an unphosphorylated RS domain has been observed previously (Cho *et al*, 2011). The involvement of phosphorylation was confirmed by the inclusion of phosphatase inhibitors. In this case, the pattern of SRSF1 association approached more closely that seen in complex A, and the complexes with very large numbers of molecules of SRSF1 were much reduced (Appendix Fig S3D). These results are very similar to those we observed previously with U2AF65 and U2 snRNP (Chen *et al*, 2017), and confirm that complex I, formed in the presence of phosphatase inhibitors, might be a better model for early splicing complexes in higher eukaryotes than complex E (Chen *et al*, 2017).

### SRSF1 binds with U1 snRNP

The observation that SRSF1 recruitment is determined by U1 snRNP does not necessarily mean that both factors are present on the pre‐mRNA concurrently. Specifically, U1 snRNP might recruit SRSF1 and then dissociate. To test this, colocalization studies were done with a nuclear extract (NE3) containing mEGFP‐SRSF1 and mCherry‐U1A, which only associates with pre‐mRNA as part of the U1 snRNP (Hodson *et al*, 2012). In both cases, the labelled component was expressed at about the same level as the endogenous protein (Table 1; Appendix Fig S1). Both proteins were predominantly found as single molecules on the pre‐mRNA (Fig 2B), although there was a higher proportion of complexes with mEGFP‐SRSF1 than with mCherry‐U1A containing two labelled proteins. When pre‐mRNA complexes containing both protein fluorophores (three‐way colocalization) were examined, the distribution of each was similar to that observed in the two‐way colocalization (Fig 2B, lower panel; χ^2^, *P_SRSF1 3‐way = 2‐way_* = 0.93; χ^2^, *P_U1A 3‐way = 2‐way_* = 0.76) and, significantly, these complexes were found at about half the frequency of complexes that were selected for the presence of one protein without regard to the other. This result is exactly what would have been expected if every one of the complexes containing labelled SRSF1 contained U1 snRNP (either labelled or unlabelled), and vice versa.

### SRSF1 is recruited to pre‐mRNA without exons

**Figure 3:**
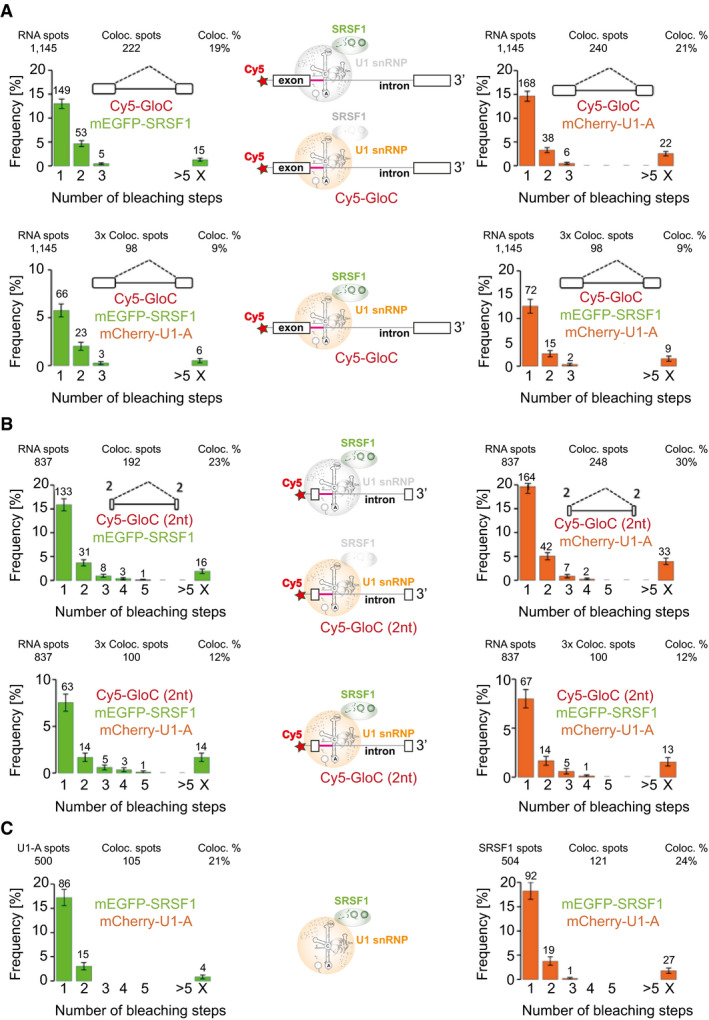
SRSF1 and U1 snRNPs are present concurrently in individual pre‐mRNA complexes even in the absence of exons Complexes formed on GloC in NE3, containing labelled mEGFP‐SRSF1 and mCherry‐U1A, in which the distribution of mEGFP‐SRSF1 (left) and mCherry‐U1A (right) in two‐way colocalization with pre‐mRNA is shown; below, the respective distributions of mEGFP‐SRSF1 and mCherry‐U1A on 3‐way colocalized complexes. The error bars in the histograms are the square root of the variance of the binomial probability that an RNA spot will be associated with the given number of protein bleaching steps. The interpretative cartoons in the centre are based on the model in Fig 2B. The top cartoon, based on the upper‐left histogram, shows the majority of SRSF1‐pre‐mRNA complexes contain a single molecule of SRSF1, while U1 snRNPs are in grey to indicate that the data relate only to SRSF1; the lower cartoon, based on the upper‐right histogram, shows that most U1/pre‐mRNA complexes contain only one U1 snRNP, while SRSF1 is in grey to indicate that the data relate only to U1 snRNPs; the third cartoon, based on the lower two histograms, shows that complexes shown to contain both U1 snRNPs and SRSF1 contain only a single molecule of each componentt.Complexes formed in NE3, as in (A) but with GloC pre‐mRNA containing exons of only 2 nts. The error bars and interpretative cartoons are as described for panel (A).Frequencies of mEGFP‐SRSF1 and mCherry‐U1A in two‐way colocalized complexes formed in the absence of pre‐mRNA. Error bars are as described in (A). The inferred complex is shown in the cartoon.

**Figure 4:**
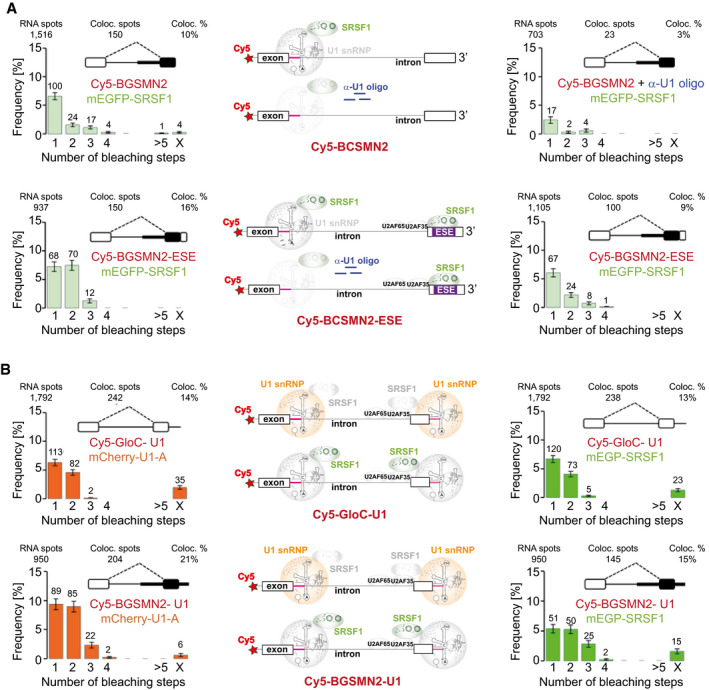
A 3′‐terminal ESE and a 3′‐terminal 5′SS both enable recruitment of an additional and independent molecule of SRSF1 Effects of 3′‐terminal ESE repeats. Top left, two‐way colocalization of complexes formed in NE2 on BGSMN2, a chimeric globin/SMN2 pre‐mRNA. The heavy black line and box indicate the intron and exon sequences of SMN2 exon 7. Top right, the effects of inclusion of a 2′‐*O*‐methyl oligonucleotide complementary to the 5′ end of U1 snRNA. The interpretative cartoons (top centre) show (upper) that most SRSF1/pre‐mRNA complexes contain a single molecule of SRSF1 and (lower) that the proportion of SRSF1‐associated pre‐mRNA molecules was reduced sharply by occlusion of the 5′ end of U1 snRNA by the oligonucleotides. Below, the corresponding experiments after addition of four tandem repeats of a strong ESE to the 3′ terminus (Jobbins *et al*, 2018). Bottom left, most SRSF1/pre‐mRNA complexes contain two molecules of SRS1. In the interpretative cartoon in the centre (upper), this is shown by association of one SRSF1 with the U1 snRNP and the other with the ESEs (blue). We have shown previously that the activity of ESEs is limited by their occupancy, consistent with transient binding by SRSF1 and stabilization of one molecule by direct interactions based on diffusional encounters with splice site‐associated factors, since their effects are not blocked by the incorporation of intervening non‐RNA linkers (Jobbins *et al*, 2018). Bottom right, double occupancy of the pre‐mRNA was greatly reduced by occlusion of the 5′ end of U1 snRNA, suggesting that the U1‐dependent but not ESE‐dependent association of SRSF1 was lost (lower cartoon). The error bars in the histograms are the square root of the variance of the binomial probability that an RNA spot will be associated with the given number of protein bleaching steps.Effects of a 3′‐terminal 5′ SS. Two‐way colocalizations of Cy5‐pre‐mRNA with mCherry‐U1A (left) or mEGFP‐SRSF1 (right) of complexes formed in NE3 on GloC pre‐mRNA after addition of a 3′‐terminal 5′SS (upper panel; c.f. Fig 3B) and on BGSMN2 with a 3′‐terminal 5′SS (lower panel; c.f. Fig 4A and Jobbins *et al*, (Jobbins *et al*, 2018)). The interpretative cartoons in both cases show that the majority of pre‐mRNA/U1 complexes and pre‐mRNA/SRSF1 complexes (upper and lower members of each pair) contain two molecules of U1 and SRSF1, respectively. The presence of the components shaded in grey is inferred from the preceding experiments. Error bars are as described in (A).

The concurrent colocalization might be attributed to mutual stabilization resulting from the binding of SRSF1 to an ESE and interactions with U1 snRNP (Jobbins *et al*, 2018). This possibility was tested with a pre‐mRNA in which the exons contained only approximately 2 nucleotides (Fig 3C). These transcripts, as with those in Fig 4 also, were labelled using 5′ Cy5‐maleimide, which was used previously for studies on spliceosome assembly (Ohrt *et al*, 2012). The substitution of Cy5 for the normal cap produces only relatively small (20–25%) reductions in the rates of splicing, and complexes accumulate within the times used for incubations (Appendix Fig S4).

The colocalization percentages showed that there was no reduction in the binding of either SRSF1 or U1 snRNPs, and, again, concurrent detection was observed in around one‐half of the complexes. Strikingly, whereas the proportions of complexes with one or two molecules of mCherry‐U1A are the same on this pre‐mRNA as with GloC (χ^2^, *P_U1A 2‐way 2C = 2B_* = 0.49), there are proportionately fewer complexes containing two molecules of mEGFP‐SRSF1 than was seen with GloC (χ^2^, *P_SRSF1 2‐way 2C = 2B_* = 0.033), and the distribution of complexes with one or two molecules of mEGFP‐SRSF1 now matches that observed for mCherry‐U1A (χ^2^, *P_2C 2‐way SRSF1 = U1A_* = 0.64). We infer that the complexes with GloC contain some pre‐mRNAs in which SRSF1 binds weakly via the exon sequences, but that with the short exons, recruitment is associated primarily with U1 snRNP binding. We conclude that SRSF1 recruitment with U1 snRNP does not require exonic splicing enhancer sequences.

### The association of U1 snRNP and SRSF1 does not require pre‐mRNA

The strong link between U1 snRNP and SRSF1 association with the pre‐mRNA suggested the possibility that interactions between the two molecules might occur even prior to or in the absence of binding to pre‐mRNA. Around 24% of mEGFP‐SRSF1 molecules and 21% of mCherry‐U1A molecules were associated with mCherry‐U1A and mEGFP‐SRSF1, respectively, in the absence of pre‐mRNA (Fig 3D). Given the fraction of each protein that was labelled, we infer that around half the molecules in each case were present in a U1:SRSF1 heterodimer. Interestingly, as with the results with the 2 nt exon (Fig 3C), the distributions of complexes with one or two molecules of mEGFP‐SRSF1 and mCherry‐U1A are the same (χ^2^, *P_2C SRSF1 = U1A_* = 0.55). When the experiments were repeated in the presence of ribonuclease, to remove residual pre‐mRNA, the level of association was reduced but was still substantial (Appendix Fig S5). Previous analyses had shown that the U1‐70K protein can interact directly with SRSF1, via either the RS or RRM domains (Kohtz *et al*, 1994; Xiao & Manley, 1997; Cho *et al*, 2011), and that they are in close proximity in cells even after ribonuclease treatment (Ellis *et al*, 2008). Our results are consistent with the possibility that a significant proportion of each protein is present in a heterodimer in functional splicing conditions.

### Recruitment of SRSF1 via U1 snRNP or an ESE is separate processes

The recruitment of a single molecule of SRSF1 by U1 snRNP is at odds with the conventional picture in which SRSF1 binds independently to ESE sequences in a pre‐mRNA and then stabilizes the binding of splicing factors. To test whether our observations have revealed a second and different mode of SRSF1 binding, we used a chimeric pre‐mRNA (BGSMN2) in which the 5′ part was derived from GloC and the 3′ part from SMN2 exon7, which does not contain an ESE bound by SRSF1. The splicing of this was enhanced by the addition of a 3′‐terminal SRSF1‐dependent ESE (BGSMN2 + ESE‐Ax4), which increases the association of U2AF35, U2AF65 and U2 snRNPs (Jobbins *et al*, 2018). In the absence of the ESE, the predominant complexes formed in complex A conditions contained one molecule of mEGFP‐SRSF1, which was U1 snRNP‐dependent (Fig 4A). The addition of the ESE caused an upsurge in molecules associated with two molecules of mEGFP‐SRSF1, such that 100% occupancy by two molecules of SRSF1 could not be excluded (χ^2^, *P_2_*_SRSF1_ = 0.26). In this case, addition of the anti‐U1 oligonucleotide substantially reduced the frequency of bleaching in two steps from approximate equality with bleaching in one step to about one‐third of the value. This indicates a major reduction in complexes containing two molecules of SRSF1, but the proportion cannot be estimated because of the background associated with U1 sequestration (c.f. Fig 2B). We conclude that the ESE sequence acted independently from the U1 snRNP to recruit an additional molecule of SRSF1 and that U1‐dependent recruitment is a separate phenomenon.

### A downstream 5′SS recruits SRSF1 and, like an ESE, recruits U2 snRNP to the 3′SS

A poorly understood property of U1 snRNP is the ability of a 5′SS at the 3′ end of an exon to stimulate splicing via an increase in U2AF65 binding to the upstream 3′SS (Grabowski *et al*, 1991; Kreivi *et al*, 1991; Hoffman & Grabowski, 1992; Cote *et al*, 1995; Izquierdo *et al*, 2005; Singh *et al*, 2007; Palacino *et al*, 2015). This is fundamental to exon definition models of mammalian splicing (Robberson *et al*, 1990; Sterner *et al*, 1996). Although the mechanisms involved have not been described, and U1 snRNPs do not appear to interact directly with U2AF, this property of U1 snRNP is similar to that of an ESE. The data described above suggest that the underlying mechanism might be the recruitment of SRSF1 by U1 snRNP.

To test this hypothesis, a consensus 5′SS sequence was attached to the 3′ end of both GloC and BGSMN2 pre‐mRNAs. This increased splicing of BGSMN2 more than the addition of two repeats of a strong ESE (BGSMN2‐ESEx2; Appendix Fig S6) (Jobbins *et al*, 2018). There was a significant increase in the proportion of pre‐mRNA‐associated complexes containing two U1 snRNPs (Fig 4B), which was estimated for GloC at 92% (χ^2^, *P_2_*_U1_ = 0.095) and for BGSMN2 at 100% (χ^2^, *P_2_*_U1_ = 0.39). In accordance with the hypothesis, there was also a corresponding increase in the proportion of complexes containing two or more molecules of SRSF1 (Fig 4B), estimated at 67% for GloC.

A characteristic property of exon definition is the recruitment of components to the upstream 3′SS. To test whether the appended 5′SS was involved in exon definition reactions, its effect on the recruitment of 3′SS components was analysed by single‐molecule methods with nuclear extracts from cells expressing either mEGFP‐U2B′′ or both mEGFP‐U2AF65 and mCherry‐U2AF65 (Chen *et al*, 2017) and compared with contemporaneous results with the same extracts on BGSMN2 and a derivative carrying four repeats of an SRSF1‐dependent enhancer (Jobbins *et al*, 2018). For all three components, the 3′‐terminal 5′SS resulted in an increase in the proportion of transcripts bound, the increase being greatest for U2 snRNP and least striking for U2AF65 (Table 2; Appendix Fig S7). This is consistent with previous results showing that U2 snRNP recruitment is affected more strongly than U2AF35 and U2AF65 by the difference in sequence between SMN1 and SMN2 (Chen *et al*, 2017) or the presence of *cis*‐ or *trans*‐acting enhancers (Martins de Araujo *et al*, 2009; Smith *et al*, 2014; Jobbins *et al*, 2018). Importantly, only a single molecule of each component was bound, showing that the 3′‐terminal U1 snRNP/SRSF1 complex did not recruit extraneous or non‐specific components. We conclude that the 3′‐terminal 5′SS mediates increases in the recruitment of single molecules of U2AF and U2 snRNPs equivalent to those produced by four repeats of a strong enhancer, showing that it is functional in the key reaction of exon definition, and that U1 snRNP binding to it recruits a molecule of SRSF1.

**Table 2:** Effects of a downstream 5′SS or ESE sequences on recruitment of 3′SS components.

	mEGFP‐U2AF65	mCherry‐U2AF35	mEGFP‐U2B′′
BGSMN2	10	12	10
BGSMN2‐ESEx4	16	25	25
BGSMN2‐U1	14	20	27

Values show the percentage of pre‐mRNA molecules in each single‐molecule experiment colocalized with labelled U2AF and U2 snRNP, as indicated. The two components of U2AF were co‐expressed and measurements made in the same experiment; mEGFP‐U2B′′ was expressed separately (Chen *et al*, 2017). BGSMN2‐ESEx4 is BGSMN2 with a 3′ extension containing four repeats of the enhancer used in BGSMN2‐ESE (Fig 4A). The data for this and BGSMN2 are from Jobbins *et al*, (2018). The data for BGSMN2‐U1 are shown in Appendix Fig S7. For each of the two extracts, the experiments with all three pre‐mRNA substrates were done at the same time in the same nuclear extract.

### SRSF1 binds directly to U1 snRNP on stem‐loop 3

**Figure 5:**
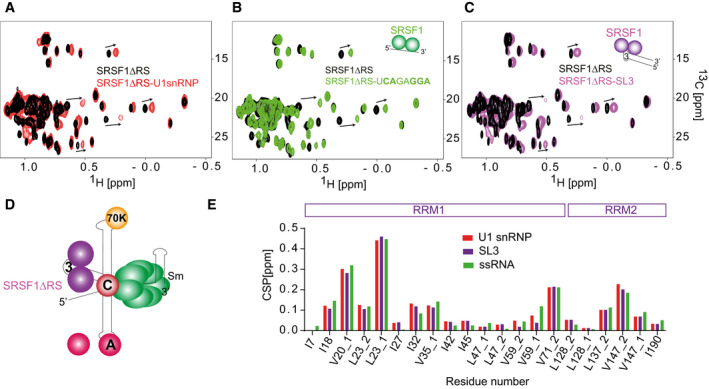
Direct interaction between SRSF1 and U1 snRNP Overlay of the 2D ^13^C‐^1^H HMQC of ^13^C ILV‐labelled SRSF1∆RS before and after addition of 1 molar equivalent of *in vitro* reconstituted U1 snRNP.Overlay of the 2D ^13^C‐^1^H HMQC of ^13^C ILV‐labelled SRSF1∆RS before and after addition of 1 molar equivalent of the ssRNA target of SRSF1.Overlay of the 2D ^13^C‐^1^H HMQC of ^13^C ILV‐labelled SRSF1ΔRS before and after addition of 1 molar equivalent of U1 snRNA stem‐loop 3.Schematic representation of SRSF1ΔRS bound on U1 snRNP.Bar plot showing the chemical shift perturbations of the SRSF1 methyl groups when the protein was titrated with U1 snRNP, ssRNA or U1 snRNA SL3.

The results so far have shown that SRSF1 forms a heterodimer with U1 snRNP and is recruited by it to 5′SSs. To test whether the interaction between U1 snRNP and SRSF1 could be direct, we titrated ^13^C ILV‐labelled SRSF1∆RS with *in vitro* reconstituted U1 snRNP using NMR spectroscopy (Fig 5A), as we did previously for other splicing factors (Campagne *et al*, 2019, 2021; Jutzi *et al*, 2020). SRSF1∆RS lacks the RS domain (amino acids 198–248) and is more soluble than the full‐length protein. It is fully active in 5′SS selection and can restore splicing of pre‐mRNAs with strong 3′SS in S100 extracts (Eperon *et al*, 2000; Zhu & Krainer, 2000). Single‐molecule analysis in complex A conditions showed that the predominant complexes contained a single molecule of SRSF1∆RS on GloC RNA and two on BGSMN2+ESE‐Ax4, respectively (Appendix Fig S8). Thus, the truncated protein maintains at least some of the U1 snRNP association properties of full‐length SRSF1. Surprisingly, the methyl groups of SRSF1∆RS experienced chemical shift perturbations (CSP) upon addition of *in vitro* reconstituted U1 snRNP. These CSP saturated at ratio 1:1 (SRSF1∆RS:U1snRNP) and were very similar to the ones observed previously when SRSF1∆RS was titrated with its single‐stranded RNA (ssRNA) target sequence containing a CA and a GGA‐binding site for RRM1 and RRM2, respectively (Fig 5B). This suggests that SRSF1∆RS can bind U1 snRNA in a similar fashion. The analysis of the structure of U1 snRNP (Pomeranz Krummel *et al*, 2009; Kondo *et al*, 2015) and the sequence of the U1 snRNA revealed that stem‐loop 3, which is solvent accessible and free of any U1‐specific protein, contains the CA and GGA motifs with the CA being in the loop and the GGA motif in the adjacent double‐stranded region (Clery *et al*, 2013, 2021). The possibility that stem‐loop 3 would be the target site for SRSF1∆RS was tested by using U1 snRNA stem‐loop 3 alone. This reproduced the same methyl CSP (Fig 5C), indicating that SRSF1∆RS binds U1 snRNP stem‐loop 3 with RRM1 bound to the CA motif at the 5′ side of the loop and RRM2 binding the GGA sequence in the 3′ side of stem (Fig 5D and E).

**Figure 6:**
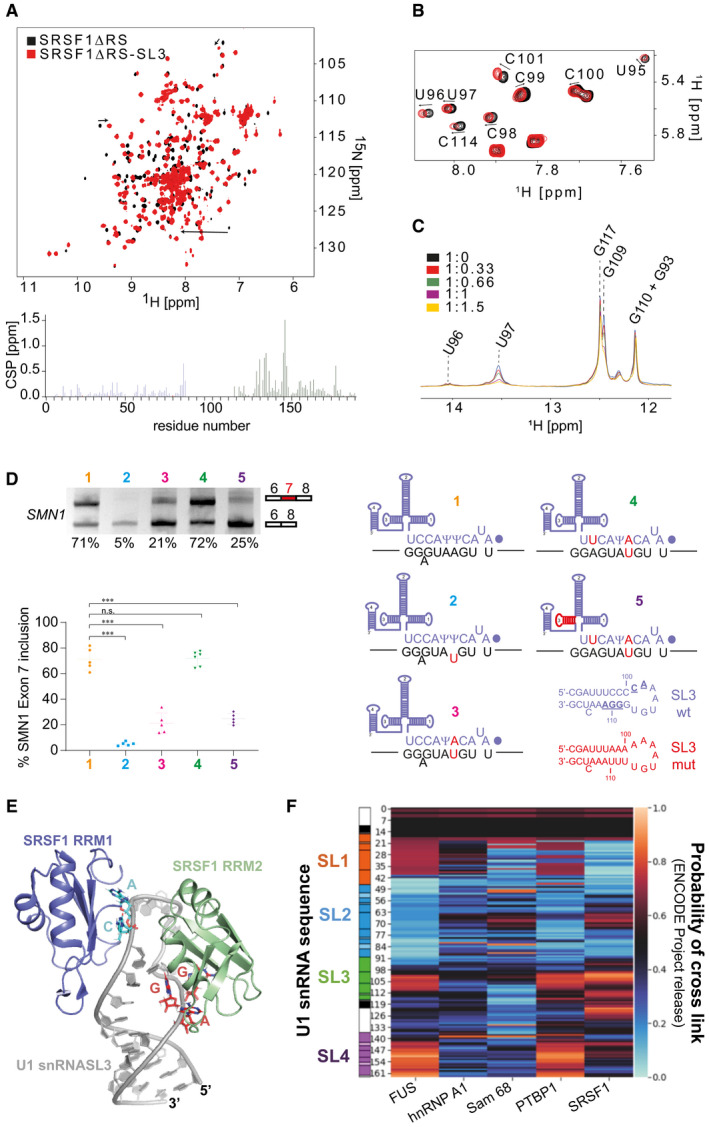
SRSF1∆RS binds U1 stem‐loop 3 SL3 at nucleotides that are essential for the function of U1 snRNA Overlay of the 2D ^1^H‐^15^N HSQC spectra of SRSF1∆RS before (black) and after addition of one equimolar amount of U1 snRNA SL3 (red). Bar plot showing the chemical shift perturbations (CSP) as a function of the residue number. Data corresponding to RRM1 and RRM2 are coloured in blue and green, respectively.Overlay of the 2D ^1^H‐^1^H TOCSY spectra of U1 snRNA SL3 before (black) and after (red) addition of SRSF1∆RS.Overlay of the 1D ^1^H SOFAST imino recorded upon successive addition of SRSF1∆RS. The spectra are coloured according to the SL3:SRSF1∆RS ratio.Suppression of a 5′SS mutant by a complementary U1 snRNA is reduced by mutations of the nucleotides in U1 SL3 predicted to be bound by SRSF1. The diagram shows base‐pairing between the 5′ end of U1 snRNA and SMN1 exon 7. (1) Wild‐type sequences, with the dot representing the 2,2,7‐trimethylguanosine cap at the 5′ end of U1 snRNA and *Ψ* representing pseudouridine; a bulged A is shown. (2) A mutated nucleotide in the SMN1 exon 7 minigene that impairs base‐pairing with U1 snRNA is shown in red. (3) A compensating mutation was made in the gene encoding U1 snRNA. (4) An additional mutation was made in the U1 snRNA gene to improve base‐pairing with the SMN1 exon 7 5′SS. (5) The U1 gene containing two mutations that allow efficient suppression of the mutated exon 7 5′SS was mutated further in SL3, as shown by the sequences of SL3 below, to alter those nucleotides that interact with SRSF1 (underlined in the SL3 wt sequence) without affecting the base‐pairing in SL3. Minigenes expressing the wild‐type and mutated sequences of SMN1 exon 7 and U1 snRNA were transfected into HEK293T cells, and the level of inclusion of SMN1 exon 7 was detected by RT–PCR. The products were separated by agarose gel electrophoresis and detected by ethidium bromide. The scatter plot shows the percentage of exon 7 inclusion for the 5 conditions. The data points are shown as well as the mean and the standard deviation. One‐way ANOVA test was used to probe the significance of the data, and *** indicates that *P* < 0.005 (*n* = 5 biological replicates) while n.s. stands for non‐significant.Structural model of the SRSF1∆RS‐SL3 complex.Heat map showing the distribution of ENCODE cross‐links of selected proteins along U1 snRNA. The scale bar on the right‐hand side shows the probability that the cross‐links are enriched at a given position. The sequence of U1 snRNA, showing the stem‐loops, is aligned on the l.h.s.

This interaction was further investigated in the context of the SRSF1∆RS‐SL3 complex, which has a smaller molecular weight and allows structural studies in solution. Using ^15^N‐labelled protein, we monitored the binding of SRSF1∆RS on the U1 snRNA SL3 (Fig 6A). SRSF1∆RS experienced amide CSP on both RNA‐binding domains, similar to the ones observed when the protein was titrated with ssRNA. The chemical shifts of the H5‐H6 of pyrimidines were also monitored upon addition of SRSF1 and CSP occurred mainly on the pyrimidines of the double‐stranded region (U95‐C100) and on the C101 from the loop (Fig 6B). In contrast, the binding of FUS RRM on SL3 induced CSP on U105 and U107 from the 3′ part of the loop (Jutzi *et al*, 2020). Altogether, the data suggest that RRM1 binds to the CA motif at the 5′ side of the loop and RRM2 to the GGA sequence in the 3′ side of the stem. Using isothermal titration calorimetry, we determined a dissociation constant of 10.9 ± 2.8 μM at 27°C that is roughly 200‐times weaker than the affinity of SRSF1∆RS for its ssRNA target (Appendix Fig S9; (Clery *et al*, 2021)). Since the GGA motif is embedded in the upper part of SL3 and RRM2 is a ssRNA binder, it seems likely that SL3 has to be melted in order to be bound by RRM2. To validate this hypothesis, we monitored the imino proton signals of SL3 at 40°C upon addition of SRSF1∆RS using a highly sensitive 1D ^1^H SOFAST based on an imino‐selective excitation pulse ((Nikolaev *et al*, 2019); Fig 6C). The data showed that several imino signals in the upper part of the stem including U97 and G109 strongly decreased in intensity upon addition of SRSF1∆RS, suggesting that binding of the protein on SL3 induces the melting of the upper part of SL3. The competition between the formation of the base pairs and the binding of RRM2 on the GGA could explain why SRSF1∆RS has a low affinity for SL3 *in vitro*.

### Functional relevance of the SRSF1‐U1 snRNP interaction

The functional importance of these two binding sites in stem‐loop 3 was tested using an assay in which the loss of splicing caused by 5′SS mutations was suppressed by expression of mutant U1 snRNAs carrying complementary nucleotide changes in their 5′ ends (Zhuang & Weiner, 1986; Roca *et al*, 2012). The 5′SS of *SMN1* exon 7 was mutated at position +4 (A_+4_U) to impair splicing mediated by the endogenous U1 snRNP (Fig 6D). To rescue splicing, a plasmid was co‐transfected that expressed U1snRNA Ψ_+5_A with the endogenous promoter. However, the splicing was only partially rescued (Fig 6D). Since the bulged adenine in position −1 strongly weakens the 5′SS of SMN exon 7 (Campagne *et al*, 2019), the compensating C_‐9_U mutation was introduced into the suppressor U1 snRNA, and the combination of both mutations rescued the splicing of SMN1 exon 7. Mutating both putative binding sites of SRSF1 on stem‐loop 3 resulted in a strong reduction of the activity of the suppressor U1 snRNA (Fig 6D), consistent with a functional role in splicing for these two SRSF1‐binding sites in U1 snRNP stem‐loop 3. Interestingly, the SL3 mutations strongly reduced the binding of SRSF1 *in vitro* while the binding of FUS remained unchanged (Appendix Fig S9). The importance of SL3 for splicing was shown previously in oocytes (Hamm *et al*, 1990), but we describe here the first evidence that it binds SRSF1, a central determinant of splicing activity, and propose a structural model for this interaction (Fig 6E).

### SRSF1 cross‐links preferentially with U1 stem‐loop 3 in cells

To confirm that SRSF1 interacts specifically with SL3 *in vivo*, we analysed the distribution of cross‐linking sites detected by eCLIP (Van Nostrand *et al*, 2020). To exclude the possibility that an apparent preference for SL3 might simply reflect non‐specific binding to the most accessible portions of the RNA, we compared this distribution with that of the other 146 RNA‐binding proteins (Van Nostrand *et al*, 2020). The heat map in Fig 6F shows that cross‐links to SRSF1 are concentrated in the loop of SL3 and to the 3′ side of the stem, in perfect agreement with our NMR study and our splicing assay, and very few cross‐links elsewhere. While other proteins show cross‐links to the loop of SL3, relatively few have the cross‐links on the 3′ side of the stem (Appendix Fig S10). The only other regulatory proteins known at present to bind U1 snRNA are PTBP1 and FUS. PTBP1 shows most cross‐links in the region of U1 SL4, its known binding site (Sharma *et al*, 2011), supporting the validity of this analysis. FUS binds SL3 at the UGU sequence in the 3′ part of the loop (Jutzi *et al*, 2020), in between the CA and GGA motifs implicated here for SRSF1, consistent with the eCLIP data. The significance of the prominent SL4 cross‐links is unknown but may originate from binding of the RS domain. The only proteins with cross‐link distributions similar to SRSF1 are SRSF7 and SRSF9, in the adjacent tracks in Appendix Fig S10. We conclude that SRSF1 interacts with SL3 *in vitro* and *in vivo* and that this may be at least part of the reason why SL3 is essential for splicing.

## Discussion

### A new model for SRSF1 recruitment to pre‐mRNA

**Figure 7:**
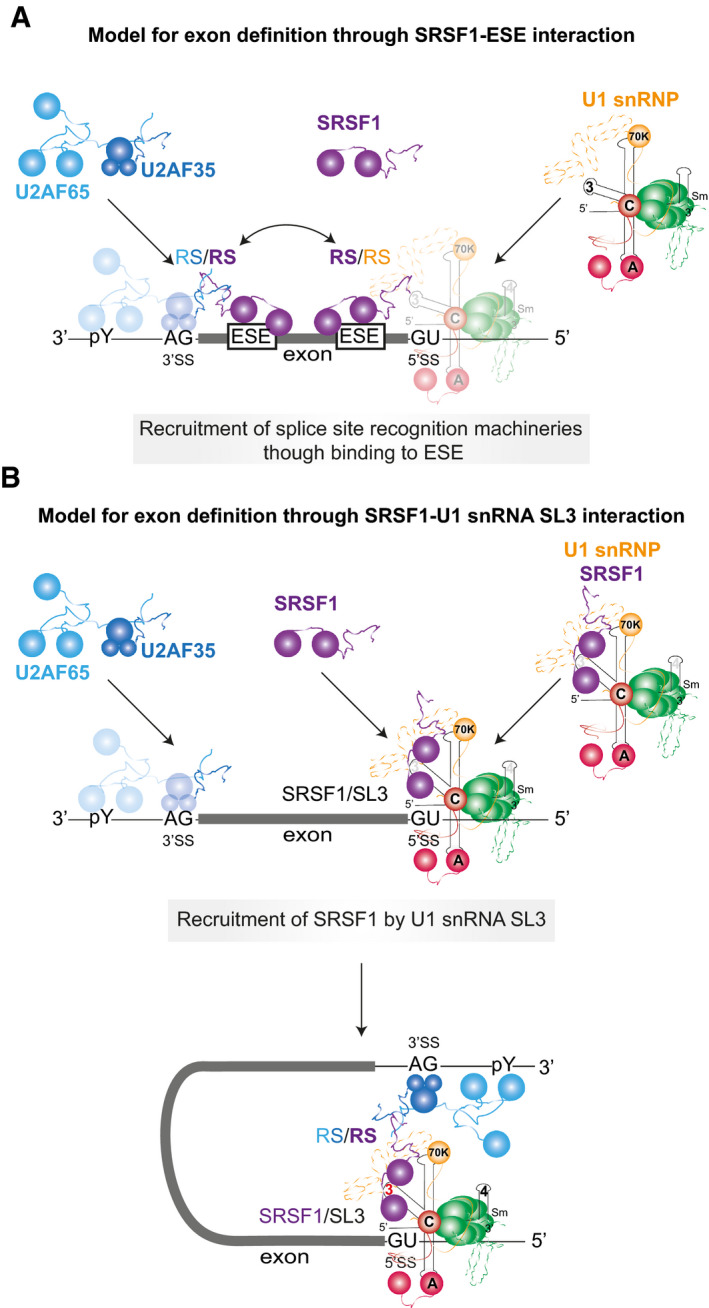
Mechanisms by which SRSF1 and U1 snRNPs collaborate in pre‐mRNA splicing The ESE‐dependent activity of SRSF1. SRSF1 probably binds transiently to the ESE and is in a dynamic equilibrium with the free protein until it collides by 3D diffusion with a U2AF/U2‐pre‐mRNA complex (Jobbins *et al*, 2018). Where binding of the 3′ splice site components would otherwise be weak, these interactions enhance the stability of binding of both SRSF1 and the 3′ complex. Similarly, the binding of SRSF1 to an ESE may enhance U1 snRNP recruitment.Recruitment of SRSF1 by interaction with U1 SL3 (this paper) may enable cross‐exon interactions (exon definition) and cross‐intron interactions. SRSF1 is not recruited to the pre‐mRNA by its interaction with an ESE but by its interaction with the U1 snRNA, which thereby brings SRSF1 to the 5′ splice site. This recruitment of SRSF1 may enable it to enhance the use of an upstream 3′SS in the same way as it does when bound to an ESE. SRSF1 recruited by a U1 snRNP may also mediate cross‐intron interactions with the same partners but at the downstream 3′SS during splicing reactions (Wu & Maniatis, 1993). In addition, in both cross‐intron and cross‐exon configurations, U1 snRNP‐bound SRSF1 may recruit the tri‐snRNP to the intron definition or exon definition A complexes (Roscigno & Garcia‐Blanco, 1995; Schneider *et al*, 2010). Competition by other proteins for SL3 might be the basis for many examples of regulation.

The conventional model for the actions of SRSF1 on pre‐mRNA splicing involves binding of the SR protein to exonic splicing enhancer sequences and the recruitment of U1 snRNP at the 5′ SS and U2‐associated factors at the 3′ SS (Fig 7A). This is consistent with extensive data from transcriptome‐wide analyses of binding sites by cross‐linking, which have revealed that these are enriched upstream of 5′ splice sites and, in some cases much more strongly, at the 5′ end of exons just downstream of the 3′ splice sites (Jamison *et al*, 1995; Sanford *et al*, 2009; Pandit *et al*, 2013; Anczukow *et al*, 2015; Bradley *et al*, 2015; Muller‐McNicoll *et al*, 2016). The results we describe here show that there is another and completely different mode by which SRSF1 associates with pre‐mRNA splicing complexes.

By using single‐molecule methods that distinguish between complexes containing different numbers of molecules of SRSF1, we have been able to show that SRSF1 can associate in a defined stoichiometry with pre‐mRNA in a process that is independent of exons but dependent on U1 snRNPs and 5′SSs. This occurs whether the 5′SS is able to participate in splicing or is at the 3′ end of an exon and participates in exon definition (Fig 7B). Even in the absence of pre‐mRNA, significant proportions of SRSF1 molecules and U1 snRNPs are associated in a stoichiometric complex, from which we infer that the binding of U1 snRNP to a 5′SS concomitantly recruits SRSF1. Our results are consistent with previous evidence showing that pure SRSF1 associates directly with U1 snRNPs (Kohtz *et al*, 1994; Xiao & Manley, 1997; Cho *et al*, 2011), forming stable complexes in the presence of a 5′SS (Jamison *et al*, 1995) and, moreover, evidence from immunoprecipitation that U1 snRNPs and SRSF1 are associated *in vivo* (Ellis *et al*, 2008; Chi *et al*, 2018; Huttlin *et al*, 2021), independently of RNA (Ellis *et al*, 2008). Further support for the existence of this interaction *in vivo* comes from FRET, which showed that the U1 70K protein is in close proximity to SRSF1, even in the absence of transcription (Ellis *et al*, 2008).

### Implications of SRSF1 binding to U1 snRNA SL3

The interaction of U1 snRNPs with SRSF1 has previously been attributed to protein–protein interactions between either the RS domain of U1‐70K, the snRNP protein that binds stem‐loop 1 of the U1 snRNA, and the phosphorylated RS domain of SRSF1 (Xiao & Manley, 1997) or between RRM domains of the same proteins (Cho *et al*, 2011). The interaction with SL3 was unexpected, although we have also shown recently that SL3 is bound by the FUS protein (Jutzi *et al*, 2020). Interestingly, SL3 is much longer in yeast U1 snRNPs and it has been proposed to act as a binding site for regulatory proteins (Li *et al*, 2017). The interaction of SRSF1 with bases in the stem of SL3 is not unprecedented, since the regulatory protein PTBP1 interacts with bases in the stem of U1 snRNA stem‐loop 4 (Sharma *et al*, 2011). Given that the RNA‐binding domains of SRSF1 interact only weakly with ESE sequences (Cho *et al*, 2011; Anczukow *et al*, 2015; Jobbins *et al*, 2018), it seems likely that the SL3 interaction is augmented by protein interactions. These probably involve the RS domain of SRSF1, since no protein interactions were detected by NMR spectroscopy with the SRSF1∆RS protein (Fig 4A). In addition, the unwinding seen in the upper part of the stem suggests the possibility that a helicase is involved. Candidates for this activity might be UAP56 (DHX39B), which is present in the earliest complexes (Fleckner *et al*, 1997; Shen *et al*, 2008) and has also been found to interact with SL3 (Martelly *et al*, 2021), or DDX5, which is involved in the use of 5′SS and U1 snRNP binding or dissociation (Liu, 2002; Guil *et al*, 2003; Lin *et al*, 2005a; Kar *et al*, 2011). SRSF7 and SRSF9 show similar cross‐linking distributions on U1 snRNA, which suggests that they may also interact with SL3 (Appendix Fig S10). Other proteins interact with SL3, but most of them do not interact with the 3′ side of the stem and therefore may not compete with SRSF1. However, there are several that do seem to have the potential capability. It should be noted, though, that SRSF1 is present at a several‐fold higher concentration than any potential competitors in HeLa cells (Hein *et al*, 2015). This does not mean that all functional competitors also compete for SL3; hnRNP A1, for example, is a functional competitor with SRSF1 (Mayeda & Krainer, 1992; Mayeda *et al*, 1993; Caceres *et al*, 1994; Eperon *et al*, 2000; Zhu *et al*, 2001) but it does not interact with the U1 snRNP (Eperon *et al*, 2000) and accordingly does not show specific binding on U1 snRNA. It seems probable that U1 snRNA is a target for a number of RNA‐binding proteins and that their competition would be a major determinant of splicing patterns and efficiency in mammalian cells.

### Alternative modes of SRSF1 association explain the functional equivalence of ESEs and 5′SS in exon definition

The SRSF1 molecule recruited by U1 snRNPs might be expected to have roles in either definition of the upstream exon or in splicing of the downstream intron. The effects on exon definition of a downstream 5′SS on short internal exons (Hwang & Cohen, 1996a; Fox‐Walsh *et al*, 2005; Erkelenz *et al*, 2021) are similar to those of an ESE (see above). Indeed, there is a negative correlation between the strength of 5′SS sequences and the density of ESEs in the flanking exon sequences (Kralovicova & Vorechovsky, 2007; Caceres & Hurst, 2013). Previously, the combination of ESEs and splice site strength would have been interpreted as a means to ensure a minimum probability of U1 snRNP binding; in the light of our finding that the U1 snRNP recruits SRSF1, we might speculate instead that ESEs and U1 snRNP binding are also alternative ways to recruit SRSF1 for exon definition. This possibility is strengthened by the absence of any evidence for direct contacts by U1 snRNP in cross‐exon interactions in mammals. While U1 snRNP does produce an increase in U2AF65 binding to the upstream 3′SS (Grabowski *et al*, 1991; Kreivi *et al*, 1991; Hoffman & Grabowski, 1992; Cote *et al*, 1995; Izquierdo *et al*, 2005; Singh *et al*, 2007; Palacino *et al*, 2015), the intermediate interactions across the exon have not been identified. Our findings raise the possibility that these interactions are mediated by U1‐associated SRSF1 that interacts directly with U2AF (Wu & Maniatis, 1993) or U2 snRNP (Akerman *et al*, 2015), exactly as would an ESE‐bound SRSF1 molecule (Fig 6). Direct interactions are also consistent with our finding that only one SRSF1 is recruited in complex A and there is no evidence of cooperative association of additional molecules of SRSF1 across the exon. Wu and Maniatis proposed such a bridging role for SRSF1 in cross‐intron interactions (Wu & Maniatis, 1993), but ESE‐based models have predominated in discussions of exon definition.

### The uncertain role of U1 snRNP in splicing a downstream intron

A fundamental structural similarity between exon‐bridging and intron‐bridging splicing complexes has been proposed in yeast, where Prp40 contacts the U1 snRNP and the branchpoint‐binding protein (Abovich & Rosbash, 1997; Li *et al*, 2019). Whether, by analogy, the recruitment of SRSF1 by U1 snRNPs in mammals is important for splicing of the downstream intron is unclear. Surprisingly, it is not yet known whether the U1 snRNP itself has any direct role. The U1 snRNP was the first splicing factor discovered, and it has well‐characterized roles in the selection of 5′ splice sites (Roca *et al*, 2013). Its status as a splicing reaction component was originally in doubt because of its weak association with splicing complexes (Konarska & Sharp, 1986), but it is now seen as a stoichiometric component (Will & Luhrmann, 2011; Roca *et al*, 2013) until it is displaced during the formation of complex B (Charenton *et al*, 2019). It has been suggested that it forms cross‐intron interactions via association with the proteins SF3a (Sharma *et al*, 2014), Prp5 (Xu *et al*, 2004; Shao *et al*, 2012) or Prp40 (Abovich & Rosbash, 1997), although none of these have been shown to bind as a single molecule and span the intron. The possibility that the U1 snRNP plays a direct role in the splicing reactions is weakened by three observations. First, it has been shown to enable splicing even when binding some nucleotides away from the 5′ splice site (Hwang & Cohen, 1996b; Brackenridge *et al*, 2003). The exact position of the 5′ splice site is determined by U6 snRNA base‐pairing (Hwang & Cohen, 1996b; Brackenridge *et al*, 2003; Hang *et al*, 2015). Second, the U4/U6.U5 tri‐snRNP can bind directly to a 5′ splice site, independently of U1 snRNP and even in competition with it (Konforti *et al*, 1993; Konforti & Konarska, 1994; Maroney *et al*, 2000). Interestingly, the recruitment of the tri‐snRNP is enhanced by SR proteins (Roscigno & Garcia‐Blanco, 1995). Third, the splicing of some introns has been found to be independent of the U1 snRNP (Crispino *et al*, 1996; Fukumura *et al*, 2009; Raponi *et al*, 2009). Remarkably, some introns that are U1‐dependent have been shown *in vitro* to splice without U1 snRNP if the concentration of SR proteins is increased (Crispino *et al*, 1994; Tarn & Steitz, 1994). It can be inferred that the U1 snRNP might not play a direct role in all splicing reactions but could also act upstream of SRSF1 in the reaction pathway.

### SRSF1, recruited by U1 snRNP, might play similar roles in exon definition and splicing of the downstream intron

Evidence for a direct role of SRSF1 in splicing is limited, but not inconsequential. As noted above, SR proteins can substitute for U1 snRNP or recruit the tri‐snRNP, and the catalytic reactions of splicing require dephosphorylation of SRSF1. When SRSF1 was added to an S100 cytoplasmic extract to restore splicing, it was found that its RS domain contacted the 5′SS in complexes B and C and that it was required for U6 snRNA base‐pairing to the 5′SS (Shen & Green, 2004). The addition of SRSF1 was not required either when an RS domain was tethered by MS2 near to the 5′SS (Shen & Green, 2004) or when the base‐pairing between U6 snRNA and the 5′SS had been improved (Shen & Green, 2007). Dephosphorylation of SRSF1 is required after assembly of complex B (Cao *et al*, 1997) which, like the cross‐linking results (Shen & Green, 2004), is consistent with the presence of SRF1 in the mature spliceosome. Most strikingly, SRSF1 RRM2 is visible as a discrete component and structure in pre‐B^act^ spliceosomal complexes, which form after the U1 snRNP has been displaced (Townsend *et al*, 2020). These observations suggest that SRSF1, recruited by a U1 snRNP to the 5′SS, which we demonstrate here, might play similar roles in constitutive splicing reactions and exon definition. First, interactions with U2AF would establish connections across the intron or exon, respectively. In addition, SRSF1 might in both cases recruit the tri‐snRNP. This would explain its persistence in splicing complexes B or even C, after the departure of U1 snRNPs. Moreover, exon definition complexes also contain tri‐snRNPs and can assemble complex B directly (Schneider *et al*, 2010). If binding to SL3 and SL4 is the focus of competition among splicing regulatory proteins, then it is possible that much regulation occurs at the point of tri‐snRNP recruitment. Our finding that SRSF1 binds U1 snRNA SL3 and is recruited by U1 snRNPs supports the possibility that SRSF1 is playing much wider roles in pre‐mRNA splicing than just the binding of exonic splicing enhancers.

## Materials and Methods

### Sequences

Most of the transcripts used were derivatives of rabbit β‐globin exon 2‐exon 3, with a shorter intron and truncated 3′ exon, as described previously (Skordis *et al*, 2003; Hodson *et al*, 2012). The exons in the pre‐mRNA with 2 nts in each exon comprised (5′) GG and (3′) GA. The globin‐SMN2 exon 7 transcripts were also as described (Skordis *et al*, 2003; Smith *et al*, 2014), but transcription terminated at nucleotide 48 in SMN2 exon 7 so as to exclude the exonic part of the 5′ splice site.

### Oligonucleotide annealing

The transcription of RNA substrates and the subsequent annealing of the fluorescent oligonucleotide βg‐5′‐Cy5 (Cy5‐UAGACAACCAGCAGCC C‐biotin, 2′‐*O*‐methyl/LNA) were done as described (Hodson *et al*, 2012). All labelled constructs were run on a 6% polyacrylamide gel and imaged using a Typhoon imager (GE Healthcare) to ensure that the level of free fluorescent oligonucleotides was below 2%.

### Labelling RNA at the 5′ end

For Figs 2C and 3, the substrate RNAs were labelled using 5′ Cy5‐maleimide (Ohrt *et al*, 2012). RNA was transcribed in the presence of 10 mM guanosine‐5′‐*O*‐monophosphorothioate (BioLog) instead of cap analogue. Following transcription, the mixture was incubated with RNase‐free DNase and extracted thrice with phenol–chloroform. After purification of the RNA by gel filtration on S‐300 (GE Healthcare), the RNA was precipitated with ethanol and incubated for 4 h with 0.5 mM cyanine5‐maleimide (Lumiprobe 13080) at ambient temperature. The RNA was purified by high resolution denaturing polyacrylamide gel electrophoresis.

### Splicing and analysis of complexes

Following transfection of HeLa cells with plasmids expressing mEGFP‐SRSF1 (with or without the addition of mCherry‐U1A), the cells were cultured for about 48 h and extracts were prepared. Aliquots of the extracts were made and stored at −80°C. The activities of the extracts in splicing and complex assembly were analysed as described previously (Hodson *et al*, 2012). Proteins were detected by Western blotting, using primary antibodies against GFP (ProteinTech 66002) or SRSF1. Fluorescent secondary antibodies were used against mouse IgG (IRDye 680LT, LI‐COR 926‐68020) and rabbit IgG (IRDye 800CW, LI‐COR 926‐32211). The concentration standard was recombinant GFP. The ratios of labelled and endogenous proteins in the various extracts are shown in Table 1. The total concentrations of SRSF1 (labelled and endogenous) in NE1 and NE2 are around 1.7 and 1.2 µM, respectively, and the respective concentrations in the splicing reactions would be 0.8 and 0.6 µM. This is comparable with a previous estimate of 1.3 µM for the concentration of SRSF1 in a splicing reaction *in vitro* (Mayeda *et al*, 1993).

### Sample preparation

For reactions containing pre‐mRNA labelled with biotinylated oligonucleotides, coverslips (22 mm by 50 mm #1, Menzel‐Gläser) were washed with distilled water, followed by purified water and sonicated in a water bath for 12 min and dried under a nitrogen stream. They were cleaned in an argon plasma (MiniFlecto‐PC‐MFC, Gala Instruments) five times for 5 min each with pure argon at 0.15 mbar and an applied power of 80 W. Double‐sided tape was used to create a channel of width 5–10 mm parallel to the coverslide, which was covered by another coverslip, 21 mm by 26 mm #1, to form the sample chamber. The sample chamber was incubated with 20 μg/ml biotin‐BSA in PBS for 10 min, washed with buffer A (100 mM NaCl, 50 mM HEPES pH 7.5, 1 μM DTT, 20 units/ml RNase OUT [Invitrogen]), incubated with 10 μg/ml streptavidin (Invitrogen) in PBS and again washed with buffer A.

All samples were prepared with 50% nuclear extract, 3.2 mM MgCl_2_, 50 mM monopotassium glutamate and 3 units RNase OUT (Invitrogen) for incubations without ATP, and for incubations in the presence of ATP 20 mM phosphocreatine, 1.5 mM ATP and 20 mM HEPES pH 7.5 were included. To halt splicing at complex A, either 1 μM of a 2′‐*O*‐methyl oligonucleotide complementary to U6 or 570 nM anacardic acid was added, as described (Hodson *et al*, 2012). U1 snRNA was inhibited by the inclusion of a complementary 2′‐O‐methyl oligonucleotide (GCCAGGUAAGUAU–biotin) at 3.3 μM. PhosSTOP (Roche) was included as a broad‐spectrum phosphatase inhibitor at a concentration of 2×. Samples were pre‐incubated for 15 min at 30°C before pre‐mRNA was added at a final concentration of 50–60 nM and incubated for a further 15 min at 30°C. Samples were diluted using buffer A, loaded into the sample chamber and incubated for 5 min. The sample chamber was then flushed with 2mM protocatechuic acid (Spectrum Chemicals), 90 nM protocatechuate 3,4‐dioxygenase (Sigma), 1mM DTT, 0.16 units/μl RNaseOut and incubated for a further 5 min.

For reactions containing 5′ end‐labelled RNA, coverslips were soaked in 1 M KOH for four hours before washing in distilled water, sonication and plasma cleaning as above. Biotinylated BSA and streptavidin were omitted. Buffer A was replaced with 40 mM HEPES pH 7.5, 3.2 mM MgCl_2_, 50 mM monopotassium glutamate, 50 mM KCl, 0.1 mM EDTA and 0.5 mM DTT. Complexes were adsorbed directly onto the glass surface. Although the adsorbed complexes are stable, transient interactions might be lost during dilution and the period of a few minutes during which complexes diffuse to the slide surface after loading. Experiments to test whether the interaction between mEGFP‐SRSF1 and mCherry‐U1A was dependent on pre‐mRNA were done by including 1 µl of an RNase A/T1 mixture (Thermo Scientific; RNase A at 2 mg/ml and RNase T1 at 5 units/µl) in a 10 µl reaction that otherwise reproduced splicing reaction conditions.

### Data acquisition

An objective‐based total internal reflection fluorescence microscope was used for all single‐molecule experiments. The laser power incident on the objective was approximately 4 mW at 488 nm and 3 mW at 561 nm. Only the central part of the approximately Gaussian‐shaped intensity profile of the laser beam was used, together with the central 250 by 250 region of the 512 by 512 pixels CCD chip to ensure a homogenous illumination across the field of view (≤ 50%). The internally reflected beam was directed onto a quadrant photodiode where its displacement was measured. It was fed back to the microscopic stage to create a feedback loop to ensure the positional stability of the sample in the nanometre range. The acquired data were saved in the Tagged Image File Format (TIFF) as well as an accompanying text file containing all the information about the execution of the individual acquisition. A typical acquisition involved the successive collection of 50 frames with excitation at 633 nm, to identify Cy5‐pre‐mRNA spots, followed by 250 frames at 561 nm (mCherry, where collected) and 250 frames at 488 nm (mEGFP). Frames were collected at a rate of 20 s^−1^. All fluorophores bleached completely during the appropriate stage. Between 50 and 100 fields were acquired from each channel on the coverslip.

### Data analysis

The data were analysed with a MATLAB program. Spots were detected by creating two composite images for each wavelength by computing the mean and the maximum value for each pixel across all images of a wavelength. Spots were identified by moving a square, 19 pixels by 19 pixels, across the composite images to find areas where the maximum pixel intensity inside the box was 1.119 times higher than the mean pixel intensity. The size of the box was reduced until only one peak of intensity was within this box, and rejected if the size was ultimately smaller than 9 pixels by 9 pixels. The intensity around the remaining peak was fitted with a 1D Gaussian for each dimension, with 0× at the centre:

$$G\left(x\right)=B+\;\frac{1}{\sqrt{2\pi }}\;\times \exp\left[‐\frac{{\left(x‐\mu \right)}^{2}}{{2\sigma }^{2}}\right].$$

A spot was considered valid, if the fit yielded values for 0.9 ≤ σ ≤ 3.5, 0 ≤ *B* ≤ 0.4 for Cy5, and 0.3 ≤ *σ* ≤ 4.7, 0 ≤ *B* ≤ 0.53 for mEGFP and if |µ| < size of the box – 2.5. For each dimension, µ was taken as the true centre of the spot. A linear transformation in each dimension to correct for chromatic aberration yielded the “true” position of each spot (*x*_t_, *y*_t_):

$$x_{t}=x+\frac{\left(x‐x_{c}\right)}{{\mathrm{SF}}_{x}},\;y_{t}=y+\frac{\left(y‐y_{c}\right)}{{\mathrm{SF}}_{y}}.$$

The values for the parameters *x*_c_, SF_x_, *y*_c_ and SF_y_ were calibrated using oligonucleotides labelled with both ATTO 488 and Cy5. The coordinates of the molecular spots were measured with excitation at 488 and 633 nm, and then, the data were fitted by a model in which divergence of the 488 nm signal from that at 633 nm is proportional to distance from a centre at which no divergence occurs. This produces the coordinates of the centre (*x*_c_, *y*_c_) and the scaling factors (SF_x_, SF_y_).

Two spots of different wavelengths were considered colocalized if their corrected separation was below or equal to two pixels. The probability of random background colocalization was calculated for each image and was generally around 1–2%. This was considered to be so small that it was not subtracted from the colocalization values.

Background‐corrected intensity traces for each spot were calculated from the raw data by subtracting the mean intensity of all the pixels that were five pixels away from the centre from the mean intensity of the pixels found in a central box of three pixels by three pixels. Step detection was done recursively by a Bayesian step detector (Ó Ruanaidh & Fitzgerald, 1996) to identify the most likely change points for the original trace and each following sub‐trace to the left and right of the identified change‐point, until the likelihood of the change‐point is below a set threshold. These change points were used to estimate the intensity plateaus of the trace, thus allowing any re‐activation of fluorophores to be discounted. The change points were checked by visual inspection and assignments accepted or corrected. The number of plateaus detected minus one was taken as the number of fluorophores present. The validity of the assignment of the step numbers to the traces was checked for an experiment in which a substantial number of RNA molecules were deemed to be associated with two molecules of mEGFP‐SRSF1 (Fig 4B, GloC‐U1) by measuring the total number of photon counts emitted by mEGFP for each complex, and comparing the results from complexes assigned as containing one or two molecules of mEGFP‐SRSF1 (Appendix Fig S11). The median number of photon counts emitted from complexes containing one molecule of mEGFP‐SRSF1 was 1,472, whereas the median for complexes containing two was 3,085. The distribution between the two classes was significantly different by a two‐tailed Mann–Whitney test (SPSS); *P_1 = 2_* = < 0.001. As a check, the values from the complexes containing one molecule were added to the same values rearranged in a random order. The pseudo‐double complexes had a median of 3,802, and the distribution was not significantly different from that of the *bona fide* complexes assigned as having two molecules (*P_2 = (1 + 1)_* = 0.2).

### Statistics

Each frequency histogram shows the result of measurements collated from around 50 fields acquired from each sample. The error bars are the square root of the variance of the binomial probability that an RNA spot will be associated with the given number of protein bleaching steps. The expected distributions for binding by a single molecule were taken to be the same as those seen for bleaching in one or two steps under the same conditions but in the absence of pre‐mRNA (Table 1). The distributions expected if there were binding by two molecules on pre‐mRNA with duplicated splice sites were calculated based on the observed levels of dimerization in the absence of pre‐mRNA and the proportions of protein labelled, as described previously (Hodson *et al*, 2012; Chen *et al*, 2017). Chi‐squared tests included frequency classes with expected values ≥ 5. The results were significantly different from the expected distributions. This could result from either pre‐mRNA associated with only one or no molecules of SRSF1 or the existence of non‐fluorescent (misfolded or pre‐bleached) mEGFP. To avoid over‐estimating binding, it was assumed that all molecules were fluorescent (previously, with an EGFP‐PTB assumed to occupy all available pre‐mRNA fully we estimated that > 90% were active) and the probability of occupancy and the total number of accessible molecules of pre‐mRNA were allowed to vary. This enabled an estimation of the fraction of pre‐mRNA molecules bound by SRSF1 that contained two molecules of SRSF1 by minimization of chi‐squared. However, the extra variables precluded the use of chi‐squared tests of significance. An additional complication was the existence of the significant background from molecules that did not form complex A. Poisson and geometric distributions were fitted to this background data by minimizing the value of chi‐squared, and tests of significance were done on those categories in which 4 or more pre‐mRNA molecules were expected using N‐2 and N‐3 (respectively) degrees of freedom.

With a pre‐mRNA containing two introns (Fig 1B), we estimated that about as many SRSF1‐pre‐mRNA complexes contained two molecules of SRSF1 as contained one. This was roughly consistent with our previous measurements of U1 snRNP binding to the same pre‐mRNA. This calculation was based on the proportion of SRSF1 protein that was tagged by mEGFP and on the observed pattern of dimerization in the absence of exogenous RNA. This procedure might underestimate the level of complexes containing two molecules of SRSF1, since a proportion of mEGFP molecules might not be actively fluorescent. Inactive mEGFP might result from several factors: (i) a significant proportion of mEGFP molecules might have entered a brief dark state, known as blinking; (ii) bleaching might have occurred before fluorescence was recorded; and (iii) a significant proportion of mEGFP molecules might be misfolded. Blinking (i) would not affect the results, since the dark state lifetime of mEGFP is only 1–2 s (Dickson *et al*, 1997; Garcia‐Parajo *et al*, 2000; Vamosi *et al*, 2016). Spots were detected in our experiments using both the mean and maximum fluorescence, meaning that the total yield from a fluorophore is unimportant, and measurements were made for 15–30 s, so that the probability of being in a dark state throughout is negligible. The second factor, bleaching prior to recording or so early in recording that no mean level of initial fluorescence was measured, undoubtedly happens but is likely to affect a relatively small proportion of molecules because fluorescence was recorded continuously during the switch from illumination at 633 nm (for Cy5 excitation) to 488 nm (for mEGFP). The third factor, misfolding, is harder to estimate. It is usually estimated, when the proteins cannot be purified, by assuming that the population of complexes is homogeneous. In such a case, the distribution is analysed as we have done here and the difference from the expected distribution is attributed to misfolding. It would not be justified in the present work because there are no grounds for the *a priori* assumption of homogeneity; indeed, the question at issue is whether more than one molecule of SRSF1 is bound. Thus, we considered it safer to assume that misfolding was negligible and that the population was heterogeneous. In the first assay of this type, misfolding was estimated at 20% (Ulbrich & Isacoff, 2007), although in this case the EGFP sequence was expressed on the C‐terminal side of the fusion. Misfolding of mEGFP is particularly likely in this location, since folding would be affected by any misfolding of the upstream sequence (Waldo *et al*, 1999; Wang & Chong, 2003). In contrast, it has been reported that the maturation of N‐terminal GFP fusions expressed in HEK293 cells was almost complete (Liesche *et al*, 2015). The fluorescent protein moieties in mEGFP‐SRSF1 and mCherry‐U1A are N‐terminal and expressed in mammalian cells. The absence of significant levels of dark mEGFP is consistent with the results in Fig 4. In Fig 4A, the results for BGSMN2‐ESE are consistent with full binding by two molecules of SRSF1 (c.f. Appendix Fig S2), and in Fig 4B, the results suggest that double occupancy of 92% of GloC‐U1 pre‐mRNA molecules by U1 snRNPs is associated with double occupancy of 67% by SRSF1. Our estimate that about half of the complexes of SRSF1 with pre‐mRNA containing two introns contained two molecules of SRSF1 in Fig 2A is therefore a minimum but probably close to the correct value.

An alternative explanation for the binding of two molecules of mEGFP‐SRSF1 might be that pre‐mRNA substrates with ESEs or an additional U1 snRNP‐binding site might be more prone to dimerization. Analysis of the data for GloC‐U1 in Fig 3B showed that about 10% of the Cy5 spots contained two molecules of pre‐mRNA. If these were excluded from the analysis, the results were the same (by a chi‐squared test, *P_inc = exc_* = 0.91), and the possibility was not taken into account further.

A less distinct form of binding produces a significant background of molecules bound by more than one or two molecules of SRSF1. This was conspicuous when U1 snRNA binding was blocked (Figs 2B and 4A). In some cases, the residual distribution was geometric, suggesting that it might be accounted for by cooperative interactions, but in other cases, the pattern was less clear. There are several reasons for thinking that this background was present to some extent in many of the experiments and represented a separate class of molecules from those bound primarily by a single SRSF1 molecule. First, if the U1‐dependent association involved binding of an additional molecule of SRSF1 to RNA molecules already bound by SRSF1 according to the background distribution, then the net effects would be a shift of the distributions from *n* to *n* + 1. On the contrary, the U1‐dependent increase is restricted to the complexes with one or two bleaching steps (according to the number of sites or introns). Second, we have shown (Jobbins *et al*, 2018) that additional copies of the ESE on the 3′ end of BGSMN2 (c.f. Fig 4A) increased splicing, increased complex A formation and reduced the background distribution. Third, U2AF35, U2AF65 and U2 snRNPs show a similar super‐stoichiometric distribution after sequestration of U1 snRNPs or ablation of 3′SS signals (Chen *et al*, 2017). Thus, the distributions that we observe probably result from the superimposition of distributions arising from two mutually exclusive processes: specific recruitment of SRSF1 molecules via U1 snRNPs or ESEs, and the non‐stoichiometric background complexes representing molecules unable to form complex A.

### Activity of SRSF1 fusions in alternative splicing

Plasmids expressing mEGFP‐SRSF1, mCherry‐SRSF1 or just the fluorescent proteins were transfected into HeLa cells. RNA was extracted after 48 h and analysed by reverse transcription and PCR. The PCR primers were complementary to sequences on either side of the SRSF1‐responsive intron 4 of the endogenous SRSF1 gene (Sun *et al*, 2010), and the products were 1,136 or 216 bp for intron‐retained or spliced isoforms, respectively. After ethidium staining, the intensities of the bands were measured using a Typhoon imager.

### Western blotting

GFP and U1A were detected using primary antibodies from ProteinTech (66002) and Abcam (ab55751), respectively; SRSF1 was detected using a monoclonal antibody kindly provided by Dr A.R. Krainer (Cold Spring Harbor Laboratory) (Hanamura *et al*, 1998). Fluorescent secondary antibodies were used against mouse IgG (IRDye 680LT, LI‐COR 926‐68020) and rabbit IgG (IRDye 800CW, LI‐COR 926‐32211). The recombinant GFP was kindly provided by Prof. C.R. Bagshaw (Leicester). For phosphatase treatment, nuclear extracts were incubated with shrimp alkaline phosphatase (NEB) at 1 unit/µl for 30 min at 37°C prior to gel electrophoresis.

### Analysis of eCLIP data from ENCODE

The alignment of reads to the snRNAs was based on the eCLIP‐seq Processing Pipeline v2.2 used in the ENCODE project (Consortium, 2012; Davis *et al*, 2018). FASTA files were downloaded from the ENCODE database for all eCLIP experiments performed in the K562 and HepG2 cell lines. As these files were already demultiplexed, they were first processed using the Cutadapt steps provided in the pipeline (Martin, 2011). Alignment was performed using Bowtie2, with output for unaligned reads, and discordant or mixed pairs discarded, against snRNA sequences obtained from Ensembl (Langmead & Salzberg, 2012; Yates *et al*, 2020). The output was then sorted and PCR duplicate removal performed using UMI‐tools (Smith *et al*, 2017). The resultant bam file was then processed using BEDTools bamToBed in paired‐end mode, with the output processed using awk so that the span of the whole pair was reported in standard bed format (Quinlan & Hall, 2010). This was then mapped using Bedmap to provide read counts at each position on the snRNAs in BED format (Neph *et al*, 2012).

Additional processing was performed using a custom Perl script to construct a BED file recording the 3ʹ terminus of each read pair. As the eCLIP was performed without a poly‐A pulldown, there was a substantial amount of noise in the percentage of total reads aligning to the snRNAs. To compensate for this, read counts were normalized at each position between the eCLIP experiments by taking the percentage of all pairs aligned to the specific snRNAs that were present at that position. For each experiment, the percentage at each position was then divided by the size‐matched control, providing a value for enrichment at that position compared to the control. Processing was parallelized using GNU parallel (Tange, 2018).

The logarithm of the enrichment value was subtracted from the logarithm of the percentage of reads terminating at each position. The averages and standard deviations were calculated for all experiments at each position and for each experiment across all positions. As the values were normally distributed both position‐wise and experiment‐wise, the cumulative probabilities for the position‐wise and experiment‐wise distributions were multiplied and the square root taken. Following this, the data were collapsed for each protein across cell types, by taking the product of all the probabilities at each position and then raising the result to the power of either 0.5 or 0.25, depending on whether there were 2 or 4 experiments for that protein, respectively.

### NMR spectroscopy analysis of the binding of SRSF1ΔRS to U1 snRNP

SRSF1ΔRS was produced in bacteria and purified as previously described (Anczukow *et al*, 2015). To ensure uniform ^15^N‐labelling and partial deuteration, the protein was produced in M9 medium prepared with fresh D_2_O and complemented with ^15^N‐labelled ammonium chloride (1 g/l) and protonated glucose (2 g/l). One hour before induction of protein expression, 100 mg/l of alpha‐ketobutyric acid (methyl‐^13^C, 99%; 3,3‐D2, 98%, Cambridge Isotope Laboratory) and 60 mg/l of alpha‐ketoisovaleric acid (^13^C5, 98%; 3‐D1, 98%, Cambridge Isotope Laboratory) were added to the medium to ensure specific ^13^C‐labelling of ILV methyl groups.

The reconstitution of U1 snRNP *in vitro* was performed as previously described (Campagne *et al*, 2019). The stem‐loop 3 RNA and the mutant were produced by T7 run off using double‐stranded DNA template (for SL3 wild type: 5′‐GGCCAGTGAATTCTAATACGACTCACTATAGCGATTTCCCCAAATGTGGGAAACTCGC‐3′; 5′‐GCGAGTTTCCCACATTTGGGGAAATCGCTATAGTGAGTCGTATTAGAATTCACTGGCC‐3′ and for SL3 mutant: 5′‐GGCCAGTGAATTCTAATACGACTCACTATAGCGATTTAAAAAAATGTGGGAAACTCGC‐3′; 5**′**‐GCGAGTTTCCCACATTTTTTTAAATCGCTATAGTGAGTCGTATTAGAATTCACTGGCC‐3′). After 4 h of incubation at 37°C, the transcription mixture was loaded into an HPLC system and the RNA was purified on an anion exchange column in denaturing conditions (85°C, 8 M urea). The RNA was extracted using butanol and refolded in water.

The binding of SRSF1ΔRS on U1 snRNP or U1 snRNA stem‐loop 3 was monitored by NMR spectroscopy in the NMR buffer (NaPO_4_ 10 mM pH7.0, l‐arginine 50 mM, l‐glutamate 50 mM, DTT 5 mM). Upon addition of U1 snRNP or U1 snRNA stem‐loop 3, the chemical shifts of the ILV methyl and amide groups were monitored on a 2D ^13^C‐^1^H HMQC and 2D 15N‐1H HSQC, respectively. The NMR data were recorded on a Bruker AVIII 700 Mhz at 313 K. Data were analysed using CARA (Keller, 2004).

The structural model presented in Fig 6E was generated using PyMol (Delano) and three following structures: the solution structure of FUS‐SL3 (PDB ID 6SNJ), the solution structure of SRSF1 ψRRM bound to 5′‐UGGAGGAC‐3′ (PDB ID 6M8D) and the solution structure of SRSF1 RRM1 bound to 5′‐AACAAA‐3′ (PDB ID 6HPJ). Briefly, the lowest energy structures of the SRSF1 RRMs bound to CA and GGA motifs were aligned on the stem‐loop 3 lowest energy structure from the FUS‐SL3 ensemble. FUS RRM was hidden as well as the original CA and GGA motifs of SL3. The resulting image is presented in Fig 6E.

FUS RRM was produced as previously described (Loughlin *et al*, 2019) and titrated with SL3 wild‐type and SL3 mutant at 30°C.

### Isothermal titration calorimetry

ITC experiments were performed on a VP‐ITC instrument (MicroCal), calibrated according to the manufacturer's instructions. Protein and RNA samples were dialysed against the NMR buffer (where the DTT was substituted by TCEP). Concentrations of proteins and RNAs were determined using optical density absorbance at 280 and 260 nm, respectively. 20 µM of each RNA was titrated with 180 µM of GB1‐SRSF1ΔRS protein by 40 injections of 6 µl every 5 min at 27°C. Raw data were integrated, normalized for the molar concentration and analysed using the Origin 7.0383 software according to a 1:1 RNA:protein ratio‐binding model.

### U1 snRNA rescue assay on *SMN1* exon7

The SMN1 minigene is encoded by the pCI‐SMN1 plasmid (Lorson *et al*, 1999). It contains the following 5′SS GGA\GUAAGUCU at the 3′‐end of exon 7. In order to make this splicing event independent of the endogenous U1 snRNP, the 5′SS was mutated to GGA\GUAUGUCU (mutation A + 4 U). To rescue the splicing, U1 snRNA containing the complementary mutation was expressed from a modified pG3‐U1 snRNA (Raponi *et al*, 2009) containing the mutation U_5_A. Since the rescue was only partial, we also closed the bulged created by the unpaired adenine in position −1 by adding the mutation C_10_U on pG3‐U1 U_5_A. Using this plasmid, the splicing is driven by the exogenous U1 snRNA and the effects of mutations on stem‐loop 3 were investigated. The mutations C_98–101_A and G_108–110_U were introduced into pG3‐U1 snRNA U_5_A C_10_U, which disturb the CA and GGA motifs but maintain the base‐pairing. All the mutations were introduced using a quick change protocol, and oligonucleotide sequences are available upon request.

One microgram of pCI‐SMN1 and pG3‐U1snRNA was transfected in HEK293T cells using the Lipofectamine 2000 Reagent (Life Technologies) according to the manufacturer's protocol. The cells were previously counted and seeded 24 h in advance at 400,000 cells/well. Cells were harvested after 40–44 h. Total RNA was extracted, and 1 µg was used for reverse transcription using Oligo(dT)15 primer and M‐MLV Reverse Transcriptase (RNase H‐; Promega). 10% of the resulting cDNA was then used for semiquantitative PCR using a vector‐specific forward primer (pCI‐fwd 5′‐GGTGTCCACTCCCAGTTCAA‐3′) and a SMN1‐specific reverse primer (SMN1rev 5′‐GCCTCACCACCGTGCTGG‐3′). The PCR products were separated on a 4% polyacrylamide gel at 100 V for 1 h and stained with GelRed (Biotium). Band integration was performed Image Studio Lite (LI‐COR), and the ratio between both isoforms for each condition was determined. The experiment was performed five times.

## Author contributions

ICE, FHTA and AJH conceived and supervised the study; AMJ, RW and CML did single‐molecule experiments; AJH and RW built the microscope; RW conceived, designed and wrote the MATLAB code; SC prepared *in vitro* reconstituted U1 snRNP and did the NMR experiments and mutant analyses; ARG did the bioinformatic analyses; AC prepared SRSF1 proteins; LC, LPE and MJH did additional experiments; and ICE and SC wrote the manuscript with inputs from AMJ, RW, ARG, AC and FHTA.

## Conflict of interest

The authors declare that they have no conflict of interest.

## Supporting information



Appendix

## Data Availability

The data supporting this publication are available from the University of Leicester's Figshare data repository https://doi.org/10.25392/leicester.data.c.5521944. The single‐molecule image data in the Appendix are available upon request.

## Footnotes

^§^
Correction added on the 4^th^ of January 2022, after first online publication: the x‐axis label has been corrected from “time (min)” to “time (frames)”.
