# Supplementary material for: Exon‐independent recruitment of SRSF1 is mediated by U1 snRNP stem‐loop 3
Source: EMBO J. 2021 Nov 15;41(1):e107640. doi: 10.15252/embj.2021107640 (PMC8724738; doi:10.15252/embj.2021107640)
Supplement: Supplementary file 1 — Appendix [file EMBJ-41-e107640-s001.pdf]

Appendix

Exon-independent recruitment of SRSF1 is mediated by U1 snRNP stem-loop 3

Andrew M. Jobbins†, Sébastien Campagne†, Robert Weinmeister, Christian M. Lucas, Alison R. Gosliga, Antoine Clery, Li Chen, Lucy P. Eperon, Mark J. Hodson, Andrew J. Hudson, Frédéric H.T. Allain* and Ian C. Eperon*

Table of contents

Appendix Table 1: List of genes with proteins represented in Appendix Fig. S10.

Appendix Fig. S1 and legend: Characterization of mEGFP-SRSF1 expression and activity.

Appendix Fig. S2 and legend: Frequency histograms showing the distributions of bleaching steps expected if all complexes contained one or two molecules of SRSF1.

Appendix Fig. S3 and legend: The level and patterns of binding of mEGFP-SRSF1 in the absence of ATP depend on U1 snRNA binding to 5′SS and on protein phosphatases.

Appendix Fig. S4 and legend: Splicing and complex assembly by RNA labelled with Cy5-maleimide.

Appendix Fig. S5 and legend: Frequency histograms showing the levels of two-way co-localization of mEGFP-SRSF1 and mCherry-U1A in a nuclear extract in the absence of exogenous pre-mRNA after treatment with ribonucleases A and T1.

Appendix Fig. S6 and legend: Addition of a 5′SS to the 3′ end of BGSMN2 is as effective as the addition of tandem copies of a strong ESE.

Appendix Fig. S7 and legend: Effects of a 3′-terminal 5′SS on the recruitment of U2AF35, U2AF65 and U2 snRNP to BGSMN2-U1 pre-mRNA.

Appendix Fig. S8 and legend: Colocalization of mEGFP-SRSF1ΔRS on GloC and BGSMN2 + ESE-Ax4.

Appendix Fig. S9 and legend: SL3 mutations impair the binding of SRSF1[illegible]RS but maintain the interactions with FUS RRM and PTBP1 RRM1.

Appendix Fig. S10 and legend: Protein cross-links to U1 snRNA *in vivo*.

Appendix Fig. S11 and legend: Complexes identified by bleaching as containing two molecules of mEGFP-SRSF1 emit approximately twice as many photons as those identified as containing one molecule.

Appendix Table S1. List of genes with proteins represented in Appendix Fig. S1

1. AATF
2. ABCF1
3. AGGF1
4. AKAP1
5. AKAP8L
6. AQR
7. BCCIP
8. BCLAF1
9. BUD13
10. CDC40
11. CPEB4
12. CPSF6
13. CSTF2
14. CSTF2T
15. DDX21
16. DDX24
17. DDX3X
18. DDX42
19. DDX51
20. DDX52
21. DDX55
22. DDX59
23. DDX6
24. DGCR8
25. DHX30
26. DKC1
27. DROSHA
28. EFTUD2
29. EIF3D
30. EIF3G
31. EIF3H
32. EIF4G2
33. EWSR1
34. EXOSC5
35. FAM120A
36. FASTKD2
37. FKBP4
38. FMR1
39. FTO
40. FUBP3
41. FUS
42. FXR1
43. FXR2
44. G3BP1
45. GEMIN5
46. GNL3
47. GPKOW
48. GRSF1
49. GRWD1
50. GTF2F1
51. HLTF
52. HNRNPA1
53. HNRNPC
54. HNRNPK
55. HNRNPL
56. HNRNPM
57. HNRNPU
58. HNRNPUL1
59. IGF2BP1
60. IGF2BP2
61. IGF2BP3
62. ILF3
63. KHDRBS1
64. KHSRP
65. LARP4
66. LARP7
67. LIN28B
68. LSM11
69. MATR3
70. METAP2
71. MTPAP
72. NCBP2
73. NIP7
74. NIPBL
75. NKRF
76. NOL12
77. NOLC1
78. NONO
79. NPM1
80. NSUN2
81. PABPC4
82. PABPN1
83. PCBP1
84. PCBP2
85. PHF6
86. POLR2G
87. PPIG
88. PPIL4
89. PRPF4
90. PRPF8
91. PTBP1
92. PUM1
93. PUM2
94. PUS1
95. QKI
96. RBFOX2
97. RBM15
98. RBM22
99. RBM5
100. RPS11
101. RPS3
102. SAFB2
103. SAFB
104. SBDS
105. SDAD1
106. SERBP1
107. SF3A3
108. SF3B1

109. SF3B4
110. SFPQ
111. SLBP
112. SLTM
113. SMNDC1
114. SND1
115. SRSF1
116. SRSF7
117. SRSF9
118. SSB
119. SUB1
120. SUGP2
121. SUPV3L1
122. TAF15
123. TARDBP
124. TBRG4
125. TIA1
126. TIAL1
127. TRA2A
128. TROVE2
129. U2AF35
130. U2AF65
131. UCHL5
132. UPF1
133. UTP18
134. UTP3
135. WDR3
136. WDR43
137. WRN
138. XPO5
139. XRCC6
140. XRN2
141. YBX3
142. YWHAG
143. ZC3H11A
144. ZC3H8
145. ZNF622
146. ZNF800
147. ZRANB2

Appendix Fig. S1

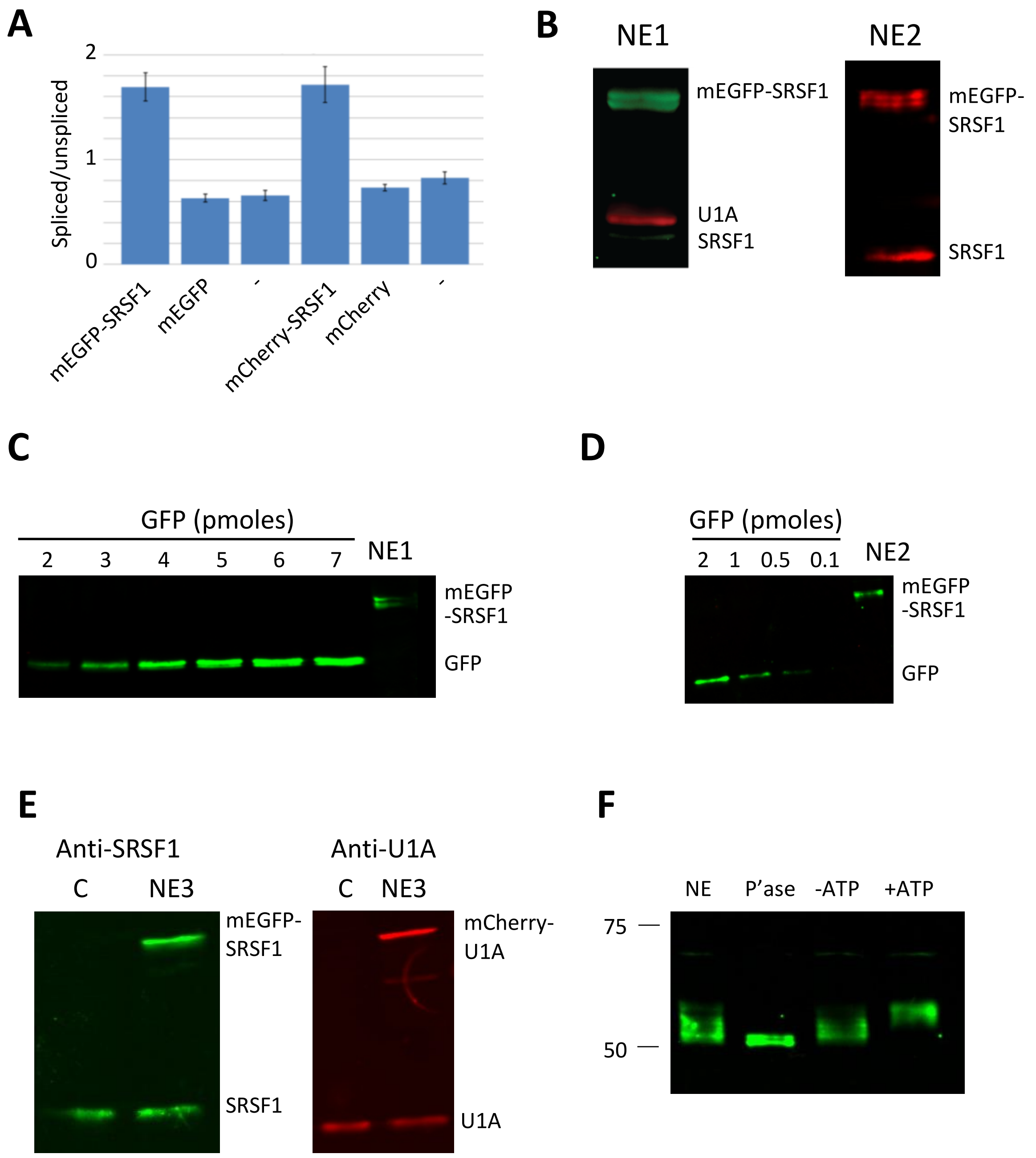

Appendix Fig. S1. Characterization of mEGFP-SRSF1 expression and activity. (**A**) Effects of transfection of plasmids expressing mEGFP-SRSF1 or mCherry-SRSF1 on the ratio of spliced to unspliced intron 4 of endogenous SRSF1 mRNA in HeLa cells (Sun *et al,* 2010). Empty vector and negative controls are shown also. PCR products were analysed by agarose gel

electrophoresis and stained with ethidium bromide before imaging. Experiments were biological triplicates; the error bars show the standard errors of the mean. (**B**) Western blot of SRSF1 in nuclear extracts NE1 and NE2. Fluorescent antibodies were detected in an Odyssey CLx (LI-COR). Endogenous U1A was detected in parallel with NE1 as a control. (**C**) Quantification of expression in NE1 by western blotting using anti-GFP antibody and known quantities of pure GFP. (**D**) As (C), with NE2. (**E**) Western blot of SRSF1 in the extract (NE3) in which both mEGFP-SRSF1 and mCherry-U1A were expressed. (**F**) Western blot with anti-GFP antibodies on samples from NE1, containing mEGFP-SRSF1: untreated nuclear extract (NE), nuclear extract treated with alkaline phosphatase (P'ase), nuclear extract incubated to deplete ATP (-ATP) and nuclear extract incubated under splicing conditions with ATP (+ATP).

Appendix Fig. S2

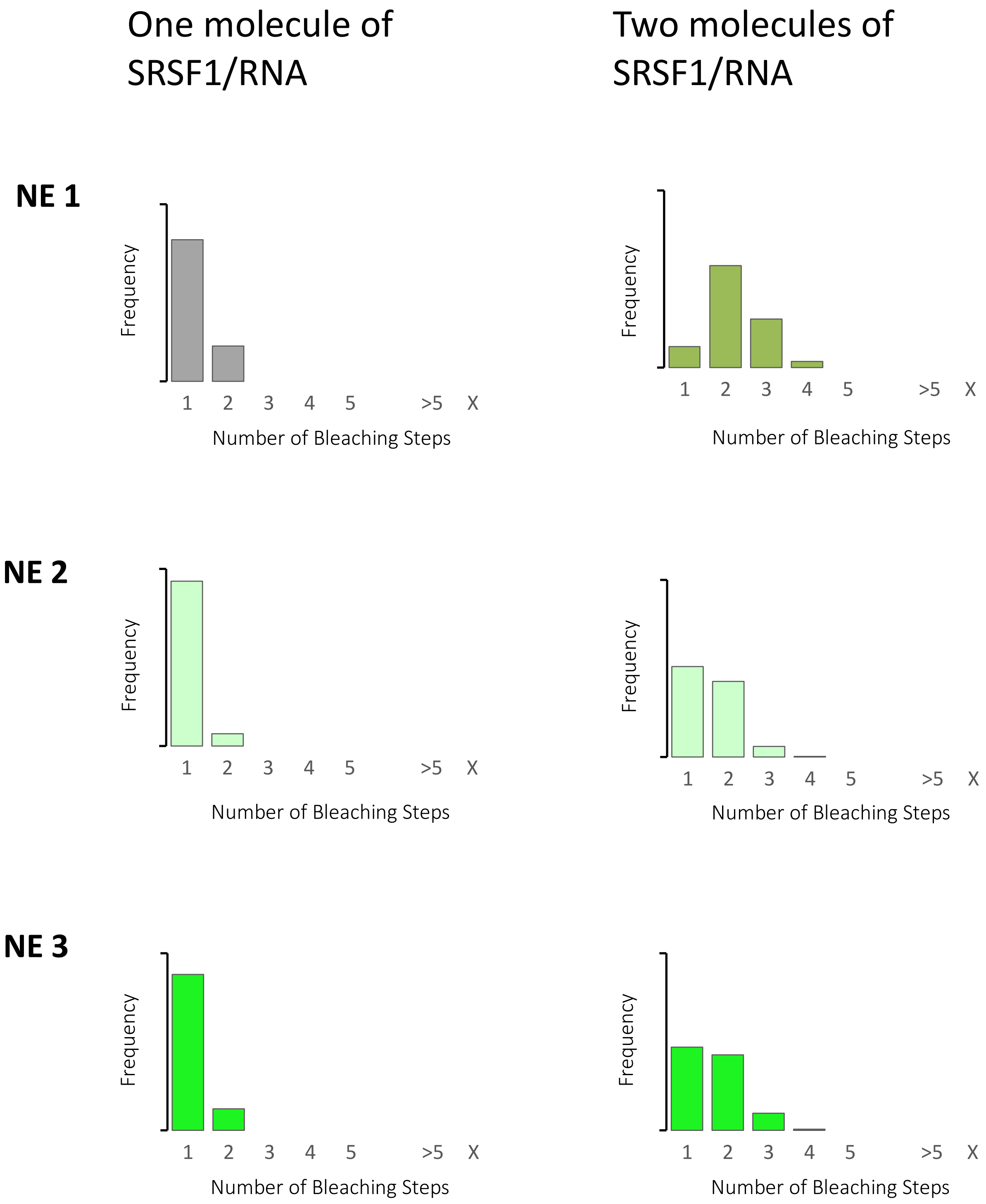

Appendix Fig. S2. Frequency histograms showing the distributions of bleaching steps expected if all complexes contained one or two molecules of SRSF1. The distributions expected are calculated from the relative levels of mEGFP-SRSF1 and endogenous SRSF1 in each extract and the observed levels of dimerization. (Table 1).

Appendix Fig. S3

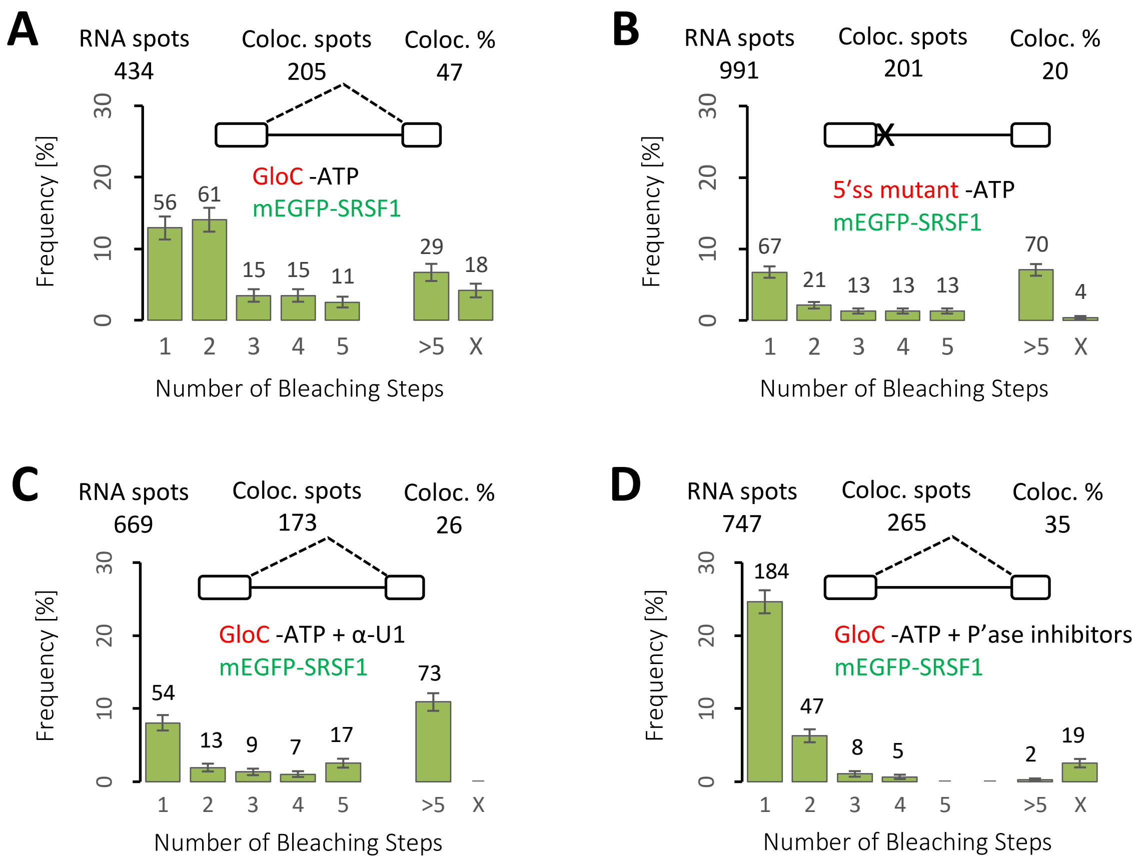

Appendix Fig. S3. The level and patterns of binding of mEGFP-SRSF1 in the absence of ATP depend on U1 snRNA binding to 5'SS and on protein phosphatases. (**A**) Distributions of numbers of molecules of mEGFP-SRSF1 associated with single molecules of GloC pre-mRNA after depletion of ATP by pre-incubation prior to the addition of ATP. (**B**) Association of mEGFP-SRSF1 on pre-mRNA in which the 5'SS had been inactivated by mutation. (**C**) Association with GloC pre-mRNA after sequestration of U1 snRNA with a complementary oligonucleotide. (**D**) Distribution on GloC pre-mRNA when ATP depletion had been done in the presence of protein phosphatase inhibitors.

Appendix Fig. S4

A

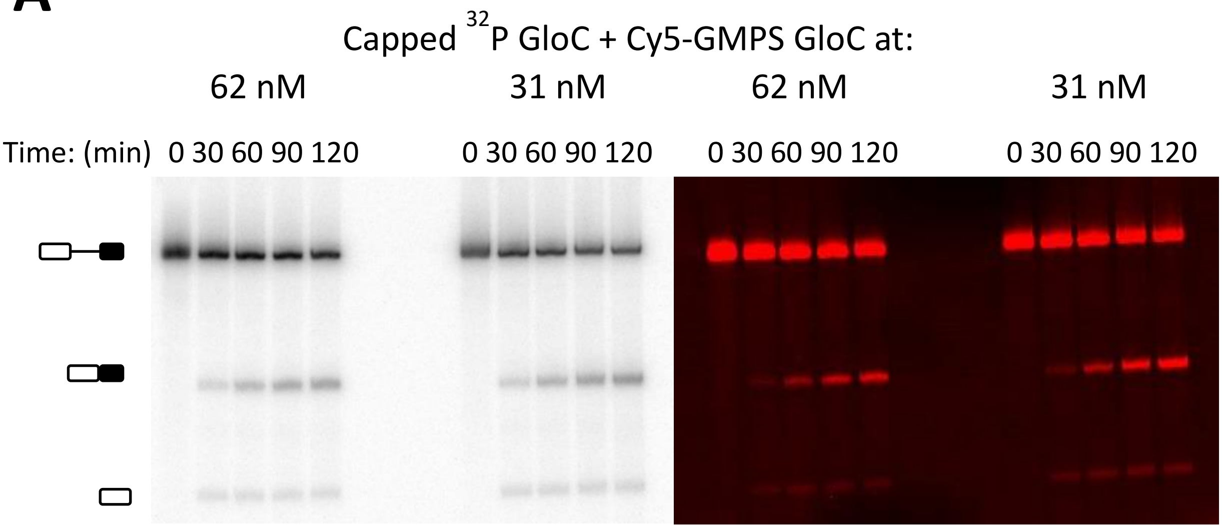

B

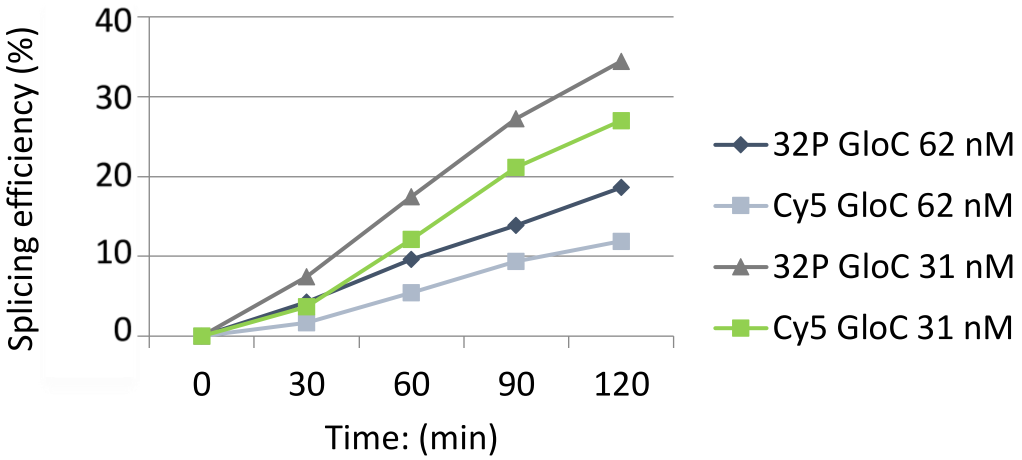

C

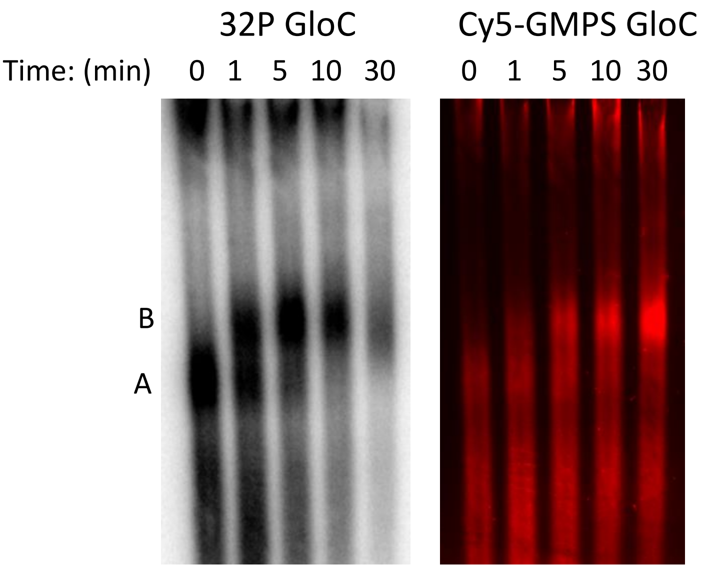

Appendix Fig. S4. Splicing and complex assembly by RNA labelled with Cy5-maleimide. (**A**) Normal ^{32}P-labelled GloC pre-mRNA, with a 5′ cap, and Cy5-labelled pre-mRNA were mixed and incubated at ~1 nM and either 31 or 62 nM, respectively, in nuclear extract under splicing conditions. Samples were removed at the times indicated and analysed by gel electrophoresis. Gels were imaged to detect both fluorescence and radioactivity. (**B**) Splicing efficiency was taken as [mRNA]/([pre-mRNA]+[mRNA]+[5′ exon intermediate]) at each time point. (**C**) Analysis by native gel electrophoresis of the formation of heparin-resistant complexes in reactions done as above with transcripts labelled with ^{32}P or Cy5 (62 nM).

Appendix Fig. S5

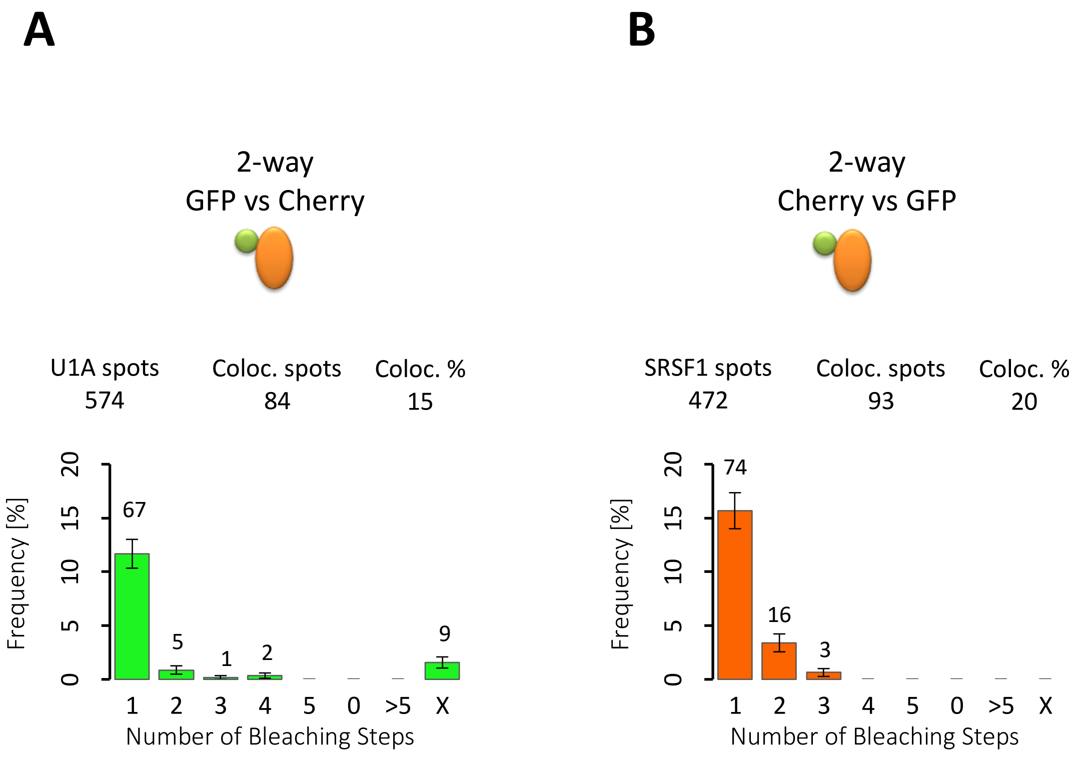

Appendix Fig. S5. Frequency histograms showing the levels of two-way co-localization of mEGFP-SRSF1 and mCherry-U1A in a nuclear extract in the absence of exogenous pre-mRNA after treatment with ribonucleases A and T1. (**A**) Association of mEGFP-SRSF1 with mCherry-U1A. Of 574 mCherry-U1A spots, 84 were colocalised with mEGFP-SRSF1, and in 67 of the 84 cases there was only a single molecule of mEGFP-SRSF1. (**B**) Association of mCherry-U1A with mEGFP-SRSF1. Of 472 mEGFP-SRSF1 spots, 93 were colocalised with mCherry-U1A, and in 74 of the 93 cases there was only a single molecule of mCherry-U1A.

Appendix Fig. S6

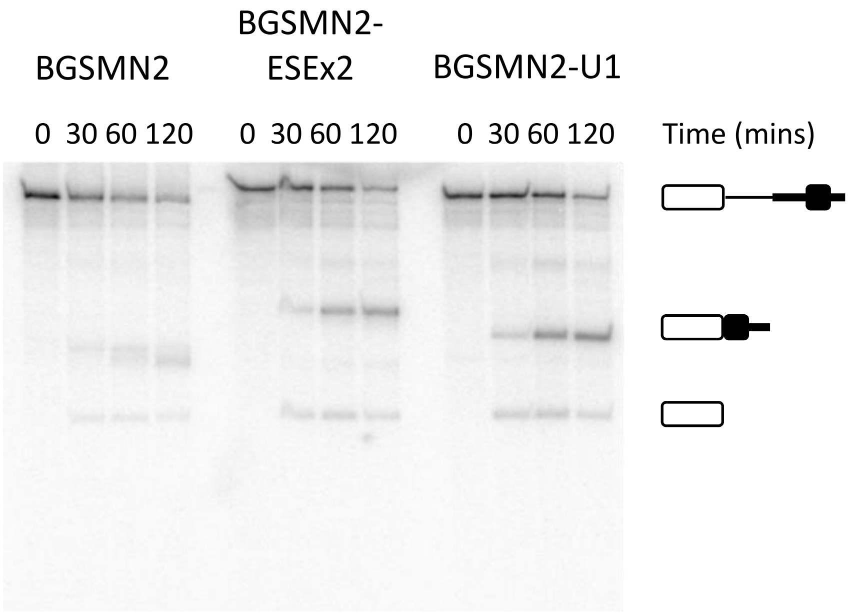

Appendix Fig. S6. Addition of a 5'SS to the 3' end of BGSMN2 is as effective as the addition of tandem copies of a strong ESE. The pre-mRNA substrates indicated were incubated in nuclear extract for the times shown and the reactions analysed by denaturing polyacrylamide gel electrophoresis.The pre-mRNA, mRNA and 5' exon intermediate are shown. The addition of the 3' ESE sequences and the U1 binding site increased the lengths of the pre-mRNA and the mRNA but not that of the 5' exon intermediate. The effects of adding multiple copies of this ESE sequence to this pre-mRNA is documented fully elsewhere (Jobbins *et al,* 2018).

Appendix Fig. S7

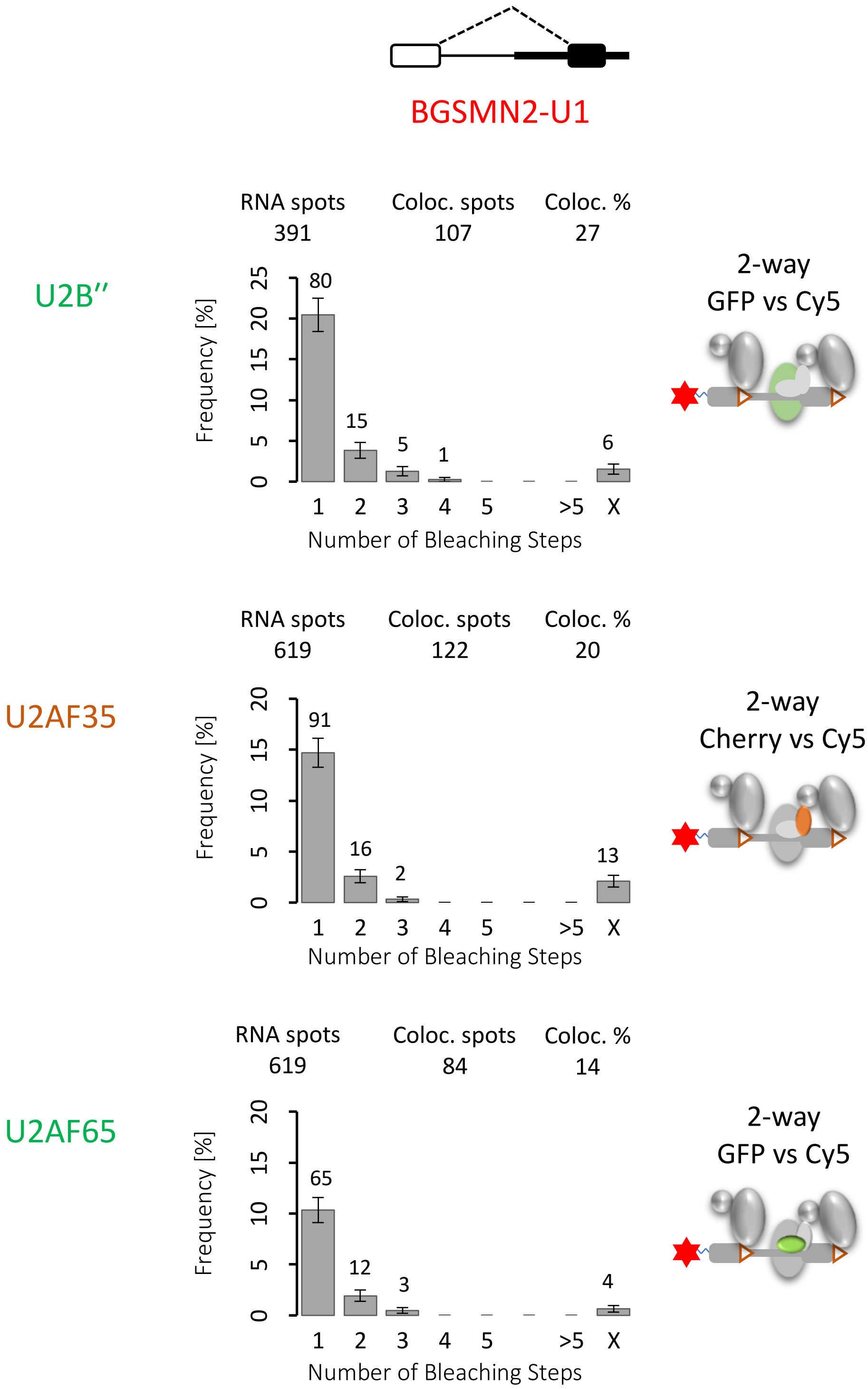

Appendix Fig. S7. Effects of a 3′-terminal 5′SS on the recruitment of U2AF35, U2AF65 and U2 snRNP to BGSMN2-U1 pre-mRNA. The U2 snRNP was detected by using a nuclear extract from HeLa cells expressing mEGFP-U2B″, and U2AF35 and U2AF65 were detected using a single extract from cells in which mCherry-U2AF35 and mEGFP65 had been expressed (Chen *et al,* 2017). Two-way colocalization experiments were done with each extract and Cy5-labeled pre-mRNA. The interpretative cartoons on the right are coloured to indicate the components labeled and detected in each experiment.

Appendix Fig. S8

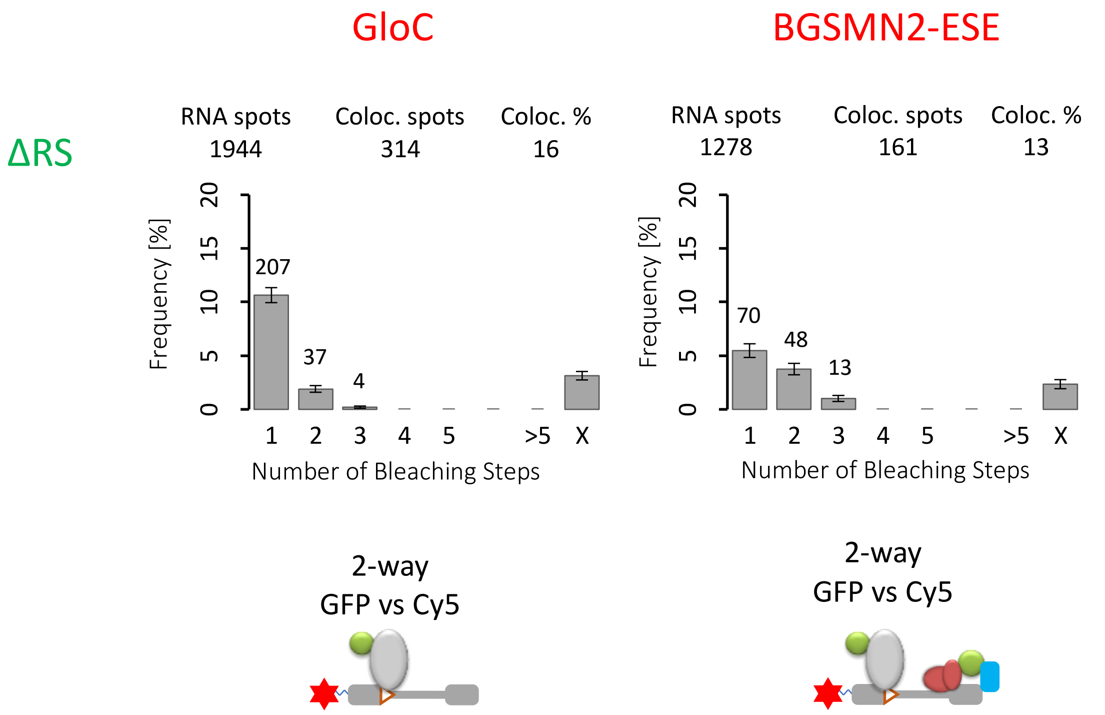

Appendix Fig. S8. Colocalization of mEGFP-SRSF1ΔRS on GloC and BGSMN2 + ESE-Ax4. Nuclear extract expressing mEGFP-SRSF1ΔRS was incubated with GloC or BGSMN2-ESE Cy5-pre-mRNA, amd two-way colocalization was done to measure the number of steps in which the mEGFP colocalised with the pre-mRNA was bleached.

Appendix Fig. S9

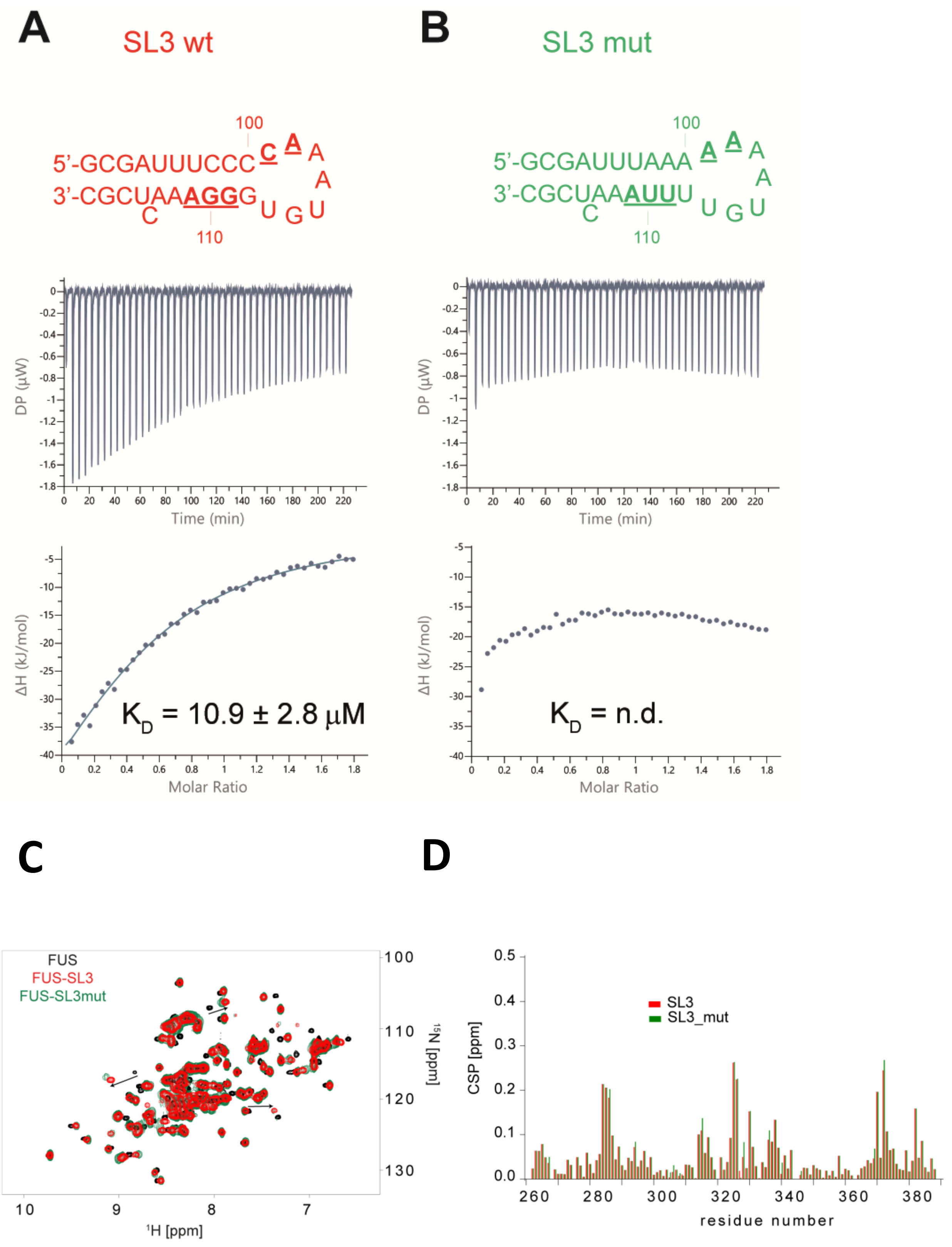

Appendix Fig. S9. SL3 mutations impair the binding of SRSF1ΔRS but maintain the interactions with FUS RRM. (**A**) Isothermal titration calorimetry of the binding of SRSF1ΔRS on U1 snRNA SL3. (**B**) Isothermal titration calorimetry of the binding of SRSF1ΔRS on U1 snRNA SL3 mutant. (**C**) Overlay of the 2D ^{1}H-^{15}N HSQC spectra of FUS RRM before (black) and after addition of 1.3 molar equivalent of SL3 wild type (red) or SL3 mutant (green). (**D**) Plot of the normalized chemical shift perturbations (CSP) of the FUS amide resonances upon titration with SL3 wild-type (red) or SL3 mutant (green).

Appendix Fig. S10

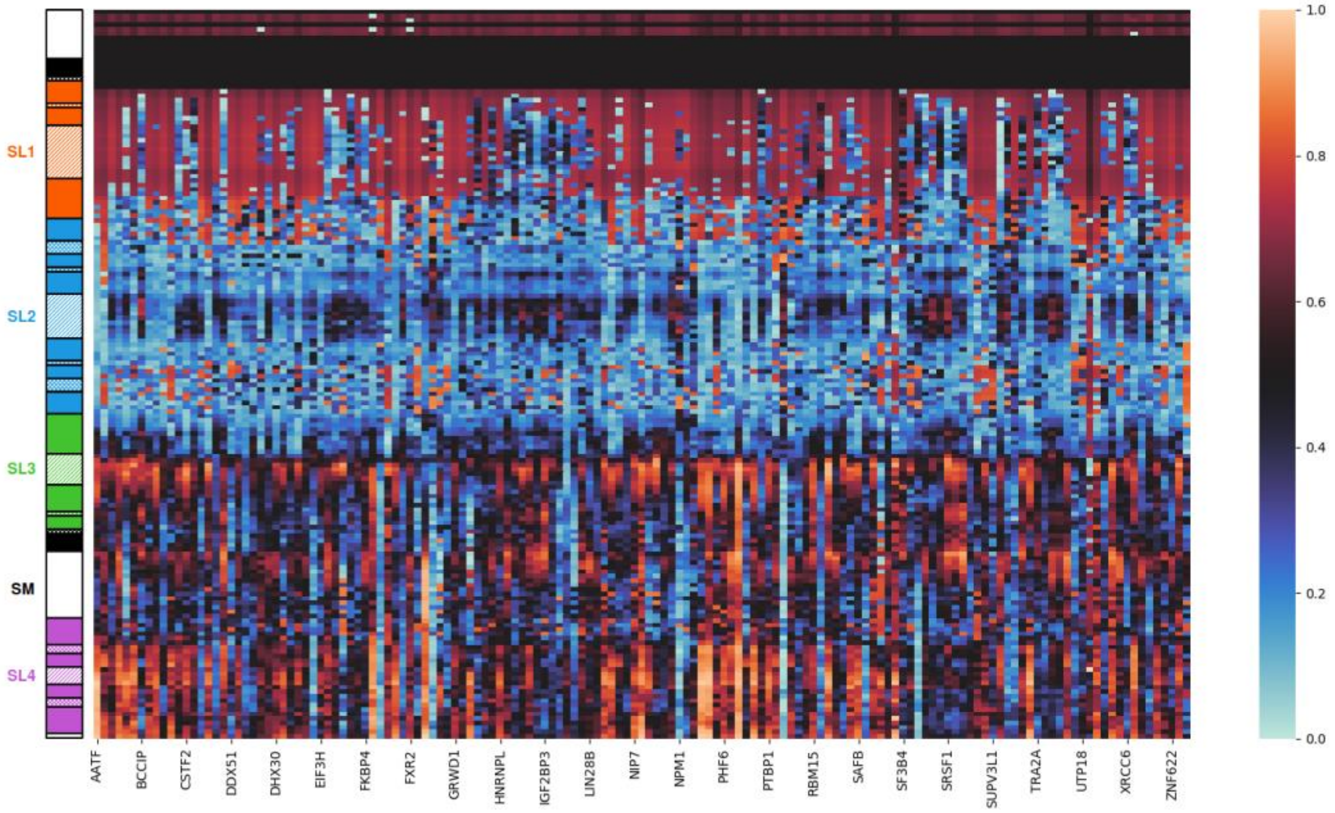

Appendix Fig. S10. Protein cross-links to U1 snRNA *in vivo*. The heat map shows the distribution of cross-links to U1 snRNA for 147 RNA-binding proteins, from ENCODE project data (Van Nostrand *et al,* 2020). U1 snRNA is represented along the ordinate. The strategy used in the experiments precluded the observation of cross-links at the 5′ end of U1 snRNA. A list of all the proteins represented is included in Appendix Table 1.

Appendix Fig. S11

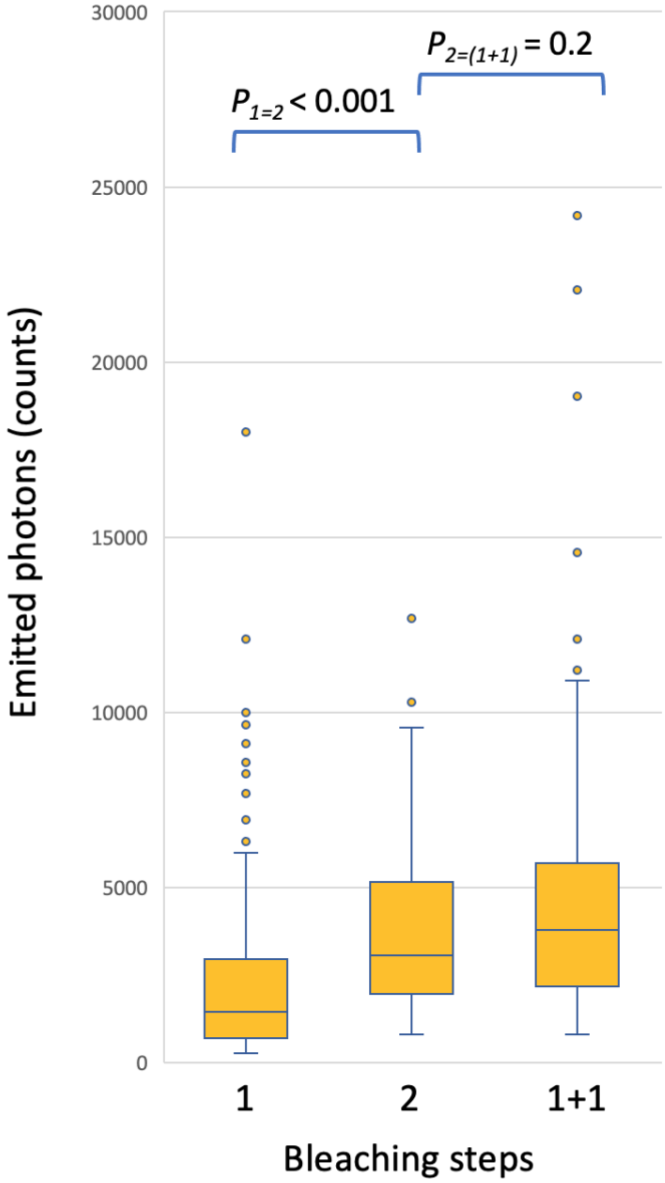

Appendix Fig. S11. Complexes identified by bleaching as containing two molecules of mEGFP-SRSF1 emit approximately twice as many photons as those identified as containing one molecule. The total counts emitted upon irradiation at 488 nm was measured for every spot counted in Figure 3B (mEGFP-SRSF1 colocalised with pre-mRNA GloC-U1). The background measured in the same area after bleaching was subtracted. The counts detected from individual complexes containing one or two molecules of mEGFP-SRSF1 are plotted as a box plots, labelled 1 or 2 respectively. In the plot labelled 1+1, values from the single molecules complexes in plot 1 were shuffled at random and added pairwise to the

values in plot 1, simulating complexes with two molecules of SRSF1. The median values are shown as a line in each box, and the values are (1) 1,472, (2) 3,085 and (1+1) 3,802.
