# Supplementary material for: Exon‐independent recruitment of SRSF1 is mediated by U1 snRNP stem‐loop 3
Source: EMBO J. 2021 Nov 15;41(1):e107640. doi: 10.15252/embj.2021107640 (PMC8724738; doi:10.15252/embj.2021107640)
Supplement: Supplementary file 2 [file EMBJ-41-e107640-s002.pdf]

THE EMBO JOURNAL

Exon-independent recruitment of SRSF1 is mediated by U1 snRNP stem-loop 3

Andrew Jobbins, Sébastien Campagne, Robert Weinmeister, Christian Lucas, Alison Gosliga, Antoine Cléry, Li Chen, Lucy Eperon, Mark Hodson, Andrew Hudson, Frederic Allain, and Ian Eperon
DOI: 10.15252/embj.2021107640

Corresponding author(s): Ian Eperon (eci@leicester.ac.uk) , Ian Eperon (eci@leicester.ac.uk), Frederic Allain (allain@mol.biol.ethz.ch)

Review Timeline:

Submission Date:	4th Jan 21
Editorial Decision:	11th Feb 21
Revision Received:	16th Jul 21
Editorial Decision:	9th Sep 21
Revision Received:	24th Sep 21
Editorial Decision:	29th Sep 21
Revision Received:	4th Oct 21
Accepted:	7th Oct 21

Editor: Stefanie Boehm

Transaction Report:

(Note: With the exception of the correction of typographical or spelling errors that could be a source of ambiguity, letters and reports are not edited. Depending on transfer agreements, referee reports obtained elsewhere may or may not be included in this compilation. Referee reports are anonymous unless the Referee chooses to sign their reports.)

1st Editorial Decision **11th Feb 2021**

Prof. Ian C. Eperon
Leicester, University of
Molecular and Cell Biology
University of Leicester
Henry Wellcome Building, Lancaster Road
Leicester, UK-Leicester LE1 9HN
United Kingdom

11th Feb 2021

Re: EMBOJ-2021-107640
Exon-independent recruitment of SRSF1 is mediated by U1 snRNP stem-loop 3

Dear Prof. Eperon,

Thank you for submitting your manuscript for consideration by The EMBO Journal. We have now however received comments on your study from three experts, which are included below for your information.

As you will see, the reviewers are overall positive and acknowledge the interest in the proposed model for SRSF1 and U1 in splicing. However, they do raise some concerns, which will need to be addressed in a revised version. Specifically, further characterization and comparison of SRSF1 binding modes by additional experimental analyses and/or expanding the discussion should be included as indicated (ref#1- point 1,2; ref#2- point 2; ref#3- point 3, 4). In addition, the technical concerns and issues with insufficient explanation of the experimental setup or data analysis must be resolved (ref#1- point 3; ref#2- point 3, 4, 5, 6, 7; ref#3- point 1, 2). Referee #2's points 8 and 9 should also be discussed, and the manuscript overall carefully revised for readability. Please also consider all other referee comments and revise the manuscript accordingly. If you are able to resolve the key concerns and satisfactorily respond to issues raised by the reviewers, we will be happy to consider the study further for publication. Therefore I would now like to invite you to prepare and submit a revised manuscript.

Please note that it is our policy to allow only a single round of major revision. We realize that lab work worldwide is currently affected by the COVID-19/SARS-CoV-2 pandemic and that an experimental revision may be delayed. We can extend the revision time when needed, and we have extended our 'scooping protection policy' to cover the period required for a full revision. However, it is nonetheless important to clarify any questions and concerns at this stage and we encourage you to discuss a revision plan and any potential issues you may foresee as soon as possible.

When submitting the revised version, we would also like to ask you to reassess the list of corresponding authors, which currently includes four persons. We realize that interdisciplinary and international studies may involve several laboratories conducting experiments independently; we also understand the perceived importance of authorship position for academic credit. However, corresponding authors have an exceptional responsibility regarding the manuscript submission and publication process and in post-publication communication and accountability for the data. Corresponding authorship should not be equated with 'senior authorship'. Thus, we would like to ask you to reconsider to which degree the criteria for corresponding authorship are met in all four cases. Please note that the Author Contribution section of the manuscript allows authors to explicitly define their individual contributions in a more granular fashion.

Please also feel free to contact me should you have any other further questions. Thank you for the opportunity to consider your work for publication. I look forward to receiving your revised manuscript.

Kind regards,

Stefanie Boehm

Stefanie Boehm
Editor
The EMBO Journal

Referee #1:

In this manuscript, the authors probe the binding targets of the splicing enhancer SRSF1. Using quantitative single-molecule imaging, the authors uncover a new mode of SRSF1 binding in which the protein targets stem loop 3 on the U1 snRNP. Potentially, this observation is an impactful conceptual advancement in our understanding of spliceosome assembly. A new issue is raised however: if U1 is capable of independent recruitment of SRSF1, how does one achieve exon definition? A few comments need to be addressed before the manuscript can be published.

Major comments:

(1) Compared to the established binding mode in which SRSF1 binds exonic sequences independently of the U1 snRNP, how strongly does SRSF1 bind stem loop 3 of U1? The single molecule stoichiometry experiments indirectly suggest that the binding affinities of these two distinct substrates could be quite similar however quantitative measurements of binding affinity are missing from this manuscript. The authors should perform in vitro binding assays to quantify the relative binding affinity of SRSF1 RS to the U1 snRNP (or just stem loop 3) compared to a typical exonic binding site for this protein. No matter the outcome, this result would provide insight into the relative prevalence of these binding modes. Furthermore, in vitro binding assays should also be performed to the stem loop mutant used in figure 4 C to show that this mutation really does prevent SRSF1 binding.

(2) In figure 4C the authors argue that that efficient splicing requires SRSF1 in complex with stem loop 3 of the U1 snRNP. However, the authors should address (1) the impact of this mutation on the binding of other proteins, namely FUS and PTBP1 which appear to target stem loop 3 as well and (2) the impact of this mutation on U1 structure and its implications for spliceosome assembly. As the manuscript stands, figure 4C does not convincingly measure the contribution of SRSF1 to the overall reduction in splicing observed in condition 5. Therefore, this manuscript would benefit from a more convincing link between SRSF1/U1 heterodimerization and splicing efficiency.

(3) The section titled, "The association of U1 and SRSF1 does not require pre-mRNA" requires further clarification. In this experiment, why is it not possible that U1 and SRSF1 are in complex with unlabeled endogenous mRNA from the nuclear extract?

Minor comments:

(1) Figure 1A: While the authors performed a rigorous analysis of the number of photobleaching steps for SRSF1-mEGFP and U1-mCherry they also need to perform this same analysis for Cy5 mRNA molecules. I believe the authors only assume that each that Cy5 spot corresponds to only a single mRNA, however this may not be the case and could significantly change the interpretation of the data. Furthermore, the authors illustrate their photobleaching analysis workflow with two representative traces. It is preferable to include a figure that illustrates the overall quality of all their traces. This can be done with a histogram that shows the distribution of intensities for all analyzed spots (Cy5, mEGFP, and mCherry). One would expect to see multiple peaks corresponding to different binding stoichiometries. Another potential option is to use transition density plots (https://github.com/ebfret/TransitionDensityPlots).

(2) Figure 1A: The images look strangely thresholded. The authors should include what (if any) image filtering they used.

(3) The figure legends right now are too minimal. This is very apparent in figure 4 which, in many ways is similar to figure 1 in Roca et al. (PMID: 22588721). The figure legend should have a similar amount of description as this publication to improve readability.

(4) A number of the figures (2C, 3B) appear to be from screenshots. Remove the artifacts. Figure 3B (top right) also has some formatting issues.

(5) Old and new nomenclature are occasionally used interchangeably (U2AF2 / U2AF65).

Referee #2:

In this work Jobbins and colleagues use a single molecule approach to study U1 complexes deposited on RNAs and find that frequently they colocalize with the SRSF1 factor--ultimately suggesting that SRSF1 can be a constitutive component of the snRNP. They complement these assays with some functional assays, biophysical studies of a potential SRSF1/U1 snRNA interaction, and analysis of eCLIP data. The manuscript data are robust for supporting the central conclusions.

Significantly, the authors provide good evidence for the existence of a "holo"-U1 snRNP that minimally contains SRSF1 in addition to the canonical members. This suggests that canonical U1 may really just be the apo-snRNP and only part of the splicing reaction when in complex with other members. This is a very important addition to the field and will be of interest to a large number of readers interested in splicing mechanism and regulation. In addition, the paper represents a technical tour-de-force and enviable synthesis of multiple experimental methods (single molecule, NMR, CLIP-Seq, in vitro biochemistry and molecular biology) to arrive at their final model. At this stage, however, there are a number of issues which need to be addressed and clarified before publication. Nonetheless, this work is of high interest and the science compelling.

Major Issues

1. Overall the data presentation is challenging. I think the authors may want to consider how that contributes to the overall readability of the manuscript.There were also a number of key experiments in the supplemental that may be better in the main text. I particularly found the result showing that the RS domain wasn't responsible solely for the RNA colocalization was interesting (Figure S6).

2. I think the data and resulting model are over-interpreted. While the author's provide compelling evidence for a U1-SRSF1 complex, they have no data that specifically excludes other mechanisms such as that in 5A. I think the authors should conclude that their mechanism is an alternate pathway by which U1 can be recruited and the predominant pathway that may occur within a cell or on a particular transcript needs further study. Similarly on line 436, this seems a vast overstatement. I would suggest "U1 snRNP might not play a direct role in all splicing reactions but could also act..."

3. The experimental setup described on page 5 is inadequate. First, how was functional monomeric SRSF1 confirmed? I'm assuming this is based on the data in S1A, however this isn't clearly described and it is unclear if this is an in vivo or in vitro assay. Why do extracts contain different amounts of mGFP-SRSF1? Does the amount of mEGFP-SRSF1 present correlate with the amount of spliced/unspliced using the assay in Figure S1A? Were the U1 molecules attached to the pre-mRNAs under conditions that fully support splicing? How was U6 ablation confirmed--by in vitro splicing assays or Northern blotting against the U6 snRNA? In our hands, the amount of U6 oligo needed to ablate the activity is extract dependent. Finally, were the RNA molecules analyzed single molecules of RNA? Or is it possible that some RNA spots being analyzed were actually clusters of multiple RNAs either containing non-fluorescent RNA molecules or those unable to resolved by the microscope as individual spots?

4. A major experiment in the paper was showing that association of U1 and SRSF1 can occur without pre-mRNA. However, no details were included about how that experiment was carried out. Is it possible that this interaction occurs mainly in vitro? What happens if a NE containing only fluroescent U1 (and non fluorescent but over-expressed SRSF1) was combined with a NE containing nonfluorescent U1 and over expressed fluorescent SRSF1? Would colocalization still be observed? If so, that may reveal some information about the relative stabilities and dynamics of the U1/SRSF1 complex.

5. Another major experiment is showing that the U1/SRSF1 complexes can recruit U2. This is important since it implies that the U1/SRSF1 complexes being studied are chemically competent for splicing and advancing through the reaction. However, this experiment was very poorly described and this reviewer could not make sense of the data in Table 2. Do U1/SRSF1 complexes also colocalize with U2? If this cannot be measured directly, then this should be noted in the manuscript and the competence of the studied complexes needs to be made less certain.

6. Table 1 is a bit confusing. %labeled:unlabeled perhaps should be %FP tagged/endogenous. The critical value for interpretation of the data is the % fluorescent complexes (# fluorescent molecules/total molecules). This is not necessarily the same as the FP tagged percentage since not all FP chromophores will mature. Can the authors correct for the maturation percentage? Does this impact the data interpretation? In addition, some values were listed as "n.d." since the levels were equal to the endogenous protein. It is unclear to me why this would be "n.d." for mEGFP-SRSF1 for NE3 then but not for NE2?

7. It seems like the distribution of likely U1-dependent SRSF1 spots in Figure 1B can be obtained by subtracting the dstribution

in 1 C with anti-U1 from the distribution in 1B with GloC. Similarly, the key analysis for interpretation of the data in 1B from the RNA showing 2 introns would subtraction of a distribution observed from the same RNA under anti-U1 conditions. Would that still show a sizable number of 2 photobleaching spots? Without the 2 intron control, I'm unsure how to interpret the data from that RNA.

8. Could apo and holo U1 (U1 +SRSF1) have different functions in the cell? Perhaps one is primarily involved in splicing and the other telescripting?

9. I think it would be worth surveying the literature on characterization of U1--given that many groups have purified U1 from human cells for decades has there ever been any evidence of co-purification of SRSF1?

Minor Issues:

1. Abstract line 31, maybe "other" components at the 3'SSs
line 35, SRSF1 "can be" recruited by a U1 snRNP

2. line 151 "was restricted and specific rather than stochastic" I am unsure what this means.

3. Line 154, I found this paragraph very confusing

4. Line 259, is work from the Zhao lab on the yeast E complex relevant for thinking about how cross-exon complexes form? (Nature. 2019 Sep;573(7774):375-380. doi: 10.1038/s41586-019-1523-6)

5. Discussion: line 352 "can associate", "SRSF1 can be recruited to splicing-competent"

Referee #3:

Jobbins and colleges report the colocalization of SRSF1 and U1 snRNP detected by single molecule total internal reflection fluorescence microscopy. SRSF1 is an important splicing factor, that is essential and linked to cancer, thus understanding its biological role and underlying mechanisms is very relevant. They first show that the colocalization of SRSF1 from a nuclear extract and an exogenous fluorescent pre-mRNA is dependent on the ability of U1 snRNP to recognize the 5'SS, that is independent of the presence of exonic splicing enhancer sequences (ESE) but additive. Then the authors claim that this interaction may be important for exon definition due to an observed increased on colocalization of the 3' SS proteins with a fluorescent pre-mRNA when there is a downstream 5´SS, being a similar effect as the one obtained in the presence of ESE sequences. The authors test whether deltaSRSF1 (without the RS domain) is able to interact with a reconstituted U1 snRNP and find that it interacts with the SL3 of the U1 snRNA, as has been previously proposed based on eCLIP experiments. They provide convincing evidence that deltaSRSF1 indeed binds to SL3 of U1 snRNA in vitro using NMR experiments. Finally, the authors attempt to link their findings with splicing efficiency and potential roles in exon definition.
Some of the results concerning the stoichiometry of SRSF1 and U1 and binding of SRSF1 with U1 have already been reported in the literature by the authors and by the Yeo group. An interesting finding of the current study it the mapping of the SRSF1 - U1 interaction with some (although not sufficient) structural detail. Although the hypothesis and some of the results appear relevant for the understanding of the exon definition mechanism, this conclusion is not well supported by the data provided and should be addressed experimentally, - or the statements regarding this should be toned down. There are also some technical questions that the authors should address.
Major points:
– In the first part (page 6-8), the authors examine the colocalization of SRSF1 and the labelled RNA and found that 20-30% of colocalization using the standard GloC pre-mRNA. After duplication of the sequence (i.e. two intron) they observed a duplication of the colocalization (also a duplication of the distribution of SRSF1 molecules respect to one molecule of pre-mRNA) and after disruption of the snRNP U1 binding they observed a reduction on the % of colocalization (by about half). Then they also labelled a U1 snRNP component U1A and observed also a colocalization with the labelled pre-mRNA (30%) and a triple colocalization (pre-mRNA + SRSF1 + U1A). The authors take into account the presence of endogenous unlabelled proteins in the distribution of the bleaching events. But when they examine the colocalization of U1 and SRSF1 in the absence of exogenous labelled pre-mRNA (pag 8-9) they find that there is also some colocalization between both proteins (SRSF1 and U1A). The authors claim that this is happening in the absence of pre-mRNA. According to the experimental procedures they do not remove the endogenous mRNAs during the nuclear extract preparation so this colocalization cannot be ruled out to happen via binding to the present pre-mRNAs in the nuclear cell extract.
– The authors claim that the addition of a consensus 5´SS at the end of the GloC and BGSMN2 pre-mRNAs increases the splicing (page 10) but they do not show the data. Also they test if the pre-mRNA extension enhances the colocalization of 3´SS proteins (U2AF35, U2AF65 and U2B') (suppl figure 5) and compare with their previous published data (Jobbins et al, 2018). As they have shown before, the relative amount of fluorescently labelled vs endogenous protein could cause some differences in the % of colocalization (NE1 has 90:10 ratio and a 31% of colocalization of EGFP_SRSF1 while NE2 has 59:41 ratio of labelled protein vs endogenous and a smaller % of colocalization - 21%), so the question is if they used the same batch of nuclear extract for the experiments with the unmodified GloC pre-mRNA, with the 4xESE variant and with the one they are testing now

(they say in the text that the results are contemporaneous - at similar time, but similar extract?. If not the differences that they observe can arise from the use of nuclear extracts with different labelling ratios).
– In the NMR titrations (page 11), the authors claim that the CSP saturates at a 1:1 ratio but only show one point of the titration. Also they do no further characterize the affinity of this interaction and compare it between the SL3 and the ssRNA. This should be provided.
– Another question concerns the structural basis of this interaction. Specifically, do the authors believe that the SL3 is melted upon binding as they claim due to binding to the GGA motif, which forms a duplex in the SL? This is an important point that needs to be addressed, preferably by providing a three-dimensional structure of the complex, but at least by providing evidence about the conformation of the RNA that is recognized.

Minor points:
– Two citations appear in the text but in press, I can find one but not the other. The unpublished reference should be provided for the review of the current manuscript.
– In some of the graphs (Fig2C, Fig3B) there are some incorrect labels, probably pasted by mistake.
– In some parts of the text the U2 associated factors recognizing the 3´SS are named as U2AF1/2 (e.i. page 3) and in others U2AF35/65 (e.i. page). It is recommended to use the 1/2 nomenclature but in any case it should be consistent along the text.

Access to supporting data: https://figshare.com/xxxx

Responses to reviewers

To begin, we would like to thank the reviewers for the work involved in their analyses of this manuscript and for their helpful and constructive comments. These have, we hope, resulted in very significant improvements to the manuscript. The reviewers' comments are in blue, and our responses are in black.

Reviewer 1

In this manuscript, the authors probe the binding targets of the splicing enhancer SRSF1. Using quantitative single-molecule imaging, the authors uncover a new mode of SRSF1 binding in which the protein targets stem loop 3 on the U1 snRNP. Potentially, this observation is an impactful conceptual advancement in our understanding of spliceosome assembly. A new issue is raised however: if U1 is capable of independent recruitment of SRSF1, how does one achieve exon definition? A few comments need to be addressed before the manuscript can be published.

Major comments:

(1) Compared to the established binding mode in which SRSF1 binds exonic sequences independently of the U1 snRNP, how strongly does SRSF1 bind stem loop 3 of U1? The single molecule stoichiometry experiments indirectly suggest that the binding affinities of these two distinct substrates could be quite similar however quantitative measurements of binding affinity are missing from this manuscript. The authors should perform in vitro binding assays to quantify the relative binding affinity of SRSF1ΔRS to the U1 snRNP (or just stem loop 3) compared to a typical exonic binding site for this protein. No matter the outcome, this result would provide insight into the relative prevalence of these binding modes. Furthermore, in vitro binding assays should also be performed to the stem loop mutant used in figure 4 C to show that this mutation really does prevent SRSF1 binding.

In order to quantitively access the strength of the binding of SRSF1ΔRS to stem loop 3, we performed isothermal titration calorimetry (ITC). The protein was gradually added to the diluted solution of SL3 at 27°C. We determined a dissociation constant of 10.9 ± 2.8 µM (see Figure 1 below). The binding appears to be roughly 200-times weaker than for an optimal ssRNA motif containing a GGA motif preceded by a CA motif (Clery A *et al.*, Nat. Comm. 2021). In the case of SL3, the CA motif is located in the loop, in a ssRNA region. The solution structure of SRSF1 RRM2 bound to RNA clearly shows that the pseudoRRM is a specific ssRNA binder (Clery A et al., PNAS 2013). However, the GGA motif is involved in base-pairing at the apical part of the stem and the binding of SRSF1ΔRS to stem loop 3 will compete with the formation of the secondary structure, thus explaining the reduced affinity of SRSF1ΔRS for stem loop 3 when compared to ssRNA. As suggested by the reviewer, we also performed the same experiment by replacing the wild-type SL3 by the SL3 mutant. Using ITC, we only observed residual binding and the strength of the binding cannot be quantitatively determined. The removal of the CA and GGA motif of SL3 impaired the binding of SRSF1ΔRS on U1 snRNA stem loop 3 (Figure 1 below). These data have now been added as Appendix Fig. S9. Note that we have previously seen in the case of the splicing factor FUS that micromolar affinities for SL3 have physiological roles. However, we cannot rule out that other proteins or enzymatic activities (as helicases) would favour the binding of SRSF1 on U1 SL3 *in vivo* or in nuclear extracts or that the RS domain will play a role in this protein-RNA interaction.

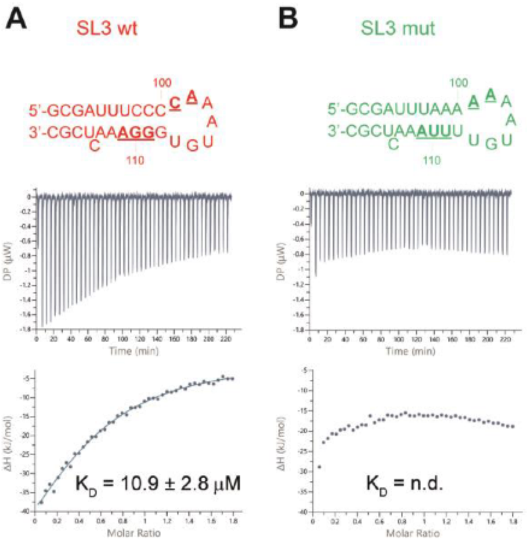

Figure 1 – SRSF1ΔRS binds specifically to U1 snRNA stem loop 3. *(A) ITC titration curve showing the binding of SRSF1ΔRS on U1 snRNA stem loop 3 at 27°C. The dissociation constant of the interaction was 10.9 ± 2.8 µM. (B) ITC titration curve showing the binding of SRSF1ΔRS on U1 snRNA stem loop 3 mutant at 27°C. The binding curve could not be fitted. This is included in the manuscript as part of Appendix Fig. S9.*

(2) In figure 4C the authors argue that that efficient splicing requires SRSF1 in complex with stem loop 3 of the U1 snRNP. However, the authors should address (1) the impact of this mutation on the binding of other proteins, namely FUS and PTBP1 which appear to target stem loop 3 as well and (2) the impact of this mutation on U1 structure and its implications for spliceosome assembly. As the manuscript stands, figure 4C does not convincingly measure the contribution of SRSF1 to the overall reduction in splicing observed in condition 5. Therefore, this manuscript would benefit from a more convincing link between SRSF1/U1 heterodimerization and splicing efficiency.

As shown above, the mutations on SL3 reduce the binding of SRSF1 *in vitro* which explains the reduced splicing efficiency of SMN1 exon7. The reviewer suggested that the reduced splicing efficiency could also be due to the U1 snRNP ability to recruit other splicing factors as FUS or PTBP1. Indeed, we recently shown that FUS interacts with U1 snRNP through direct protein-RNA interactions involving the stem loop 3 and solved the solution structure of the FUS RRM bound to U1 snRNA stem loop 3 (Jutzi, Campagne, *et al.*, Nat. Comm. 2020). The structure showed that FUS binds to the YNY motif (UGU) located at the 3'-part of the loop and uses the α1-β2 loop to contact the loop- adjacent major groove while the C-terminal part of the protein interacts with the loop- adjacent minor groove. In order to address this point, we performed a NMR titration of the FUS RRM with either SL3 or SL3 mutant. As expected, similar binding properties of FUS for SL3 or SL3 mutant were observed (Figure 2A-B), suggesting that the decrease in splicing efficiency is not due to FUS. The reviewer suggested that we also try with PTBP1 due to the presence of CLIP-induced deletions on SL3 on the heat map shown in Appendix Fig. S10. However, we did not investigate further this interaction since we know neither the region of PTBP1 that contacts SL3 nor the functional relevance of this interaction.

The reviewer also questioned the impact of the SL3 mutations on the tertiary structure of SL3 and its potential impact on spliceosome assembly. The stem loop 3 mutations were designed in order to remove the potential binding site for SRSF1 and preserve the base pairing and the secondary structure of the RNA. According to mfold predictions, the stem loop 3 base pairing is preserved by the mutations and it should not affect U1 snRNP structure. In addition, our U1 suppressor assay clearly showed that the SMN1 exon 7 splicing is not fully abolished with the SL3 mutations but rather diminished, in line with residual ability of U1 snRNP to induce splicing. This means that SL3 mutations preserve U1 snRNP biogenesis. In addition, subtle mutations of SL3, C101A or C99G/G109C that abolished the binding site for SRSF1 RRM1 or RRM2, respectively, only partially affect the splicing of SMN1 exon 7 in our U1 suppressor assays (See Figure 2C).

These experiments were performed only twice and are not statistically relevant to be included in the manuscript but they represent strong arguments against a total loss of U1 snRNA structure upon U1 snRNA SL3 mutations.

In summary, these experiments support the conclusions we drew initially from the U1 rescue assays. These results are shown in Appendix Fig. S9.

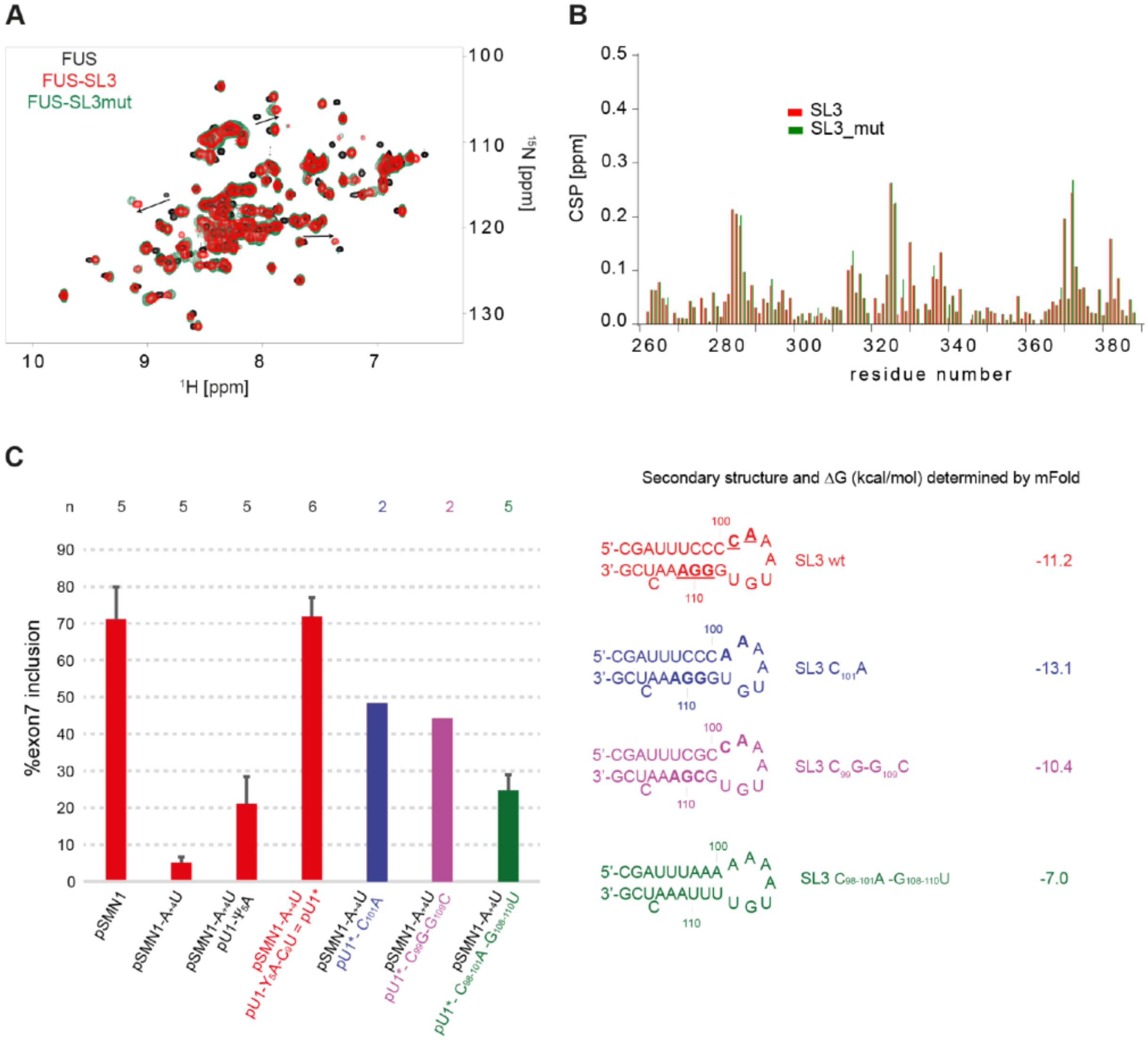

Figure 2 – Mutations of SL3 do not perturb FUS binding to SL3 in vitro and preserve the tertiary structure of the U1 snRNA. *(A) Overlay of the of the 2D ^{15}N-^{1}H HSQC spectra of ^{15}N-labelled FUS RRM before (black) and after addition of 1.33 molar equivalents of SL3 wild-type (red) or SL3 mutant (green). (B) Plot of the normalized chemical shift perturbations (CSP) of the FUS amide resonances upon titration with SL3 wild-type (red) or SL3 mutant (green). (C) Results of the U1 suppressor assay showing intermediate mutations. On the left, bar plot showing how the SMN1 exon 7 splicing event became independent of the endogenous U1 and was rescued by the exogeneous U1 snRNA expression. Mutations C101A or C99G/G109C, that respectively disrupt SRSF1 RRM1 or RRM2 binding site without affecting the secondary structure of SL3 showed moderated effects on the SMN1 exon 7 splicing. These effects are additives as shown by the results obtained*

with the mutant C98-101A/G108-110U. On the right, the SL3 sequences are depicted as well as their theorical relative stability (deltaG, computed with mFold).

(3) The section titled, "The association of U1 and SRSF1 does not require pre-mRNA" requires further clarification. In this experiment, why is it not possible that U1 and SRSF1 are in complex with unlabeled endogenous mRNA from the nuclear extract?

We had tested the effects of ribonuclease treatment, and we have included the result in Appendix Fig. S5 and main text line2 220-222. This is a conventional technique used to demonstrate RNA independence. The result showed a small decrease in colocalization (Figure 3 below). While the majority of the colocalization is preserved, the method is not really very convincing in the absence of evidence (not usually provided by authors) that the endogenous RNA has been removed or that the U1 snRNA has not been damaged. In view of the later structural and cross-linking data, proving a direct interaction, we did not feel that the extra investigations were justified. Ellis *et al.* (J. Cell Biol. 181, 921-34; 2008) also concluded that the association of U1-70K with SRSF1 in cells was independent of RNA. In this case, they treated co-immunoprecipitations with ribonuclease and also showed that FRET signals were unaffected by inhibition of transcription.

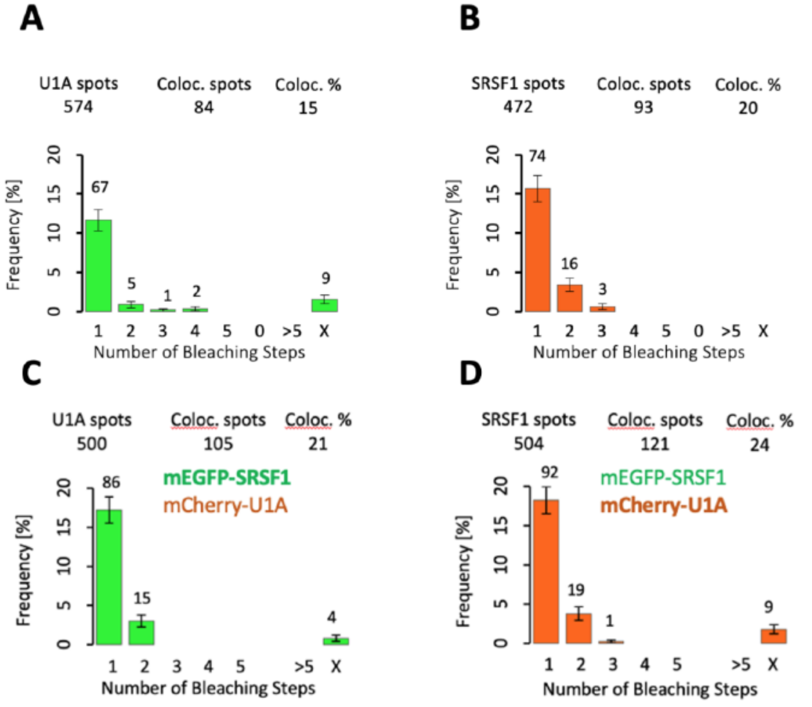

Figure 3 – Frequency histograms showing the numbers of molecules of mEGFP-SRSF1 associated with mCherry-U1A (A, C) and vice-versa (B,D) with (A,B) or without (C,D) treatment with ribonucleases A and T1. *The proteins were co-expressed to levels similar to those of the endogenous proteins in HeLa cells, and nuclear extracts were prepared. The extracts were incubated in splicing conditions, without added RNA, and visualized by TIRF microscopy after dilution and application to a glass cover slip. The Coloc. % is derived from the number of reference spots (mCherry-U1A in the case of panel A) and the number of those that were colocalized (with mEGFP-SRSF1 in the case of panel A).*

Minor comments:

(1) Figure 1A: While the authors performed a rigorous analysis of the number of photobleaching steps for SRSF1-mEGFP and U1-mCherry they also need to perform this same analysis for Cy5 mRNA molecules. I believe the authors only assume that each that Cy5 spot corresponds to only a single mRNA, however this may not be the case and could significantly change the interpretation of the data.

This is a very good point. We examined the data for GloC-U1 (Figure 3B, upper part) and inserted the following in the Methods, (line 784 *et seq.*):
'An alternative explanation for the binding of two molecules of mEGFP-SRSF1 might be that pre-mRNA substrates with ESEs or an additional U1 snRNP binding site might be more prone to dimerization. Analysis of the data for GloC-U1 in Figure 3B showed that about 10% of the Cy5 spots contained two molecules of pre-mRNA. If these were excluded from the analysis the results were the same (by a chi-square test, $p_{inc = exc}$ = 0.91), and the possibility was not taken into account further.'

Furthermore, the authors illustrate their photobleaching analysis workflow with two representative traces. It is preferable to include a figure that illustrates the overall quality of all their traces. This can be done with a histogram that shows the distribution of intensities for all analyzed spots (Cy5, mEGFP, and mCherry). One would expect to see multiple peaks corresponding to different binding stoichiometries. Another potential option is to use transition density plots (https://github.com/ebfret/TransitionDensityPlots).

This is another good question. Plotting the intensities is not helpful, because they vary very widely between molecules and with time. Unlike small dye molecules, single fluorescent proteins emit with a very wide range of intensities for which there is no predictable distribution. Moreover, the intensities of many molecules fluctuate widely with time, due to blinking or perhaps slow movements within the surface-immobilized complexes. However, there is a predictable distribution of photons absorbed prior to bleaching, since each photon absorbed is associated with an independent probability of bleaching, and a good proxy for measuring the total number of photons absorbed is a measurement of the total count of emitted photons (after subtraction of the background). The integrated photon counts should be twice as high, on average, if there were two fluorescent proteins rather than one in a spot. We have inserted Appendix Figure S11 (below) and the following text (lines 622-633 et seq.):

'The validity of the assignment of the step numbers to the traces was checked for an experiment in which a substantial number of RNA molecules were deemed to be associated with two molecules of mEGFP-SRSF1 (Fig. 3B, GloC-U1) by measuring the total number of photon counts emitted by mEGFP for each complex, and comparing the results from complexes assigned as containing one or two molecules of mEGFP-SRSF1 (Appendix Fig. S11). The median number of photon counts emitted from complexes containing one molecule of mEGFP-SRSF1 was 1,472, whereas the median for complexes containing two was 3,085. The distribution between the two classes was significantly different by a 2-tailed Mann-Whitney test (SPSS); $p_{1=2}$ = <0.001. As a check, the values from the complexes containing one molecule were added to the same values rearranged in a random order. The pseudo-double complexes had a median of 3,802, and the distribution was not significantly different from that of the *bona fide* complexes assigned as having two molecules ($p_{2=(1+1)}$ = 0.2).'

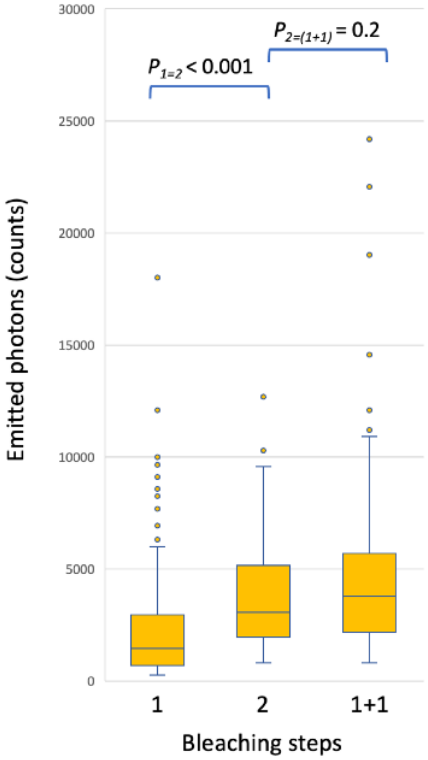

Figure 4 - Complexes identified by bleaching as containing two molecules of mEGFP-SRSF1 emit approximately twice as many photons as those identified as containing one molecule. *The total counts emitted upon irradiation at 488 nm was measured for every spot counted in Figure 3B (mEGFP-SRSF1 colocalized with pre-mRNA GloC-U1). The background measured in the same area after bleaching was subtracted. The counts detected from individual complexes containing one or two molecules of mEGFP-SRSF1 are plotted as a box plots, labelled 1 or 2 respectively. In the plot labelled 1+1, values from the single molecules complexes in plot 1 were shuffled at random and added pairwise to the values in plot 1, simulating complexes with two molecules of SRSF1. The median values are shown as a line in each box, and the values are (1) 1,472, (2) 3,085 and (1+1) 3,802.*

(2) Figure 1A: The images look strangely thresholded. The authors should include what (if any) image filtering they used.

The difference between the background and the spot intensities varies over a wide range, which is represented using just a few intensity levels (to prevent a bright spot hiding other spots). The display has absolutely no influence on the analysis, which is explained in the methods.

(3) The figure legends right now are too minimal. This is very apparent in figure 4 which, in many ways is similar to figure 1 in Roca et al. (PMID: 22588721). The figure legend should have a similar amount of description as this publication to improve readability.

We have expanded the figure legends substantially, especially the previous Fig. 4 (now 5). We hope that they are better.

(4) A number of the figures (2C, 3B) appear to be from screenshots. Remove the artifacts. Figure 3B (top right) also has some formatting issues.

Thank you for pointing this out. We have corrected these errors.

(5) Old and new nomenclature are occasionally used interchangeably (U2AF2 / U2AF65).

We have adopted U2AF65 throughout, for the sake of compatibility with the older references cited.

Reviewer 2

In this work Jobbins and colleagues use a single molecule approach to study U1 complexes deposited on RNAs and find that frequently they colocalize with the SRSF1 factor--ultimately suggesting that SRSF1 can be a constitutive component of the snRNP. They complement these assays with some functional assays, biophysical studies of a potential SRSF1/U1 snRNA interaction, and analysis of eCLIP data. The manuscript data are robust for supporting the central conclusions.

Significantly, the authors provide good evidence for the existence of a "holo"-U1 snRNP that minimally contains SRSF1 in addition to the canonical members. This suggests that canonical U1 may really just be the apo-snRNP and only part of the splicing reaction when in complex with other members. This is a very important addition to the field and will be of interest to a large number of readers interested in splicing mechanism and regulation. In addition, the paper represents a technical tour-de-force and enviable synthesis of multiple experimental methods (single molecule, NMR, CLIP-Seq, in vitro biochemistry and molecular biology) to arrive at their final model. At this stage, however, there are a number of issues which need to be addressed and clarified before publication. Nonetheless, this work is of high interest and the science compelling.

Major Issues

1. Overall the data presentation is challenging. I think the authors may want to consider how that contributes to the overall readability of the manuscript. There were also a number of key experiments in the supplemental that may be better in the main text. I particularly found the result showing that the RS domain wasn't responsible solely for the RNA colocalization was interesting (Figure S6).

The data presentation is a very important point that we considered at length. We have attempted to improve this in figures 2 and 3 by including cartoons that represented our inferences form the data shown. We hope that this will make the manuscript easier to understand. The deltaRS result is interesting, but at this stage it is tempting to infer too much from it (beyond the conclusion we have already given). We are planning additional experiments that would allow us to address the function of the RS domain in future work.

2. I think the data and resulting model are over-interpreted. While the author's provide compelling evidence for a U1-SRSF1 complex, they have no data that specifically excludes other mechanisms such as that in 5A. I think the authors should conclude that their mechanism is an alternate pathway by which U1 can be recruited and the predominant pathway that may occur within a cell or on a particular transcript needs further study. Similarly on line 436, this seems a vast overstatement. I would suggest "U1 snRNP might not play a direct role in all splicing reactions but could also act..."

We apologise for any confusion. It was not our intention to exclude the model in Fig. 5A; there is a great deal of support for it, including some of our own work. Instead, the purpose of the diagrams is to compare the activities of an ESE and a 5'SS in the recruitment not of U1 but of SRSF1. We have modified the former line 436 (now line 577) as the reviewer suggested. Incidentally, as we noted in the following section of the discussion, the possibility that SRSF1 acts downstream of SRSF1 is given weight by the finding of SRSF1 in the cryo-EM structure of a pre-Bact complex, at a stage subsequent to U1 dissociation (Townsend *et al.*, 2020).

3. The experimental setup described on page 5 is inadequate. First, how was functional monomeric SRSF1 confirmed? I'm assuming this is based on the data in S1A, however this isn't clearly described and it is unclear if this is an in vivo or in vitro assay.

We apologise again for the confusion; these experiments were done in cells, which from our perspective here is *in vivo*. The legend to Figure S1 states that the analyses were done by transfection of plasmids into HeLa cells and measurements of the ratio of spliced to unspliced <u>endogenous</u> SRSF1 mRNA by RT-PCR.

Other labs have shown also that the fusion of mEGFP-SRSF1 with mEGFP at the N-terminal side is functional, and these are cited in the Results (line 119).

Why do extracts contain different amounts of mGFP-SRSF1?

This is because each extract is made from cells transiently transfected with plasmids expressing mEGFP-SRSF1. The efficiency of transfection varies from experiment to experiment.

Does the amount of mEGFP-SRSF1 present correlate with the amount of spliced/unspliced using the assay in Figure S1A?

This was not tested with the cell-based assays.

Were the U1 molecules attached to the pre-mRNAs under conditions that fully support splicing?

Yes. We have added a phrase to confirm that (line 118). As we state in the following lines, splicing itself was blocked by the addition of an oligonucleotide that blocked assembly at complex A.

How was U6 ablation confirmed--by in vitro splicing assays or Northern blotting against the U6 snRNA? In our hands, the amount of U6 oligo needed to ablate the activity is extract dependent.

The referee is presumably thinking of oligonucleotides that direct RNase H cleavage of U6 snRNA. The 2'-O-methyl analogue that we used does not support RNase H cleavage but, as Donmez et al. (2007) showed, it is very effective. We have found that the alternative method, incubation with anacardic acid, does require titration of the reagent but the oligonucleotide is reproducibly effective (see, for example, Hodson *et al.*, 2012).

Finally, were the RNA molecules analyzed single molecules of RNA? Or is it possible that some RNA spots being analyzed were actually clusters of multiple RNAs either containing non-fluorescent RNA molecules or those unable to resolved by the microscope as individual spots?

This is a good question, answered in regard to the number of Cy5-labelled molecules present in our responses to Referee 1, minor comment 1. Inadequate resolution was addressed by maintaining a very sparse distribution of labelled molecules. Each pixel corresponds to ~160 nm on the slide, and a spot is considered to be 3x3 pixels (around or larger than the limit of resolution). Boxes of 19x19 pixels are moved across the image and intensities fitted to 1D Gaussians. If two intensity peaks are found, the box size is reduced until only one peak remains. If the box has to be less than 9x9 pixels, then the spots are automatically rejected. Thus, the probability that two spots are superimposed by chance is negligible, and the probability that two close spots are counted together is also very low. Non-fluorescent RNAs would have to be endogenous. Is the suggestion that what appear to be single molecules of RNA bound by one molecule of U1 or SRSF1 actually comprise multiple RNA molecules, each cluster being associated with one molecule of SRSF1 or U1 that has also bound to endogeneous RNA? In this case, it would be hard to explain why the addition of labelled RNA with two binding sites resulted in an altered distribution of U1 and SRSF1 consistent with binding by by two molecules of U1 or SRSF1. It would also be hard to explain why the pattern of association was so strongly affected by ablation of a 5'SS. It is worth noting too that the RNA-independent association of U1 and SRSF1 remained after RNase treatment (Appendix Figure S5).

4. A major experiment in the paper was showing that association of U1 and SRSF1 can occur without pre-mRNA. However, no details were included about how that experiment was carried out. Is it possible that this interaction occurs mainly in vitro?

The experiment was carried out just like the others but without the addition of labelled RNA. The experiment with ribonuclease is described in the Methods. The interaction of U1 and SRSF1 *in vivo* has been documented previously and shown to be RNA-independent (line 387 *et seq.*). What we show here is that the complex is not an aggregate but has a fixed 1:1 stoichiometry.

What happens if a NE containing only fluroescent U1 (and non fluorescent but over-expressed SRSF1) was combined with a NE containing nonfluorescent U1 and over expressed fluorescent SRSF1? Would colocalization still be observed? If so, that may reveal some information about the relative stabilities and dynamics of the U1/SRSF1 complex.

We have done some preliminary experiments to look at this interesting question. The results suggest that colocalization is not observed at the same level. However, many more experiments would be needed to validate this and to extract useful information from it.

5. Another major experiment is showing that the U1/SRSF1 complexes can recruit U2. This is important since it implies that the U1/SRSF1 complexes being studied are chemically competent for splicing and advancing through the reaction. However, this experiment was very poorly described and this reviewer could not make sense of the data in Table 2. Do U1/SRSF1 complexes also colocalize with U2? If this cannot be measured directly, then this should be noted in the manuscript and the competence of the studied complexes needs to be made less certain.

The point of the experiment was to show that the 5'SS at the 3' end of the exon was able to stimulate the recruitment of U2AF and U2 snRNP, which is a central feature of exon definition and one that is shared with the actions of ESEs. We did not intend to claim that the U1/SRSF1 complexes colocalised with U2, although had we thought this point important we could easily have done the experiment at the time. We have modified the conclusion to this section (line 280) to read, 'We conclude that the 3'-terminal 5'SS mediates increases in the recruitment of single molecules of U2AF and U2 snRNPs equivalent to those produced by four repeats of a strong enhancer, showing that it is functional in the key reaction of exon definition, and that U1 snRNP binding to it recruits a molecule of SRSF1.'

6. Table 1 is a bit confusing. %labeled:unlabeled perhaps should be %FP tagged/endogenous. The critical value for interpretation of the data is the % fluorescent complexes (# fluorescent molecules/total molecules). This is not necessarily the same as the FP tagged percentage since not all FP chromophores will mature. Can the authors correct for the maturation percentage? Does this impact the data interpretation? In addition, some values were listed as "n.d." since the levels were equal to the endogenous protein. It is unclear to me why this would be "n.d." for mEGFP-SRSF1 for NE3 then but not for NE2?

We thank the referee for pointing this out. We have changed the axis label as suggested. We did not correct for maturation percentage, which was discussed at length in the second paragraph in the Statistics section of the Methods. Given that the outcome of guessing the maturation percentage (we explain why it would have been only a guess) would be to elevate the apparent proportion of complexes containing two molecules in those cases where we would predict that they did, we thought it better practice to err upon the side of caution and underestimation.
We have removed the line in the legend to Table 1 giving a reason for not having estimated the absolute concentrations in the extract. The important figures for our experiments are the ratios of tagged:endogenous and the level of apparent dimerization.

7. It seems like the distribution of likely U1-dependent SRSF1 spots in Figure 1B can be obtained by subtracting the dstribution in 1 C with anti-U1 from the distribution in 1B with GloC. Similarly, the key analysis for interpretation of the data in 1B from the RNA showing 2 introns would subtraction of a distribution observed from the same RNA under anti-U1 conditions. Would that still show a sizable number of 2 photobleaching spots? Without the 2 intron control, I'm unsure how to interpret the data from that RNA.

This important point was discussed in the Methods (now lines 702 to 719). The distribution seen in the anti-U1 experiment is not a fixed background that can be subtracted from the distributions of interest. It is likely that it represents unproductive complexes, the proportion of which depends on the circumstances (see Methods) but appears to be very small in normal conditions. Thus, it would not be sensible to simply

subtract the frequencies seen in the anti-U1 experiments, where they are at their highest. This is graphically shown by considering Figure 1C, where the subtraction of the frequencies complexes with 3, 4 or 5 molecules of SRSF1 in the anti-U1 experiment from the frequencies seen in the normal conditions would produce negative frequencies!

8. Could apo and holo U1 (U1 +SRSF1) have different functions in the cell? Perhaps one is primarily involved in splicing and the other telescripting?

This is a very interesting suggestion. There are no data in our paper that would suggest this, though, and it might be thought to be stretching the results beyond reasonable speculation if we were to suggest it here.

9. I think it would be worth surveying the literature on characterization of U1--given that many groups have purified U1 from human cells for decades has there ever been any evidence of co-purification of SRSF1?

There are a number of papers that show co-immunoprecipitation, although the problem always is to gain any insights into the stoichiometry and the proportions bound (i.e., the authors might just be detecting aggregates of sticky proteins or very minor populations). We have cited some good examples and the work on FRET in cells in lines 389-393.

Minor Issues:

1. Abstract line 31, maybe "other" components at the 3'SSs line 35, SRSF1 "can be" recruited by a U1 snRNP

Changed as suggested Line 32).

2. line 151 "was restricted and specific rather than stochastic" I am unsure what this means.

This phrase has been removed.

3. Line 154, I found this paragraph very confusing.

We have tried to find ways to improve this, but we just could not find a better form of words. We apologise for this.

4. Line 259, is work from the Zhao lab on the yeast E complex relevant for thinking about how cross-exon complexes form? (Nature. 2019 Sep;573(7774):375-380. doi: 10.1038/s41586-019-1523-6)

Good point. We have have introduced a sentence about this, and a reference (line 452).

5. Discussion: line 352 "can associate", "SRSF1 can be recruited to splicing-competent"

This sentence has been changed as per the referee's suggestion (line 381).

Reviewer 3

Jobbins and colleges report the colocalization of SRSF1 and U1 snRNP detected by single molecule total internal reflection fluorescence microscopy. SRSF1 is an important splicing factor, that is essential and linked to cancer, thus understanding its biological role and underlying mechanisms is very relevant. They first show that the colocalization of SRSF1 from a nuclear extract and an exogenous fluorescent pre-mRNA is dependent on the ability of U1 snRNP to recognize the 5'SS, that is independent of the presence of exonic splicing enhancer sequences (ESE) but additive. Then the authors claim that this interaction may be important for exon definition due to an observed increased on colocalization of the 3' SS proteins with a fluorescent pre-mRNA when there is a downstream 5´SS, being a similar effect as the one obtained in the presence of ESE sequences. The authors test whether deltaSRSF1 (without the RS domain) is able to interact with a reconstituted U1 snRNP and find that it interacts with the SL3 of the U1 snRNA, as has been previously proposed based on eCLIP experiments. They provide convincing evidence that deltaSRSF1 indeed binds to SL3 of U1 snRNA in vitro using NMR experiments. Finally, the authors attempt to link their findings with splicing efficiency and potential roles in exon definition.

Some of the results concerning the stoichiometry of SRSF1 and U1 and binding of SRSF1 with U1 have already been reported in the literature by the authors and by the Yeo group. An interesting finding of the current study is the mapping of the SRSF1 - U1 interaction with some (although not sufficient) structural detail. Although the hypothesis and some of the results appear relevant for the understanding of the exon definition mechanism, this conclusion is not well supported by the data provided and should be addressed experimentally, - or the statements regarding this should be toned down. There are also some technical questions that the authors should address.

Major points:

− In the first part (page 6-8), the authors examine the colocalization of SRSF1 and the labelled RNA and found that 20-30% of colocalization using the standard GloC pre-mRNA. After duplication of the sequence (i.e. two intron) they observed a duplication of the colocalization (also a duplication of the distribution of SRSF1 molecules respect to one molecule of pre-mRNA) and after disruption of the snRNP U1 binding they observed a reduction on the % of colocalization (by about half). Then they also labelled a U1 snRNP component U1A and observed also a colocalization with the labelled pre-mRNA (30%) and a triple colocalization (pre-mRNA + SRSF1 + U1A). The authors take into account the presence of endogenous unlabelled proteins in the distribution of the bleaching events. But when they examine the colocalization of U1 and SRSF1 in the absence of exogenous labelled pre-mRNA (pag 8-9) they find that there is also some colocalization between both proteins (SRSF1 and U1A). The authors claim that this is happening in the absence of pre-mRNA. According to the experimental procedures they do not remove the endogenous mRNAs during the nuclear extract preparation so this colocalization cannot be ruled out to happen via binding to the present pre-mRNAs in the nuclear cell extract.

We have included the results from an experiment in which RNases A and T1 were added (Appendix Fig. S5). Please see also our response to Referee 1, point 3, which covers this in more detail.

− The authors claim that the addition of a consensus 5´SS at the end of the GloC and BGSMN2 pre-mRNAs increases the splicing (page 10) but they do not show the data.

A splicing gel showing the results for BGSMN2 is shown now in Appendix Fig. S6. Since GloC splices very well anyway, and assembles complex A very rapidly, the improvement is marginal, and we have altered the text to refer only to BGSMN2.

Also they test if the pre-mRNA extension enhances the colocalization of 3´SS proteins (U2AF35, U2AF65 and U2B') (suppl figure 5) and compare with their previous published data (Jobbins et al, 2018). As they have shown before, the relative amount of fluorescently labelled vs endogenous protein could cause some differences in the % of colocalization (NE1 has 90:10 ratio and a 31% of colocalization of EGFP_SRSF1 while

NE2 has 59:41 ratio of labelled protein vs endogenous and a smaller % of colocalization - 21%), so the question is if they used the same batch of nuclear extract for the experiments with the unmodified GloC pre-mRNA, with the 4xESE variant and with the one they are testing now (they say in the text that the results are contemporaneous - at similar time, but similar extract?. If not the differences that they observe can arise from the use of nuclear extracts with different labelling ratios).

This is an important point. The previous text did say that the experiments with all three substrates were done at the same time with the same extract (line 271). However, we agree that the point was buried in the text, so we have added the statement to the legend to Table 2 as well (lines 1396-7).

− In the NMR titrations (page 11), the authors claim that the CSP saturates at a 1:1 ratio but only show one point of the titration. Also they do no further characterize the affinity of this interaction and compare it between the SL3 and the ssRNA. This should be provided.

The binding of SRSF1 on U1 snRNP saturates at a ratio of 1:1, although it is very difficult to determine the concentration of the U1 snRNP solution stock precisely (ε_{260}=220000 $M^{-1}.cm^{-1}$). We have added the intermediate points to the titration but the spectral overlay is very difficult to read (intermediate exchange) and thus, we kept our previous overlay in the current version of the manuscript.

In order to quantitively access the strength of the binding of SRSF1ΔRS to stem loop 3, we performed isothermal titration calorimetry (see above, Figure 1). The protein was gradually added to the diluted solution of RNA. We determined a dissociation constant of 10.9 ± 2.8 µM. The binding appears to be 200-times weaker than for an optimal ssRNA motif containing a GGA motif preceded by a CA motif (Clery A et al., Nat. Comm. 2021). The results are included in the Results in lines 319-321.

− Another question concerns the structural basis of this interaction. Specifically, do the authors believe that the SL3 is melted upon binding as they claim due to binding to the GGA motif, which forms a duplex in the SL? This is an important point that needs to be addressed, preferably by providing a three-dimensional structure of the complex, but at least by providing evidence about the conformation of the RNA that is recognized.

We agree that this is an important point. As mentioned by the reviewer, SRSF1ΔRS was proposed to bind the $C_{101}A_{102}$ motif using its RRM1 and the $G_{109}G_{110}A_{111}$ using its RRM2. However, the GGA motif is embedded in a secondary structure at the apical part of the loop. Using NMR spectroscopy, we found that SRSF1ΔRS experienced the same chemical shift perturbations when titrated with SL3 or its preferred ssRNA target at 40°C. However, when we performed the same experiment at 30°C, saturation of the chemical shifts of SRSF1ΔRS was delayed and required an excess of stem loop 3. This already suggested that the binding of SRSF1ΔRS to SL3 probably competes with the formation of stable base pairs involving the GGA motif.

In order to probe the status of the RNA base pairing during the formation of the complex, we monitored the signals of SL3 imino protons upon addition of SRSF1ΔRS. First, we assigned the imino resonances (U/G) of the free SL3 by recording a 2D ^{1}H-^{1}H imino-selective SOFAST NOESY and a 2D ^{1}H-^{15}N HSQC of the RNA (Figure 5A-B below). Then, we monitored the intensities of the imino signals upon addition of SRSF1ΔRS and observed that U97 and G109 imino signals decrease upon addition of SRSF1ΔRS (Figure 5C). The intensity decrease of the G110 imino signal cannot be observed due to its overlap with the signal of G93. This observation shows that SRSF1ΔRS perturbed the base pairing of SL3. The RNA experienced a conformational change upon binding to SRSF1; the apical part of the stem loop containing the GGA motif melts. Consequently, it would explain why SRSF1ΔRS interacts with a micromolar affinity with SL3 when nanomolar affinities are observed with ssRNA targets. The binding of the GGA motif by SRSF1 RRM2 will be in constant competition with the formation of the stem *in vitro*. However, the situation might be different *in vivo* or in nuclear extracts. Other proteins, such as helicases (Prp5 or UAP56) could facilitate the melting of SL3 and the binding of SRSF1. Furthermore, the RS domain of SRSF1 could also interact with U1 snRNP (with the RS domain of U1-70K or the RNA component) and consequently increase the global affinity of SRSF1 for SL3.

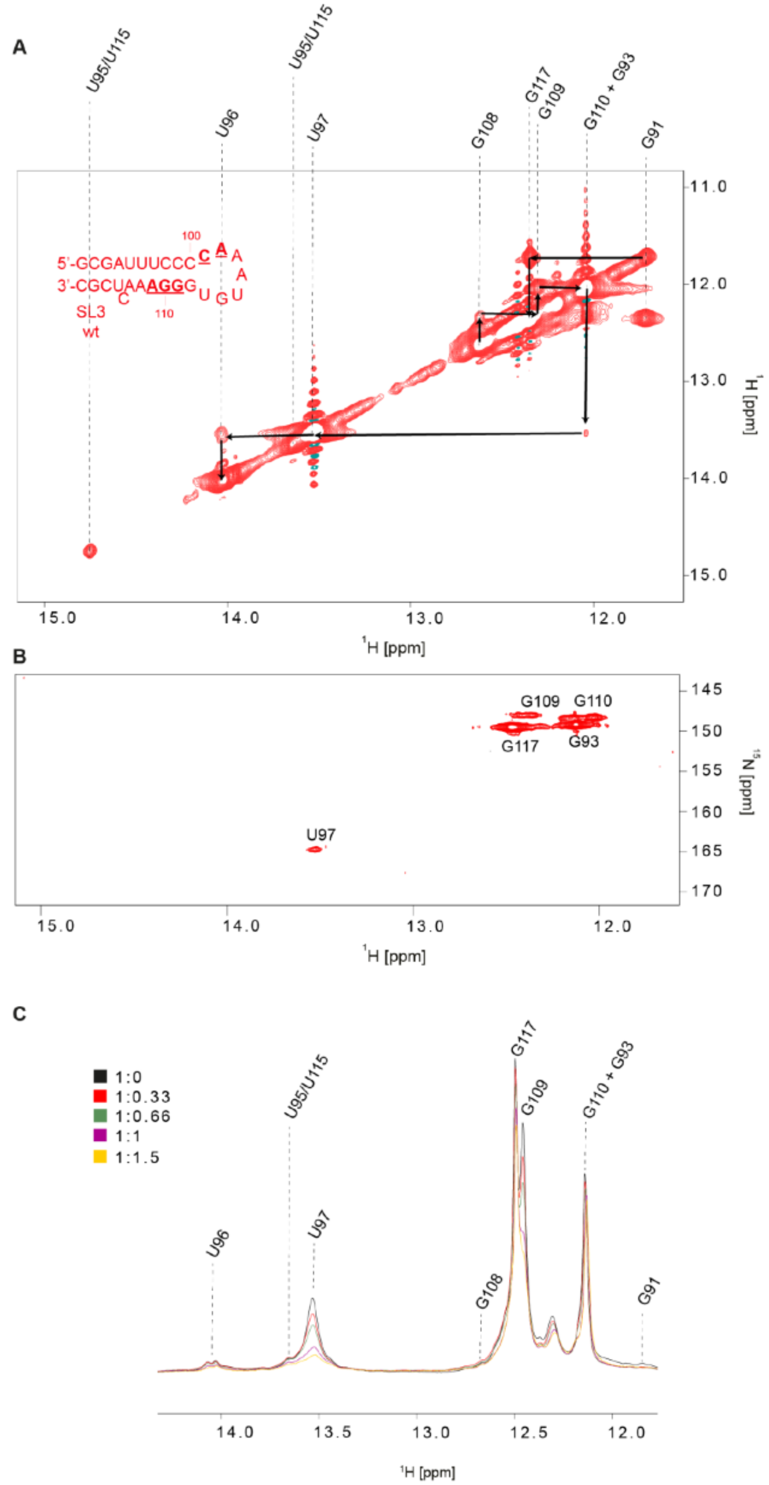

Figure 5 – The binding of SRSF1ΔRS on SL3 perturbs RNA base pairing. *(A) 2D ^{1}H-^{1}H SOFAST NOESY of SL3. The connection between neighbouring iminos are depicted. Since no cross-peaks were observed for two uracil iminos, they have double labels corresponding to both possibilities and could corresponds to U95 and U115 located on the base pairs surrounding the unpaired C114. (B) 2D ^{1}H-^{15}N HSQC of ^{13}C-^{15}N SL3. (C) Overlay of the 1D ^{1}H SOFAST imino recorded upon successive additions of SRSF1ΔRS. The spectra are colored according to the ratio SL3: SRSF1ΔRS. All the data were recorded at 313K in the NMR buffer (NaPO4 10 mM pH7.0, L-Arg 50 mM, L-Glu 50 mM, DTT 2mM). The data in C are shown in Fig. 5C in the revised manuscript.*

Minor points:

– Two citations appear in the text but in press, I can find one but not the other. The unpublished reference should be provided for the review of the current manuscript.

Thank you; corrected.

– In some of the graphs (Fig2C, Fig3B) there are some incorrect labels, probably pasted by mistake.

Also corrected; thank you.

– In some parts of the text the U2 associated factors recognizing the 3´SS are named as U2AF1/2 (e.i. page 3) and in others U2AF35/65 (e.i. page). It is recommended to use the 1/2 nomenclature but in any case it should be consistent along the text.

We corrected these to U2AF35/65, for the sake of cross-reference with important older papers.

1st Revision - Editorial Decision 9th Sep 2021

Dear Prof. Eperon,

Thank you for submitting your revised manuscript. Please also excuse the delay in communicating this decision to you, which was due to delayed referee responses over the summer, as well as further discussions regarding the issues raised by one of the referees. Please find the comments of the three original referees below.

As you will see, referee #2 and referee #3 still express a number of concerns. I have consulted with all referees on referee #3's issues regarding the availability of experimental data to support main experiments and their analysis (ref #3- point 1, 2, 3), in particular the co-localization. We recognize that you have provided a large amount of source data, which includes imaging series. However, we would ask you to further address this issue:

1) To make the uploaded datasets easier to navigate for the reader, please add a "read-me" document detailing what the files represent to the respective folder (i.e. n stacks of x images acquired by y and analyzed by z).
2) Please also carefully review the Materials and Manuscript section on data acquisition and data analysis, and ensure that all necessary information is provided. In particular, it is important to make the following points clear, also to non-experts:
a) What was the measure to distinguish between spots that were co-localized or not?
b) Which controls were used to ensure mapping between images collected at different wavelengths is correct?
c) How was co-localization defined? (less than or equal to 2 pixels?)
3) Please also consider revising the figure (also with respect to referee #2's comments) and adding example images for co-localization or no co-localization.

In addition to these issues, please also address referee #2's points regarding the clarity of the respective figures and revise the figure legends or if needed the figure. These changes will also overall improve the accessibility of your findings to readers that are not directly working in this field. When submitting the revised version, please also include a point-by-point response to all of the referees' comments.

As mentioned in the previous decision letter, it is normally EMBO Journal's policy to allow only one round of major revision, such that it is now crucial that you address the remaining referee concerns fully in the next revised version. If you have any questions regarding this revision or would like to discuss any points in more detail, please contact me.

Kind regards,

Stefanie

Stefanie Boehm
Editor
The EMBO Journal

****

Referee #1:

Overall, the authors made a serious attempt to answer my questions.

1) I appreciate how they measured SRSF1's affinity for stem loop 3 and the stem loop 3 mutant even though the result is rather disappointing and, in my opinion, puts the overall significance of this finding into question. It also seems like they're comparing their measured KD to a previously published value for SRSF1 binding to its exonic site (Clery A et al., Nat. Comm. 2021) instead of doing the measurement themselves with the same buffer conditions and same assay. Nevertheless, I am satisfied that they performed these binding assays.

2) I also appreciate how they performed additional NMR experiments to probe whether the SL3 mutant impacts FUS binding (none observed). While it would've been nice to test if this mutant impacts PTBP1 binding, I understand this this could be difficult and perhaps beyond the scope of what is reasonable.

3) To address our concerns about endogenous RNAs present in their photobleaching assay, they directed us to the supplement where they added a nuclease and still measured colocalization between SRSF1 and the U1 snRNP. They also added a sentence to the main text. As they point out, this isn't the right assay to do here as there is the potential to degrade the U1 snRNA. Because of this issue, my opinion is that the following claim on line 228 is not well supported with the data presented at this point in the manuscript: "Our results show that a significant proportion of each protein is present in a heterodimer in functional splicing conditions." I suggest moving this section to later in the manuscript, after the NMR and splicing assays.

4) I'm fine with all of their comments to our minor issues.

Referee #2:

In this revision, the authors have thoroughly and thoughtfully responded to the issues raised by the reviewers. The scientific conclusions are sound and caveats/issues for future consideration are well described. Moreover, the experiments have now been better placed in a broader context for the field. Overall, this is an excellent paper with significant impact for the field and I fully support publication in EMBO J.

That being said an important remaining issue is the clarity of the figures and their presentation. This really detracts from the science and conclusions. For example, Figure 1A is now a hodge-lodge of video images, cartoons, raw and fitted fluorescence intensity traces, and histograms. Even after reading the legend it is unclear what is what and the logical flow of the experiment. I assume the green circle in the cartoon in 1 A is mEGFP SRSF1 but why is that not labeled?How will color blind readers be able to interpret this or other figures? I think the authors should make figure 1 A its own figures where the raw data, integrated fluorescence traces and fits, and histograms can all be logically and orderly presented and with their own figure labels (1 A, 1 B, 1 C, etc ..).
Similarly, many figures have multiple parts that are not uniquely labeled. Figure 1 B has 4 sections: 2 histograms and 2 cartoons. In Figure 1 C, the label on U1 is white instead of black since presumably U1 was unlabeled in this experiment but where is this explained? Why does only the first histogram in 1 C get a cartoon and not the others? Do the cartoons always represent the interpretation of the experimental results or are they meant to represent the assay? In 1 B, why are the green circles not associated with the RNAs? Are they not bound? Why is U1 no longer labeled in Figure 2? What does Grey vs. orange U1 represent? Why are some cartoons to the left of histograms, some to the right, and others in the middle to be shared by two histograms?
This is really a major issue with Figures 1-3. While I don't think any changes are absolutely required for acceptance given the strength of the data and the significance of the science, the confusion these figures will generate will greatly reduce the readability and impact of the manuscript.

Referee #3:

In the revised manuscript, the authors responded to the points raised, and provided some additional experimental support, especially for the in vitro results. In many cases however, the authors merely restate what was written in the original version of the manuscript, with some further explanations, but without providing additional experimental support, or showing raw data, i.e .concerning the colocalization results. In my view some of these points should still be improved.

- The authors merely provide bar plots with the colocalization frequency but no raw data are shown to document the experimental evidence. The authors argue with p-values to support statistical significance, but these state just this, i.e. statistical relevance, and do not necessarily confirm the validity of the conclusions.
- Concerning the lysate used to observe colocalization with U2AF proteins, the authors state that they used exactly the same lysate as in the published work from 2018, but experiments were done with the three substrates, thus the lysate has to be a different one in each case, due to the expression of labelled U2AF proteins. The response is not very convincing and there remains some question whether the conclusions made based on the colocalization analyses are justified, and well supported by the experimental data.

- One strong point of the paper is the binding of SRSF1 to U1snRNP in vitro to support the colocalization data. But now it turns out that this interaction is 200 times weaker than for the ESE sequences and no other evidence of helicases or SR region involvement is shown (this is just speculated about), which could rationalize this. All the data related to the binding are a bit unclear, with different temperatures used, saturation at 1:1 from the protein perspective that is not mirrored in saturation at 1:1 when looking at the SL3 RNA data.

- To provide some support for the proposed melting of SL3 to enable recognition of the GGA motif the authors analyze NMR spectra arguing that the line-broadening observed is consistent with a conformational change, i.e. melting, of the upper part of the stem-loop. This is a reasonable explanation (but line broadening could also be a result of the binding and the increased molecular weight of the complex). It still leaves the result that the overall affinity to SL3 is much reduced compared to the ssRNA and in fact rather low. Will this be relevant in a cellular context to explain the biological effects?

- The authors were asked to document the effect of adding a consensus 5' splice site at the end of GloC and BGSMN2. Data are shown now in Appendix Fig. S6 for BGSMN2, while data for GloC are not shown. These data show only a marginal improvement in splicing (one of two cases) and the text has been altered to acknowledge this. This is fine but does not really strengthen the overall conclusions of the manuscript.

- Nomenclature should be changed to U2AF2/1, and rather point to the old nomenclature when first introducing U2AF.

We would like once again to thank the reviewers for their patience and hard work, and we hope that they will be pleased with the revised manuscript. The reviewers' comments are in blue, and our responses are in black.

Reviewer 1

Overall, the authors made a serious attempt to answer my questions.

1) I appreciate how they measured SRSF1's affinity for stem loop 3 and the stem loop 3 mutant even though the result is rather disappointing and, in my opinion, puts the overall significance of this finding into question. It also seems like they're comparing their measured KD to a previously published value for SRSF1 binding to its exonic site (Clery A et al., Nat. Comm. 2021) instead of doing the measurement themselves with the same buffer conditions and same assay. Nevertheless, I am satisfied that they performed these binding assays.

2) I also appreciate how they performed additional NMR experiments to probe whether the SL3 mutant impacts FUS binding (none observed). While it would've been nice to test if this mutant impacts PTBP1 binding, I understand this this could be difficult and perhaps beyond the scope of what is reasonable.

3) To address our concerns about endogenous RNAs present in their photobleaching assay, they directed us to the supplement where they added a nuclease and still measured colocalization between SRSF1 and the U1 snRNP. They also added a sentence to the main text. As they point out, this isn't the right assay to do here as there is the potential to degrade the U1 snRNA. Because of this issue, my opinion is that the following claim on line 228 is not well supported with the data presented at this point in the manuscript: "Our results show that a significant proportion of each protein is present in a heterodimer in functional splicing conditions." I suggest moving this section to later in the manuscript, after the NMR and splicing assays.

We tried to re-position the text as suggested, but it did not seem to flow well. Instead, we moved the sentence in question so that it followed the references to previous work from others that had shown direct and RNAse-resistant interactions, and we modified it to state that our results are consistent with the possibility that, etc..

4) I'm fine with all of their comments to our minor issues.

Reviewer 2

In this revision, the authors have thoroughly and thoughtfully responded to the issues raised by the reviewers. The scientific conclusions are sound and caveats/issues for future consideration are well described. Moreover, the experiments have now been better placed in a broader context for the field. Overall, this is an excellent paper with significant impact for the field and I fully support publication in EMBO J.

That being said an important remaining issue is the clarity of the figures and their presentation. This really detracts from the science and conclusions. For example, Figure 1A is now a hodge-lodge of video images, cartoons, raw and fitted fluorescence intensity traces, and histograms. Even after reading the legend it is unclear what is what and the logical flow of the experiment. I assume the green circle in the cartoon in 1A is mEGFP SRSF1 but why is that not labeled?How will color blind readers be able to interpret this or other figures? I think the authors should make figure 1A its own figures where the raw data, integrated fluorescence traces and fits, and histograms can all be logically and orderly presented and with their own figure labels (1A, 1B, 1C, etc..).

Similarly, many figures have multiple parts that are not uniquely labeled. Figure 1B has 4 sections: 2 histograms and 2 cartoons. In Figure 1C, the label on U1 is white instead of black since presumably U1 was unlabeled in this experiment but where is this explained? Why does only the first histogram in 1C get a cartoon and not the others? Do the cartoons always represent the interpretation of the experimental results or are they meant to represent the assay? In 1B, why are the green circles not associated with the RNAs? Are they not bound? Why is U1 no longer labeled in Figure 2? What does Grey vs. orange U1 represent? Why are some cartoons to the left of histograms, some to the right, and others in the middle to be shared by two histograms?

This is really a major issue with Figures 1-3. While I don't think any changes are absolutely required for acceptance given the strength of the data and the significance of the science, the confusion these figures will generate will greatly reduce the readability and impact of the manuscript.

All the figures have been redrawn, and we have paid close attention to each point that the reviewer made about them. We have, as the reviewer suggested, made a new Figure 1, covering the same ground as Figure 1A but in more detail and with better diagrams. There is an extensive new legend explaining the details of the figure. We hope the reviewer will agree that the manuscript is much more readable and easily understood.

Reviewer 3

In the revised manuscript, the authors responded to the points raised, and provided some additional experimental support, especially for the in vitro results. In many cases however, the authors merely restate what was written in the original version of the manuscript, with some further explanations, but without providing additional experimental support, or showing raw data, i.e .concerning the colocalization results. In my view some of these points should still be improved.

- The authors merely provide bar plots with the colocalization frequency but no raw data are shown to document the experimental evidence. The authors argue with p-values to support statistical significance, but these state just this, i.e. statistical relevance, and do not necessarily confirm the validity of the conclusions.

The raw data were available to the reviewers previously, but we realised that the comments attached to the folders could be made more helpful. Thus, we have amplified the comments to provide much better insight into the contents and significance of the text files that accompany each time series. Each sub-folder contains a time series as a stack of several hundred sequential images obtained using two or three lasers in succession. The accompanying text files describe which lasers were used for which frames. The data is now publicly accessible.

In the main text, we have provided an extra paragraph explaining the calibration procedure used to align images obtained at different wavelengths (lines 610 to 614). The text states (line 616-7) that spots were considered as colocalized if their true centres were separated by up to and including two pixels.

- Concerning the lysate used to observe colocalization with U2AF proteins, the authors state that they used exactly the same lysate as in the published work from 2018, but experiments were done with the three substrates, thus the lysate has to be a different one in each case, due to the expression of labelled U2AF proteins. The response is not very convincing and there remains some question whether the conclusions made based on the colocalization analyses are justified, and well supported by the experimental data.

We are afraid that our description in the Methods must not have been clear. We have altered the Methods on line 528-9 to explain that the lysates, prepared from cells expressing the protein fusions, are aliquotted and stored at -80 deg C. Each experiment involved the subsequent addition to an aliquot of the lysate of whatever pre-mRNA has been transcribed *in vitro* and labelled with Cy5. Thus, it is perfectly possible to use the same lysate for many experiments on separate occasions or, as here, on one occasion with different pre-mRNAs.

- One strong point of the paper is the binding of SRSF1 to U1snRNP in vitro to support the colocalization data. But now it turns out that this interaction is 200 times weaker than for the ESE sequences and no other evidence of helicases or SR region involvement is shown (this is just speculated about), which could rationalize this. All the data related to the binding are a bit unclear, with different temperatures used, saturation at 1:1 from the protein perspective that is not mirrored in saturation at 1:1 when looking at the SL3 RNA data.

As reviewer 3 said, the interaction between SRSF1 and SL3 is 200 times weaker than the interaction between SRSF1 and ESE although they both contains the binding sites for RRM1 and RRM2. However, this is explained by the base pairing observed in the case of SL3. SRSF1 clearly binds to ssRNA (Clery et al., PNAS 2013 and Nature Comm. 2021) and we collected experimental data showing that SL3 has to melt in order to accommodate SRSF1. As mentioned in our manuscript, there was a recent publication suggesting that SL3 is a substrate for the helicase UAP56 (Martelly W et al., RNA biol. 2021).

- To provide some support for the proposed melting of SL3 to enable recognition of the GGA motif the authors analyze NMR spectra arguing that the line-broadening observed is consistent with a conformational change, i.e. melting, of the upper part of the stem-loop. This is a reasonable explanation (but line

broadening could also be a result of the binding and the increased molecular weight of the complex). It still leaves the result that the overall affinity to SL3 is much reduced compared to the ssRNA and in fact rather low. Will this be relevant in a cellular context to explain the biological effects?

This difference in affinity can be explained by the competition between base pair formation and binding of SRSF1. SRSF1 competes with the formation of the secondary structure. In addition, we cannot exclude the possibility that, in a cellular context, helicases facilitate the unwinding of the U1 snRNA stem 3 and therefore facilitate the binding of SRSF1, which would add another layer of regulation and would lead to a much higher affinitiy of SRSF1 for this RNA.

- The authors were asked to document the effect of adding a consensus 5' splice site at the end of GloC and BGSMN2. Data are shown now in Appendix Fig. S6 for BGSMN2, while data for GloC are not shown. These data show only a marginal improvement in splicing (one of two cases) and the text has been altered to acknowledge this. This is fine but does not really strengthen the overall conclusions of the manuscript.

We are not sure what the reviewer would consider a substantial improvement, but from IE's experience of splicing *in vitro* for 35 years this would generally be considered substantial.

- Nomenclature should be changed to U2AF2/1, and rather point to the old nomenclature when first introducing U2AF.

Respectfully, we disagree. We were asked in the previous round to harmonise our nomenclature either way, and this we did. We opted for U2AF35 and U2AF65 because the approximate molecular masses make it easier to remember which one is which and therefore which domains the proteins contain and their sites of binding.

Re: EMBOJ-2021-107640R1
Exon-independent recruitment of SRSF1 is mediated by U1 snRNP stem-loop 3

Dear Prof. Eperon,

Thank you for submitting your revised manuscript and addressing the remaining issues, as well as including the additional explanations with the available source data. I am pleased to say that we will now proceed with publication and would therefore ask you to address a small number of editorial and formatting issues that are listed in detail below. Once these remaining issues are resolved, we will be happy to formally accept the manuscript for publication in The EMBO Journal.

Thank you again for giving us the chance to consider your manuscript for The EMBO Journal. Please feel free to contact me if you have further questions regarding the revision or any of the specific points listed below.

Kind regards,

Stefanie

The authors have made all requested editorial changes.

3rd Revision - Editorial Decision 7th Oct 2021

Thank you again for submitting the final revised version of your manuscript for our consideration. I am pleased to inform you that we have now accepted it for publication in The EMBO Journal.

EMBO PRESS

YOU MUST COMPLETE ALL CELLS WITH A PINK BACKGROUND

PLEASE NOTE THAT THIS CHECKLIST WILL BE PUBLISHED ALONGSIDE YOUR PAPER

Corresponding Author Name: I.C. Eperon

Journal Submitted to: EMBO Journal

Manuscript Number: EMBOJ-2021-107640

Reporting Checklist For Life Sciences Articles (Rev. June 2017)

This checklist is used to ensure good reporting standards and to improve the reproducibility of published results. These guidelines are consistent with the Principles and Guidelines for Reporting Preclinical Research issued by the NIH in 2014. Please follow the journal's authorship guidelines in preparing your manuscript.

A- Figures

1. Data

The data shown in figures should satisfy the following conditions:

- the data were obtained and processed according to the field's best practice and are presented to reflect the results of the experiments in an accurate and unbiased manner.
- figure panels include only data points, measurements or observations that can be compared to each other in a scientifically meaningful way.
- graphs include clearly labeled error bars for independent experiments and sample sizes. Unless justified, error bars should not be shown for technical replicates.
- if n< 5, the individual data points from each experiment should be plotted and any statistical test employed should be justified
- Source Data should be included to report the data underlying graphs. Please follow the guidelines set out in the author ship guidelines on Data Presentation.

2. Captions

Each figure caption should contain the following information, for each panel where they are relevant:

- a specification of the experimental system investigated (eg cell line, species name).
- the assay(s) and method(s) used to carry out the reported observations and measurements
- an explicit mention of the biological and chemical entity(ies) that are being measured.
- an explicit mention of the biological and chemical entity(ies) that are altered/varied/perturbed in a controlled manner.

- the exact sample size (n) for each experimental group/condition, given as a number, not a range;
- a description of the sample collection allowing the reader to understand whether the samples represent technical or biological replicates (including how many animals, litters, cultures, etc.).
- a statement of how many times the experiment shown was independently replicated in the laboratory.
- definitions of statistical methods and measures:
  - common tests, such as t-test (please specify whether paired vs. unpaired), simple χ2 tests, Wilcoxon and Mann-Whitney tests, can be unambiguously identified by name only, but more complex techniques should be described in the methods section;
  - are tests one-sided or two-sided?
  - are there adjustments for multiple comparisons?
  - exact statistical test results, e.g., P values = x but not P values < x;
  - definition of 'center values' as median or average;
  - definition of error bars as s.d. or s.e.m.

Any descriptions too long for the figure legend should be included in the methods section and/or with the source data.

In the pink boxes below, please ensure that the answers to the following questions are reported in the manuscript itself. Every question should be answered. If the question is not relevant to your research, please write NA (non applicable). We encourage you to include a specific subsection in the methods section for statistics, reagents, animal models and human subjects.

USEFUL LINKS FOR COMPLETING THIS FORM

http://www.antibodypedia.com
http://1degreebio.org
http://www.equator-network.org/reporting-guidelines/improving-bioscience-research-repor

http://grants.nih.gov/grants/olaw/olaw.htm
http://www.mrc.ac.uk/Ourresearch/Ethicsresearchguidance/Useofanimals/index.htm
http://ClinicalTrials.gov
http://www.consort-statement.org
http://www.consort-statement.org/checklists/view/32-consort/66-title

http://www.equator-network.org/reporting-guidelines/reporting-recommendations-for-tumo

http://datadryad.org

http://figshare.com

http://www.ncbi.nlm.nih.gov/gap

http://www.ebi.ac.uk/ega

http://biomodels.net/

http://biomodels.net/miriam/
http://jjj.biochem.sun.ac.za
http://oba.od.nih.gov/biosecurity/biosecurity_documents.html
http://www.selectagents.gov/

B- Statistics and general methods

Please fill out these boxes (Do not worry if you cannot see all your text once you press return)

1.a. How was the sample size chosen to ensure adequate power to detect a pre-specified effect size?	The number of SM spots analysed was determined by the requirement to distinguish between binding of one or two molecules of SRSF1 or U1A. Splicing assays were performed using at least three biological replicates and NMR/ITC experiments were repeated at least one time.
1.b. For animal studies, include a statement about sample size estimate even if no statistical methods were used.	N/A
2. Describe inclusion/exclusion criteria if samples or animals were excluded from the analysis. Were the criteria pre-established?	The selection of Cy5 spots for analysis is based on the fit of their intensities to Gaussian parameters and the absence of overlapping spots, as decribed in the Statistics section of the Methods.
3. Were any steps taken to minimize the effects of subjective bias when allocating animals/samples to treatment (e.g. randomization procedure)? If yes, please describe.	N/A
For animal studies, include a statement about randomization even if no randomization was used.	N/A
4.a. Were any steps taken to minimize the effects of subjective bias during group allocation or/and when assessing results (e.g. blinding of the investigator)? If yes please describe.	No
4.b. For animal studies, include a statement about blinding even if no blinding was done	N/A
5. For every figure, are statistical tests justified as appropriate?	Yes.
Do the data meet the assumptions of the tests (e.g., normal distribution)? Describe any methods used to assess it.	SM frequencies evaluated as discrete data used in Chi square test.
Is there an estimate of variation within each group of data?	N/A

Is the variance similar between the groups that are being statistically compared?	N/A

C- Reagents

6. To show that antibodies were profiled for use in the system under study (assay and species), provide a citation, catalog number and/or clone number, supplementary information or reference to an antibody validation profile. e.g., Antibodypedia (see link list at top right), 1DegreeBio (see link list at top right).	Source and catalogue number specified.
7. Identify the source of cell lines and report if they were recently authenticated (e.g., by STR profiling) and tested for mycoplasma contamination.	HeLa and HEK293T cells. Not tested recently.

* for all hyperlinks, please see the table at the top right of the document

D- Animal Models

8. Report species, strain, gender, age of animals and genetic modification status where applicable. Please detail housing and husbandry conditions and the source of animals.	N/A
9. For experiments involving live vertebrates, include a statement of compliance with ethical regulations and identify the committee(s) approving the experiments.	N/A
10. We recommend consulting the ARRIVE guidelines (see link list at top right) (PLoS Biol. 8(6), e1000412, 2010) to ensure that other relevant aspects of animal studies are adequately reported. See author guidelines, under 'Reporting Guidelines'. See also: NIH (see link list at top right) and MRC (see link list at top right) recommendations. Please confirm compliance.	N/A

E- Human Subjects

11. Identify the committee(s) approving the study protocol.	N/A
12. Include a statement confirming that informed consent was obtained from all subjects and that the experiments conformed to the principles set out in the WMA Declaration of Helsinki and the Department of Health and Human Services Belmont Report.	N/A
13. For publication of patient photos, include a statement confirming that consent to publish was obtained.	N/A
14. Report any restrictions on the availability (and/or on the use) of human data or samples.	N/A
15. Report the clinical trial registration number (at ClinicalTrials.gov or equivalent), where applicable.	N/A
16. For phase II and III randomized controlled trials, please refer to the CONSORT flow diagram (see link list at top right) and submit the CONSORT checklist (see link list at top right) with your submission. See author guidelines, under 'Reporting Guidelines'. Please confirm you have submitted this list.	N/A
17. For tumor marker prognostic studies, we recommend that you follow the REMARK reporting guidelines (see link list at top right). See author guidelines, under 'Reporting Guidelines'. Please confirm you have followed these guidelines.	N/A

F- Data Accessibility

18: Provide a "Data Availability" section at the end of the Materials & Methods, listing the accession codes for data generated in this study and deposited in a public database (e.g. RNA-Seq data: Gene Expression Omnibus GSE39462, Proteomics data: PRIDE PXD000208 etc.) Please refer to our author guidelines for 'Data Deposition'.<br><br>Data deposition in a public repository is mandatory for:<br>a. Protein, DNA and RNA sequences<br>b. Macromolecular structures<br>c. Crystallographic data for small molecules<br>d. Functional genomics data<br>e. Proteomics and molecular interactions	The data supporting this publication are available from the University of Leicester's Figshare data repository https://leicester.figshare.com/account/home#/projects/116697. The single molecule image data in the Appendix is available upon request.
19. Deposition is strongly recommended for any datasets that are central and integral to the study; please consider the journal's data policy. If no structured public repository exists for a given data type, we encourage the provision of datasets in the manuscript as a Supplementary Document (see author guidelines under 'Expanded View' or in unstructured repositories such as Dryad (see link list at top right) or Figshare (see link list at top right).	Done
20. Access to human clinical and genomic datasets should be provided with as few restrictions as possible while respecting ethical obligations to the patients and relevant medical and legal issues. If practically possible and compatible with the individual consent agreement used in the study, such data should be deposited in one of the major public access-controlled repositories such as dbGAP (see link list at top right) or EGA (see link list at top right).	N/A
21. Computational models that are central and integral to a study should be shared without restrictions and provided in a machine-readable form. The relevant accession numbers or links should be provided. When possible, standardized format (SBML, CellML) should be used instead of scripts (e.g. MATLAB). Authors are strongly encouraged to follow the MIRIAM guidelines (see link list at top right) and deposit their model in a public database such as Biomodels (see link list at top right) or JWS Online (see link list at top right). If computer source code is provided with the paper, it should be deposited in a public repository or included in supplementary information.	No computational models are in the ms. The analysis of single molecules is described in detail.

G- Dual use research of concern

22. Could your study fall under dual use research restrictions? Please check biosecurity documents (see link list at top right) and list of select agents and toxins (APHIS/CDC) (see link list at top right). According to our biosecurity guidelines, provide a statement only if it could.	No.
